# Twistionics in halide perovskites

**DOI:** 10.1038/s41467-026-77126-7

**Published:** 2026-08-25

**Authors:** Sanxia Yin, Shihao Zhang, Weiqi Gao, Zhou Li, Yaonan Xiong, Xin Zhang, Pan Xu, Kun Zheng, Yangfeng Li, Jinhua Hong, Shuchen Zhang, Chao Ma, Lei Liao, Yiliu Wang, Shulin Chen

**Affiliations:** 1 National Key Laboratory of Power Semiconductor and Integration Technology, Engineering Research Center of Advanced Semiconductor Technology and Application of Ministry of Education, College of Semiconductors (College of Integrated Circuits), Hunan University, Changsha, 410082 China; 2 School of Physics and Electronics, Hunan University, Changsha, 410082 China; 3 College of Materials Science and Engineering, Hunan University, Changsha, 410082 China; 4 Key Laboratory of Precision and Intelligent Chemistry, Department of Materials Science and Engineering, School of Chemistry and Materials Science, University of Science and Technology of China, Hefei, China

**Keywords:** Electronic devices, Characterization and analytical techniques

## Abstract

Ion migration is a fundamental instability in halide perovskites, critically constraining their practical development in optoelectronic applications. Here we introduce twistionics, a twist-angle engineering strategy, to modulate ionic transport in halide perovskites. Through low-dose in situ aberration-corrected scanning transmission electron microscopy, we directly visualize the twist-angle-dependent ion diffusion in CsPbBr_3_-CsPbCl_3_ heterostructures. Our observations uncover a two-step migration pathway: formation of interfacial diffusion channels followed by their lateral growth to achieve complete interdiffusion. In lattice-aligned heterostructures, interfacial van der Waals (vdW) interactions facilitate the formation of diffusion channels and promote ion interdiffusion. Critically, introducing a twist angle disrupts interlayer registry, weakens vdW coupling, and dramatically suppresses cross-interface ion migration by reducing the density and continuity of diffusion channels. The resulting 28°-twisted heterostructures exhibit improved structural integrity and prolonged operational stability in photodetectors. These findings establish twist engineering as a powerful strategy for stabilizing perovskite devices and pave the way for regulating ion transport in the emerging field of twistionics.

## Abstract (web summary)

Using in situ atomic-scale STEM, Chen et al. show that twisting halide perovskite interfaces disrupts diffusion channels and slows ion migration and improve photodetector stability, advancing twistionics as an emerging route to control ion transport.

## Introduction

In recent years, metal halide perovskites (MHPs) have emerged as promising candidates in optoelectronic devices, including photovoltaic cells, light-emitting diodes, and photodetectors owing to their excellent optoelectronic properties^1–5^. However, their widespread applications is impeded by intrinsic instability, primarily arising from facile ion migration enabled by the soft lattice and high defect density of perovskites^6,7^. Ion migration in MHPs can trigger phase transitions, induce structural decomposition, and cause electrode corrosion, ultimately undermining device performance and lifetime^8,9^. In photovoltaics, it results in current-voltage hysteresis and efficiency roll-off^10,11^, while its effect on open-circuit voltage is complex and can be either negative or positive depending on device conditions^12–14^. In mixed-halide perovskites, it drives phase separation, destabilizing the bandgap^15^ and degrading luminescent purity and efficiency^16^. In photodetectors, ion migration contributes to large noise, baseline drift, and high dark current, impairing the performance of X-ray detection^17–19^. Addressing these challenges requires a deeper mechanistic understanding of ion migration to enable rational design of stable next-generation devices.

Extensive studies of ion migration in halide perovskites have relied on bulk-based techniques such as current-voltage, photoluminescence, X-ray photoelectron spectroscopy, optical microscope and computational modeling^20–24^. These approaches have yielded valuable macroscopic insights, including halide diffusivity^17^, diffusion pathways^25^, and factors governing ion migration^15,26^. However, they lack the spatial and temporal resolution required to capture the dynamic evolution of ionic processes at the nanoscale and atomic scale. In contrast, recent advances in in-situ transmission electron microscope (TEM) have enabled atomic-scale observations of ion migration in lithium ion batteries^27^, functional oxide ceramics^28,29^, catalysts^30^, and memristors^31^. Yet, for halide perovskites, atomic-scale visualization of ion transport remains elusive, largely due to their extreme sensitivity to electron-beam irradiation and the inherent difficulty of tracking halide ions in real time. Consequently, critical local processes such as the nucleation and growth of ionic channels and the role of interfacial lattice interactions remain poorly understood and experimentally unprobed.

Recently, twistronics, the deliberate control of interlayer twist angles in van der Waals (vdW) materials, has revealed unprecedented electronic phenomena, including magic-angle superconductivity, correlated insulating states, and tunable moiré band structures^32,33^. Similar twist engineering has recently been extended to freestanding oxide perovskites, where polar vortices^33^, ferroelectric vortex patterns^34^, charge disproportionation^35^, and twist-angle-dependent conductivity^36^ have been observed. Furthermore, achieving intimate gapless interfaces in such systems often requires annealing^37^, and screw-dislocation networks have been observed at twisted oxide interfaces^38^. Inspired by these advances, we propose twistionics: a twist-angle engineering paradigm aimed at modulating ionic transport at the atomic scale. By altering the lattice registry through twisting, we hypothesize that we can disrupt the formation and continuity of interfacial diffusion channels, thereby suppressing cross-interface ion migration without altering the material's intrinsic chemistry.

**Fig. 1:**
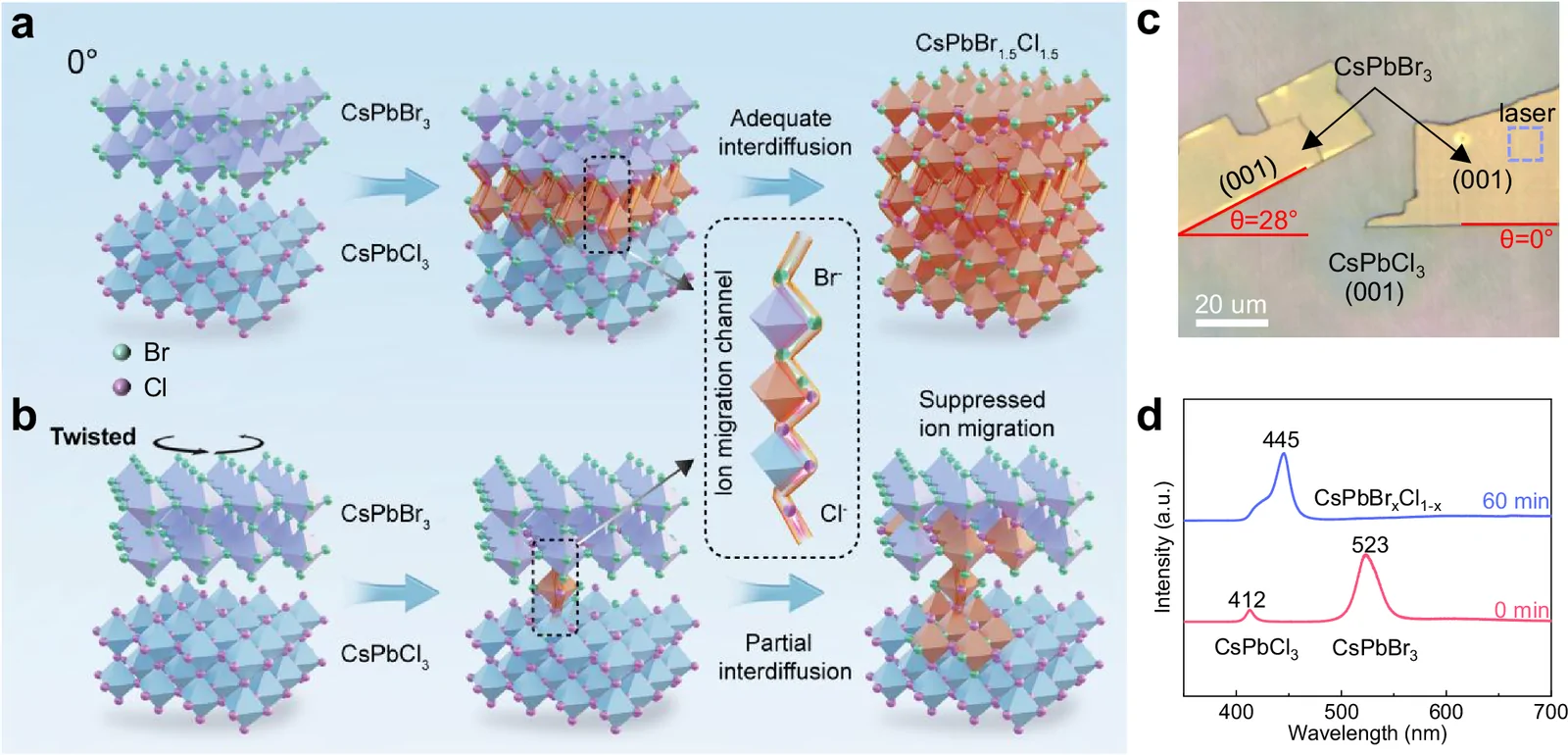
Twistionics to modulate ion migration in halide perovskites. **a, b** Schematic illustrations of ion migration dynamics in CsPbBr_3_-CsPbCl_3_ heterostructure with 0° and 28° twist angles. The enlarged section shows a schematic of the ion migration channel. The purple octahedra represent CsPbBr_3_, the light-blue octahedra represent CsPbCl_3_, and the orange octahedra represent the mixed-halide CsPbBr_1.5_Cl_1.5_ transition phase formed during interdiffusion. The green and purple spheres represent Br and Cl atoms, respectively. **c** Optical images of twisted heterostructure with different twist angles (0° and 28°). **d** PL spectra obtained from the heterostructure with twist angle 0° (Fig. 1c), heating at 150 °C for 0 min and 60 min.

In this work, we systematically investigate twistionics in halide perovskites using low-dose in situ aberration-corrected scanning transmission electron microscope (STEM). CsPbBr_3_-CsPbCl_3_ heterostructures with controlled twist angles serve as an ideal model system. Such kind of heterostructures with built-in electric fields can facilitate carrier separation, improving the performance of photovoltaic^39^ and photodetectors^19,40,41^. We directly visualize twist-angle-dependent ion diffusion in CsPbBr_3_-CsPbCl_3_ heterostructures and uncover a previously unreported two-step migration mechanism: (i) channel formation mediated by lattice alignment and vdW coupling, followed by (ii) lateral growth to achieve complete interdiffusion (Fig. 1a). Strikingly, initial perovskite nanobridges tend to be formed where the lattice of CsPbBr_3_ matches that of CsPbCl_3_, suggesting aligned lattice can facilitate such ion migration because interfacial vdW interactions serve as the driving force. Molecular dynamics (MD) simulations confirm that introducing a twist angle weakens these vdW interactions. Leveraging this insight, we engineered twisted heterostructures and found that a 28° twist configuration significantly delays diffusion channel formation and effectively suppresses cross-interface ion migration (Fig. 1b). The resulting twisted heterostructures exhibit enhanced structural and operational stability in photodetectors. These results provide fundamental insights into the mechanism of the twist-governed ion migration in perovskite heterostructures and highlight the potential of twist-angle-engineering for designing high-performance perovskite-based optoelectronic devices.

## Results

### Twist-angle-dependent interdiffusion of halide ions

**Fig. 2:**
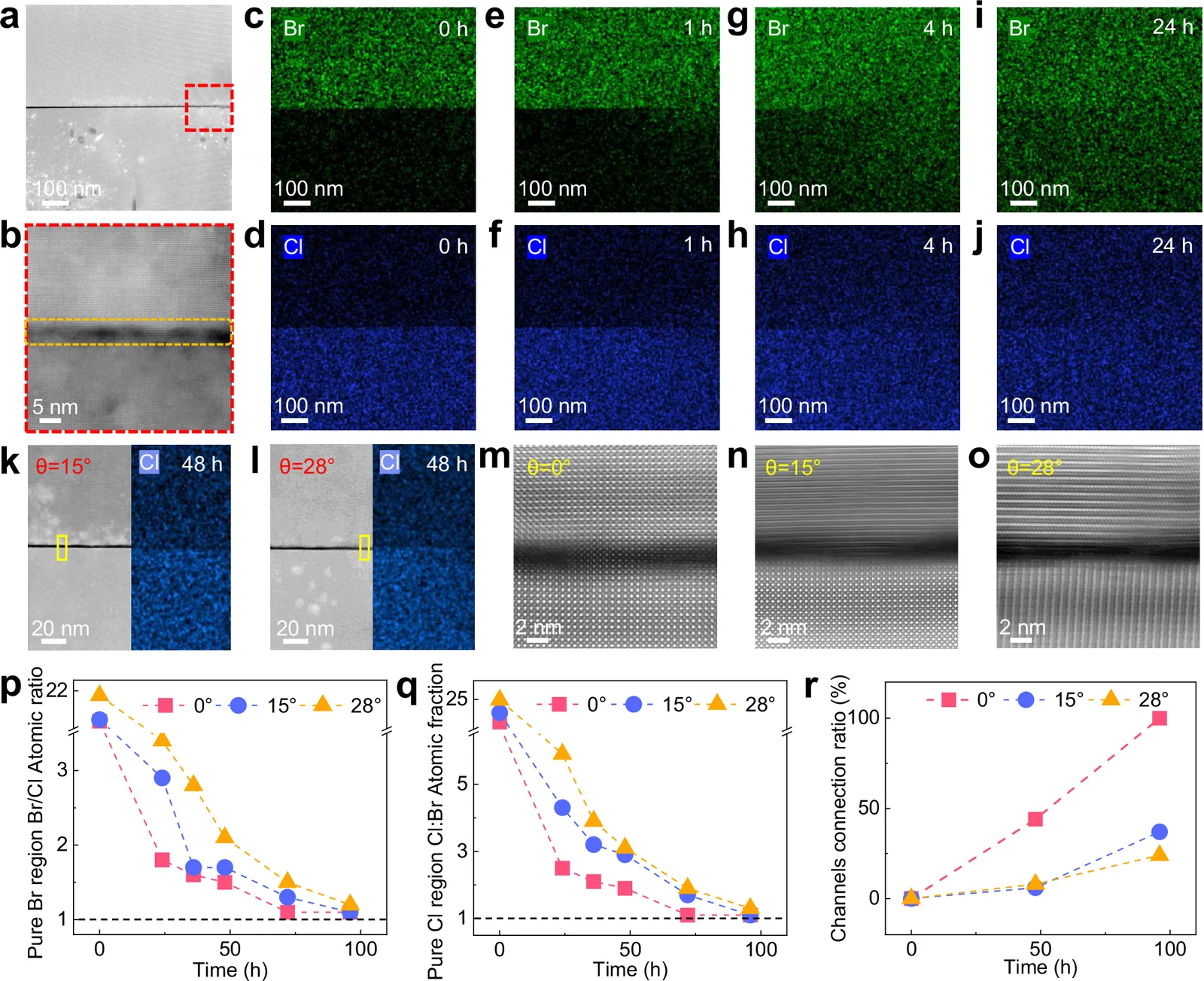
Twist-angle-dependent halide composition evolution and diffusion channels in CsPbCl_3_-CsPbBr_3_ heterostructures. **a, b** HAADF images of the diffusion channels in a twist angle of 0°. **c–j** The corresponding EDS mapping of Br and Cl at 0, 1, 4, and 24 h. **k, l** HAADF images and corresponding EDS mapping of Cl of the heterostructures with twist angles of 15° and 28° after 48 hours. The yellow rectangles indicate diffusion channels. **m–o** HAADF images of heterointerface with twist angles 0°, 15°, and 28°. **p, q** Compositional evolutions of the atomic ratio of Cl to Br at twisted heterostructures under 150 °C heating from pure Cl region and pure Br region. **r** The evolutions of diffusion channel connection ratio for different twist angles 0°, 15°, and 28°.

We have constructed a CsPbBr_3_-CsPbCl_3_ heterostructure with 0° and 28° twist-angle based on single-crystal CsPbBr_3_ and CsPbCl_3_ films (Fig. 1c and Fig. S1). The photoluminescence (PL) spectrum in Fig. 1d indicated the presence of interlayer ion migration between perovskite films. For the 0° twisted heterostructure, Figure 2a, b shows high angle annular fark field (HAADF) images of the diffusion channels. Fig. 2c–j presents the time evolution of Br and Cl energy dispersive x‑ray spectroscopy (EDS) maps at 0, 1, 4, and 24 hours. At 0 h, Br and Cl remain confined to their respective sides. After 1 h, weak interdiffusion appears around the diffusion channels highlighted by the red dashed square in Fig. 2a, while the rest of the interface shows no diffusion. At 4 h, interdiffusion intensifies and spreads laterally. By 24 h, Br and Cl become uniformly distributed within and around the channels. These observations demonstrate that halide ions preferentially migrate along these nanoscale diffusion channels rather than randomly across the entire interface.

To understand the origin of diffusion-channel formation, we examined the initial interface. The two perovskite crystals were initially separated by a narrow gap of 4 nm (Fig. S2a), which was rich in Br and Cl elements (80% at.) but depleted in Cs and Pb (20% at.) (Fig. S3). First-principles calculations show that the diffusion barrier from the bulk to the surface/gap (2.287 eV) is relatively lower than that for crossing the gap (3.133 eV) or from surface to bulk (2.574 eV) (Fig. S4). These results indicate that halide ions first diffuse from the bulk and accumulate in the interfacial gap, which has also been observed in Fig. S5. Over time, the halide-rich gap gradually evolves into perovskite nanobridges (Fig. S2b). Cation migration from the neighboring crystals compensates the local Cs/Pb depletion, enabling anion-cation migration and interfacial fusion^42,43^. This process requires interfacial coupling, as an isolated perovskite nanosheet without an adjacent crystal shows negligible ion migration (Fig. S6). With further interdiffusion, the interface continued to grow, and the amorphous region gradually crystallized, resulting in a complete interfacial fusion (Fig. S2c and Fig. S7). Fast Fourier transform (FFT) intensity profiles further confirm the lattice evolution from initial CsPbBr_3_/CsPbCl_3_ to mixed-halide CsPbBr_1.5_Cl_1.5_ (Fig. S8).

We further compared the interdiffusion of halide ions at twist angles of 15° and 28°. The interdiffusion behaviors after 48 h are shown in Fig. 2k, l. High-magnification images of the regions marked by the yellow rectangles are provided in Fig. S9. Figure 2m–o presents atomic-resolution images of the diffusion channels for the three twist angles. The ion diffusion channels were reduced at both twist angles, leading to slower ion migration. It is observed that 0°-twisted heterostructure quickly transformed into the full-diffusion product CsPbCl_1.5_Br_1.5_ within approximately 72 h, while the 28°-twisted sample exhibited delayed conversion time as large as 96 h. For the 15°-twisted configuration, we consider it achieved complete transformation between 72-96 h, as shown in Fig. 2p, q. This suggests that introducing a twist of 28° can decrease the diffusion of halide ions by approximately 25%. Meanwhile, statistical analysis of diffusion channels connection ratio based on experimental TEM images across the whole interface demonstrated the highest ratio at 0° and the lowest at 28°, indicating 28° twist angle reduces channel ratio by over 60% compared to 0° configuration (Fig. 2r). These findings suggest that a 28° twist angle between perovskite layers effectively suppresses cross-interface halide interdiffusion by significantly reducing the interfacial diffusion channels. In contrast, such cross-interface halide interdiffusion was not observed in CsPbBr_3_-SrTiO_3_ heterojunction (Fig. S10) while the CsPbBr_3_/NaCl heterostructure exhibited asymmetric halide migration: Cl^-^ migrated into CsPbBr_3_, whereas Br^-^ did not enter NaCl^44^ (Fig. S11). These results indicate that lattice matching alone is insufficient to enable mutual halide interdiffusion; chemical compatibility between the two lattices is also required.

### Atomic-scale observation of twist-angle-dependent ion diffusion pathways

**Fig. 3:**
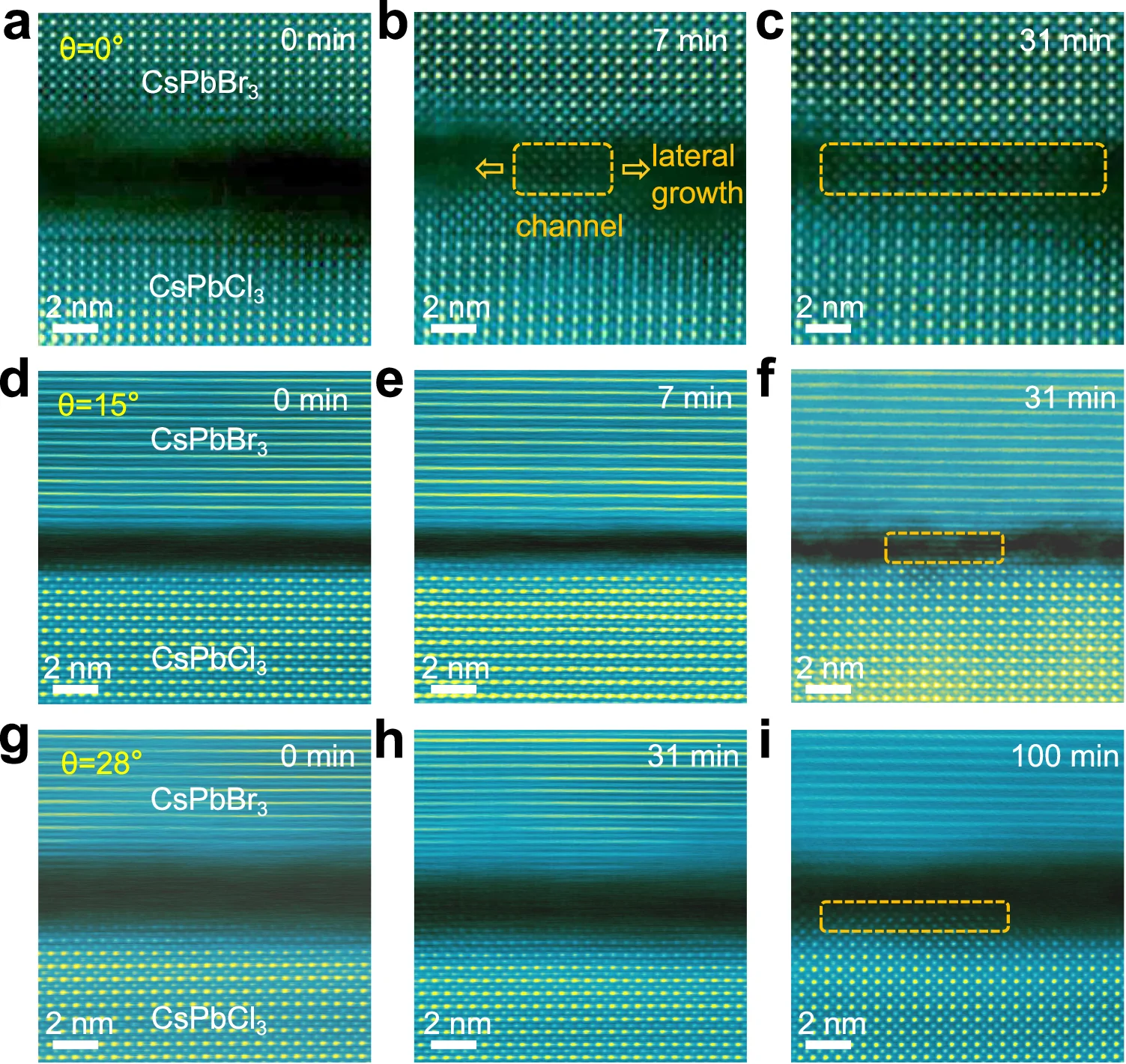
Atomic-scale visualization of twist-angle-dependent ion migration pathways. **a–i** Time-series in-situ HAADF STEM images of interfacial diffusion channel dynamics in the heterostructures with (**a–c**) 0°, **d–f** 15°, and **g–i** 28° twist angles under electron beam irradiation. The yellow dashed rectangles indicate the channel formation pathway. The images are presented with false color to enhance visual contrast between different regions.

To further elucidate atomic-scale mechanism of how diffusion channel form during the heterostructure fusion, we conducted atomic-scale observations of interface growth with different twist angles (0°, 15°, 28°) by in situ STEM. Due to the lattice difference between CsPbBr_3_ and CsPbCl_3_, their lattices present aligned and unaligned features, as indicated in Fig. S12. It is observed that the diffusion channels prefer to be formed where the lattice of CsPbBr_3_ aligns well to that of CsPbCl_3_. Herein, the lattice-matched regions include both fully aligned and half-unit-cell shifted configurations (Fig. S13). Figure 3a–i shows atomic-scale in situ observations of heterointerface growth dynamics with different twist angles, considering that electron beam irradiation can transfer energy to atoms to drive ion migration and interface fusion, similar to heat- or light-induced transitions^45^. At the initial status, the interface with a twist angle of 0° was not connected (Fig. 3a). After 7-min electron beam irradiation, the interface began to partially connect (Fig. 3b), forming diffusion channels. With further irradiation at 31 min, these channels gradually grew laterally (Fig. 3c) while retaining the perovskite phase (Fig. S14). In contrast, the 15°-twisted interface exhibited delayed channels formation, requiring 31 min of irradiation to develop partial channels (Fig. 3d–f) while the 28°-twisted interface just grew one atomic layer without forming channels even after 100-min electron beam irradiation (Fig. 3g–i). The delay in channel formation correlates directly with our measured interdiffusion kinetics (Fig. 2p, q), suggesting twist-angle-induced lattice misalignment effectively suppresses cross-interface ion migration. Control experiments confirmed that the faster interdiffusion kinetics of the 0° sample are retained despite variations in interfacial roughness and initial gap width (Figs. S13 and S15), reinforcing the dominant role of twist angle.

### Atomic-scale mechanism of twist-suppressed ion diffusion

**Fig. 4:**
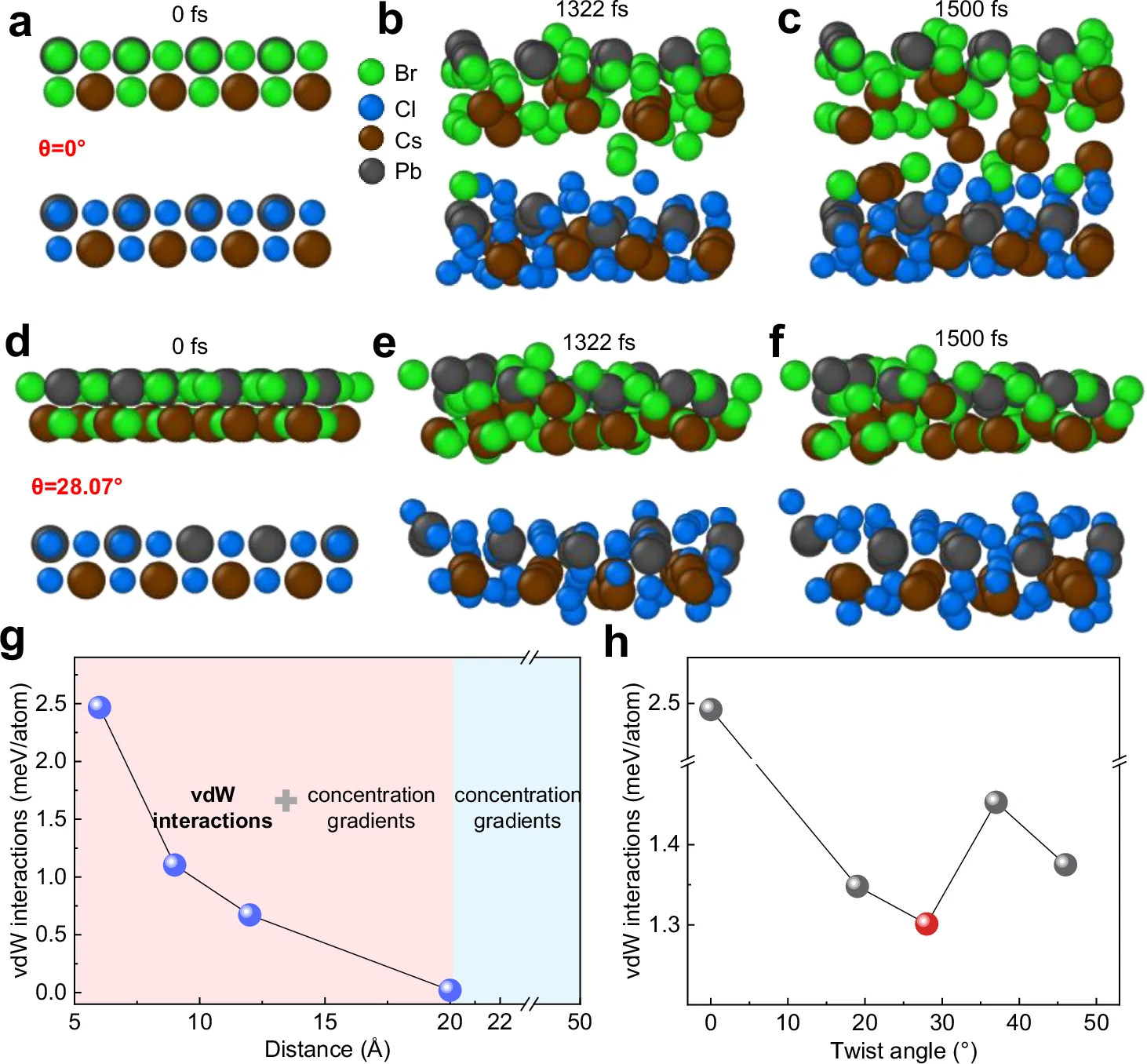
MD simulations of ion migration at twisted heterostructures. **a–c** MD simulations of ion diffusion in 0°-twisted heterostructure. **d–f** MD simulations of ion diffusion in 28.07°-twisted heterostructure. **g** Evolution of interfacial vdW interactions with varying distances of interfacial gap. **h** Evolution of vdW interactions with different twist angles at a fixed interlayer gap of 6 Å. The red data point indicates the 28° twist angle, at which the vdW interaction reaches its minimum.

Through MD simulations, we further investigated the diffusion dynamics at twisted heterostructures and elucidated the underlying mechanism of ion migration. MD simulations of the 0° CsPbBr_3_-CsPbCl_3_ heterostructure demonstrated a partial diffusion at 1322 fs and obvious interdiffusion at 1500 fs (Fig. 4a–c). In contrast, the diffusion process at a 28.07° twist angle is much slower with almost no diffusion at 1322 fs and only a slight diffusion by 1500 fs (Fig. 4d–f). This twist-angle-dependent suppression has also been confirmed by additional MD simulations using Cs-X terminations (Fig. S16). Since vdW interactions are sensitive to both interfacial distance and atomic registry, twist-induced lattice misalignment can modulate interfacial vdW coupling^46–48^ that usually increase as the interfacial distance decreases^49–51^, we further calculated the vdW interactions at the interface to clarify how twist angle affects interfacial coupling and ion interdiffusion^52^.

There is almost no vdW interaction when the interfacial gap exceeds 20 Å while it gradually increases as the distance of the gap decreases and substantially increases as the gap narrows to below 10 Å (Fig. 4g). This increase in vdW interactions promotes nanobridge formation, which then provides connected pathways for cross-interface halide interdiffusion. Accordingly, we propose a distance-dependent three-stage mechanism for ion interdiffusion: (1) When the interfacial gap exceeds 20 Å, the ion migration is dominated by concentration gradients^20,21,23^; (2) As the gap narrows to below 20 Å, vdW interactions gradually promote the ion migration^48^; (3) When the distance decreases to the lattice of the perovskites approximately 6 Å, spontaneous perovskite nanobridges are formed to serve as the channel for ion migration and achieve sufficient interdiffusion of the heterostructure (Fig. 3). Furthermore, we calculated vdW interactions with different twist angles (0°, 18.92°, 28.07°, 36.87° and 43.6°), which are commensurate angles allowed by periodic boundary conditions (Table S1). The result showed that introducing a twist angle can efficiently weaken vdW interactions, which decreased almost a half at a 28.07° twist angle (Fig. 4h), suggesting that a 28.07°-twisted heterostructure is likely to efficiently suppress cross-interface ion migration.

### Twist engineering enhanced stability in heterostructure devices

**Fig. 5:**
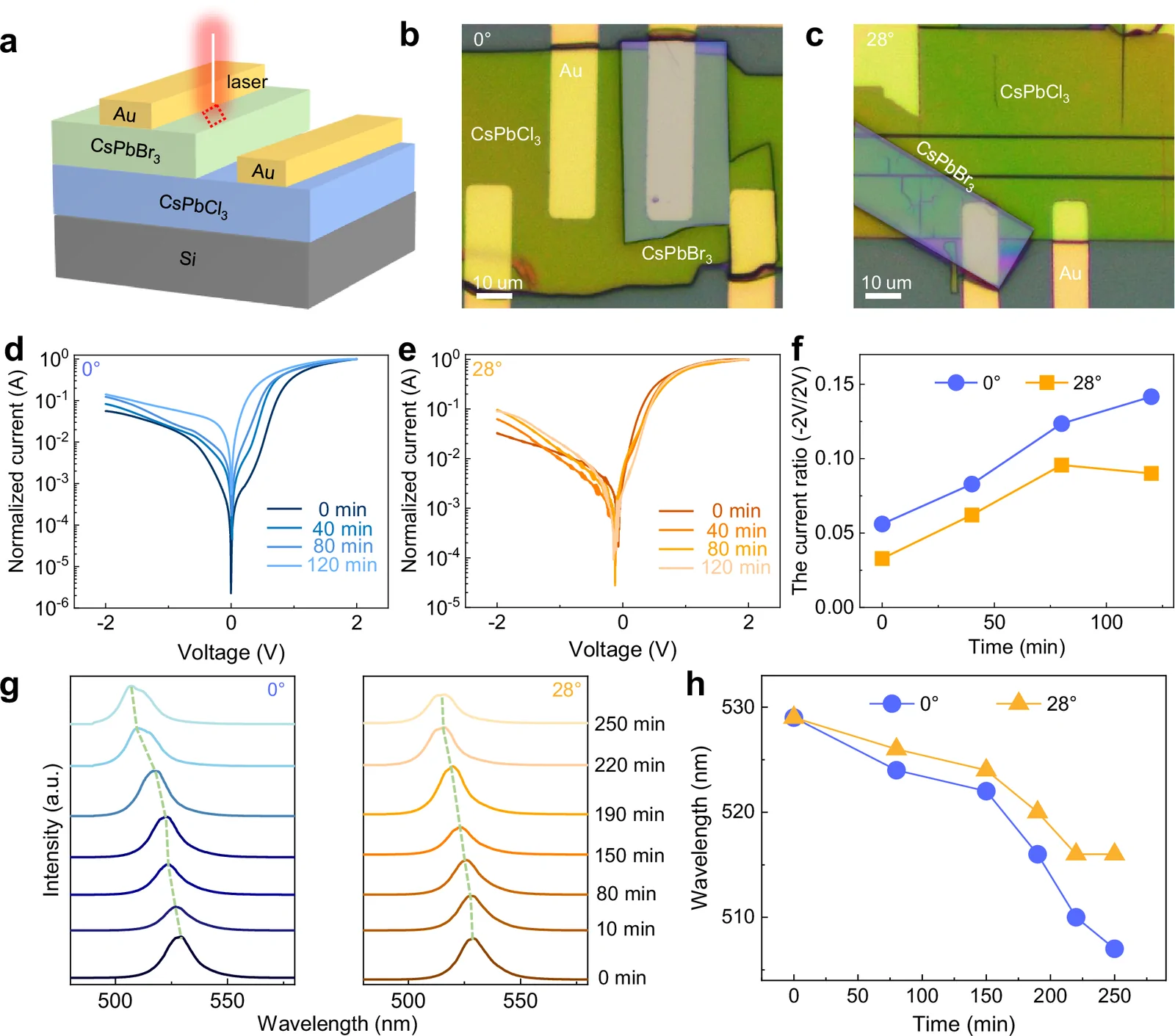
Enhanced operational stability of photodetectors via twist engineering. **a** Schematic diagram of the CsPbBr_3_-CsPbCl_3_ heterostructure photodetector. The dotted square indicates the position where the PL spectra were acquired. **b, c** Optical images of fabricated devices with 0° and 28° twist angles. **d, e** I-V curves of the 0° and 28° heterostructure devices heating at 150 °C for 0, 40, 80, and 120 min. The data in d and e are normalized to the maximum value in each dataset. **f** The corresponding evolution of the current ratio (−2V/ + 2 V) acquired from the I-V curves in (**d**, **e**). **g** The evolutions of PL spectra obtained from the CsPbBr_3_ side (Fig. 5a) under 150 °C heating. **h** The corresponding evolutions of the PL peak position acquired from the spectra in g.

To evaluate the impact of twist-suppressed ion migration in practical devices, we constructed CsPbBr_3_-CsPbCl_3_ heterostructure photodetectors with 0° and 28° configurations. Figure 5a illustrates the schematic structure of the heterostructure photodetector. Figure 5b, c present optical images of the 0° and 28° devices, respectively. The device was heated to 150 °C to accelerate halide-ions interdiffusion, allowing measurable interdiffusion within a practical time window. Although the absolute interdiffusion rates differ from those under imaging and operating conditions, the trend that twisted interfaces suppress cross-interface halide exchange likely remains consistent. The electrical performance was characterized through current-voltage (I-V) measurements after heating at 150 °C for various durations. For the 0° device, an obvious rectifying behavior of the heterostructure is observed at 0 min, which gradually weakened during the heating, indicating severe ion migration-induced interdiffusion (Fig. 5d). In contrast, the 28° device maintained significantly better rectification characteristics under identical conditions (Fig. 5e), confirming the effectiveness of twist engineering in preserving interfacial stability. The enhanced performance was further quantified by plotting the current ratio at −2 V and 2 V (Fig. 5f), which demonstrates better retention of rectification behavior in the 28° device. In addition, PL measurements under identical thermal activation further corroborated these trends: 0° heterostructure exhibited a fast blueshift (Δλ = 22 nm at 260 min), while the 28° configuration showed a slow response (Δλ = 13 nm at 260 min) as shown in Fig. 5g, h and Table S2. Although real devices might not contain an initial 4 nm gap^40^, efficient cross-interface halide exchange still requires connected interfacial diffusion pathways; thus, twisting remains relevant by weakening interfacial registry and suppressing the formation and lateral continuity of these pathways. These device-level results directly correlate with our atomic-scale observations and MD simulations, demonstrating that twist engineering is an effective strategy for suppressing cross-interface ion migration and significantly improving the operational stability of perovskite heterostructure devices.

## Discussion

In heterostructure photovoltaics and photodetectors, halide-ion migration driven by chemical potential is inevitable and reaches micrometer-scale^19,40,41^. These uncontrolled ion migrations result in large noise, high dark current, and decreased detector limit, greatly impairing device performance and stability^53^. Our in situ STEM observations uncover the atomic-scale mechanism governing ion migration at CsPbBr_3_-CsPbCl_3_ heterostructures, and clarify the relationship between the micro-nanostructure of halide perovskites and ion migration dynamics. These findings substantially enhance the understanding of the failure mechanism in heterostructure devices. In addition, we observed interfacial gaps at CsPbBr_3_-CsPbCl_3_ heterostructures, a feature also present in 2D-3D perovskite heterostructures^54^. Although simulations suggest such perovskite heterojunctions could remain stable for several years^40,41^, our experimental results demonstrate that a CsPbCl_3_-CsPbBr_3_ heterostructure can evolve into CsPbCl_x_Br_3-x_ with uniform composition within 96 hours (Fig. 2). This rapid ion interdiffusion is likely accelerated by environmental factors such heating, electrical bias, and illumination^10^.

Suppressing cross-interface ion migration has long been considered critical for enhancing the stability of optoelectronic devices^10,55^. Traditional approaches have employed composition and additive engineering^56,57^ and diffusion-blocking layers such as graphene (Fig. S17) and 2D perovskites^58,59^. Distinct from these strategies, our twist-angle engineering provides a purely geometric route to suppress cross-interface halide interdiffusion, without altering material composition^58^ or introducing foreign interlayers^57^. This approach may also be applicable to stabilizing 2D/2D, 2D/3D, and 3D/3D perovskite heterostructures. Moreover, the proposed twistionics also opens up promising avenues for regulating ion migration in lithium ion batteries, memory devices, and catalytic, thereby broadening the scope of twist engineering beyond its traditional applications in twistronics and twist-mechanics^60–63^.

In summary, we introduce and establish twistionics as a structural engineering strategy for controlling ionic transport in halide perovskites. Through in situ aberration-corrected STEM, we achieve direct observation of twist-angle-dependent ion diffusion in CsPbBr_3_-CsPbCl_3_ heterostructures, revealing a two-step migration pathway: formation of diffusion channels and gradual lateral growth to achieve complete ion interdiffusion. Interestingly, an aligned lattice facilitates such ion migration, as interfacial vdW interactions serve as the driving force. In contrast, introducing a twist angle between two perovskite layers can weaken these vdW interactions and reduce channel densities, thereby effectively suppressing cross-interface ion migration and enhancing the stability of heterostructure photodetectors. This work not only elucidates a twist-angle-governed ionic diffusion mechanism at the atomic scale in halide perovskite heterostructures, but also establishes twisted-heterostructure engineering as a broadly applicable paradigm towards next-generation, highly stable functional devices.

## Methods

### Epitaxial growth of perovskite thin films

CsPbBr_3_ monocrystalline films were epitaxially grown on muscovite mica substrates through a vapor-phase deposition process. During this process, a mixture of CsBr and PbBr_2_ precursors were positioned at the center of the tube furnace, with the mica substrate placed downstream. The argon carrier gas flowed at a rate of 100 sccm, and the growth chamber was maintained at approximately 0.15 atm. The temperature was raised to 600 °C and held steady for 20 min to ensure complete growth.

CsPbCl_3_ monocrystalline films were epitaxially grown on SrTiO_3_ (STO) substrates, which were pretreated with oxygen plasma for 10 minutes to enhance their hydrophilic properties. The mixed precursors of CsCl and PbCl_2_ were placed at the center of the tube furnace, with the STO substrates downstream. The pressure inside the chamber was maintained at around 0.09 atm, with an argon flow rate of 100 sccm. The growth temperature was set to 640 °C and held for 30 min to obtain CsPbCl_3_ films.

### Halide perovskite heterostructure synthesis and device fabrication

The CsPbCl_3_ film was first transferred onto a thermally oxidized SiO_2_/Si substrate via a van der Waals transfer method. Subsequently, the CsPbBr_3_ film was peeled off from mica using Thermal Release Tapes (TRT) (90–100 °C) and transferred onto a PDMS surface. The PDMS-supported CsPbBr_3_ film was then carefully stacked onto the CsPbCl_3_/SiO_2_/Si surface to form the heterostructure. The twist angle between the two films was controlled by aligning their edges under an optical microscope. After gentle heating at 110 °C for 3 min, the TRT was released, and the PDMS was removed, leaving the CsPbBr_3_ film on top of CsPbCl_3_.

The preformed gold electrodes coated with PMMA on a sacrificial silicon substrate were lifted using PDMS. The electrodes were then precisely transferred to a specific location on a heterojunction. Subsequently, the electrode pad region was subjected to electron beam exposure and developed, exposing the electrode area for the electrical contact.

### Density functional calculations

We carried out first-principles calculations within the framework of the generalized gradient approximation functional^64^ of the density functional theory through employing the Vienna ab initio simulation package (VASP)^65^ with projector augmented wave method^66^. The MD simulation was performed within a canonical ensemble at 300 K. We calculated the vdW interactions by $\Delta E=\left({E}_{1}+{E}_{2}-{E}_{{total}}\right)/{N}_{{atom}}$ in which ${E}_{1}$ and ${E}_{2}$ refer to the energy of isolated CsPbCl_3_ layers and CsPbBr_3_ layers, respectively. ${E}_{{total}}$ represents the total energy of heterostructure, and ${N}_{{atom}}$ is the total number of atoms in the twisted heterostructure. Thus, larger ΔE indicates of stronger vdW interactions between layers at the interface.

### Data acquisition and analysis

STEM images were obtained using an aberration-corrected FEI (Themis Z) at 300 kV with a beam current of ~5–45 pA, and a convergence semi-angle of 25 mrad. STEM EDS mapping was obtained with a beam current of ~25 –45 pA. Specific imaging conditions for each figure are detailed as follows: Fig. 2: (a–i) EDS mapping (35 pA); (j–l) HAADF-STEM images acquired at 12 pA, 23 pA, and 24 pA, respectively; Fig. 3: (a–i) HAADF-STEM images (15 pA); TEM samples of heterostructures were prepared by focused ion beam using a Thermo Scientific Helios 5 CX. PL spectra were measured using a Raman spectrometer (inVia Qontor, Renishaw) with a 496 nm laser as the excitation source. I-V measurements were conducted using a Lakeshore PS-100 cryogenic probe station equipped with a Keithley 2400 sourcemeter. The measurements were performed under vacuum at room temperature. The voltage was swept from −2V to +2 V with a step of 6 mV.

## Supplementary information

Supplementary Information

Transparent Peer Review file

## Data Availability

All relevant data supporting the findings of this study are included in the paper and Supplementary Information files. The source data underlying the figures in the main text have also been deposited in the public repository Zenodo and are openly accessible at https://doi.org/10.5281/zenodo.21712309. Additional information is available from the corresponding author upon request. Source data are provided with this paper.
