# Supplementary material for: Twistionics in halide perovskites
Source: Nat Commun. 2026 Aug 25;17:10177. doi: 10.1038/s41467-026-77126-7 (PMC13612815; doi:10.1038/s41467-026-77126-7)
Supplement: Supplementary file 1 [file 41467_2026_77126_MOESM1_ESM.pdf]

Supplementary Information for

Twistionics in halide perovskites

Sanxia Yin[a, #], Shihao Zhang[b, #], Weiqi Gao[b, #], Zhou Li[a, #], Yaonan Xiong[a], Xin Zhang[c], Pan Xu[c], Kun Zheng[a], Yangfeng Li[a], Jinhua Hong[c], Shuchen Zhang[d], Chao Ma[c, *], Lei Liao[a, *], Yiliu Wang[b,*], Shulin Chen[a, *]

[a]National Key Laboratory of Power Semiconductor and Integration Technology, Engineering Research Center of Advanced Semiconductor Technology and Application of Ministry of Education, College of Semiconductors (College of Integrated Circuits), Hunan University, Changsha 410082, China

[b]School of Physics and Electronics, Hunan University, Changsha, 410082, China

[c]College of Materials Science and Engineering, Hunan University, Changsha, 410082, China

[d]Key Laboratory of Precision and Intelligent Chemistry, Department of Materials Science and Engineering, School of Chemistry and Materials Science, University of Science and Technology of China, Hefei, China

*Correspondence and requests for materials should be addressed to: chenslin@hnu.edu.cn (S.L. Chen); yiliuwang@hnu.edu.cn (Y.L. Wang); liaolei@hnu.edu.cn (L. Liao); cma@hnu.edu.cn (C. Ma)

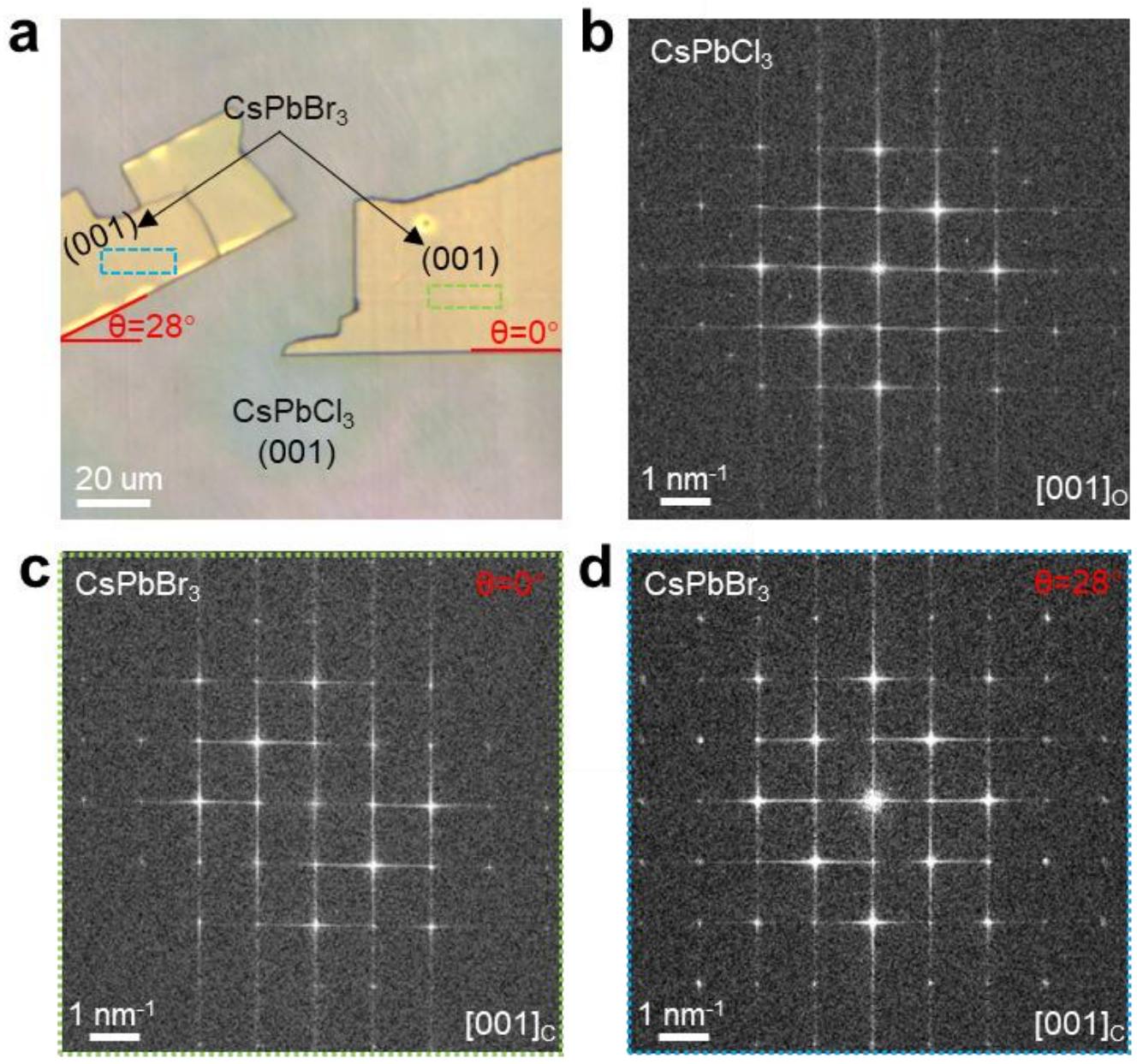

Supplementary Figure 1 | Crystallinity and orientation of $CsPbBr_3$-$CsPbCl_3$ heterostructure. (**a**) Optical images of twisted the heterostructure with different twist angles (0° and 28°). The FFT pattern (**b**) along the $[001]_O$ direction of orthogonal $CsPbCl_3$ and (**c, d**) along the $[001]_C$ direction of cubic $CsPbBr_3$ with twist angles 0° and 28°.

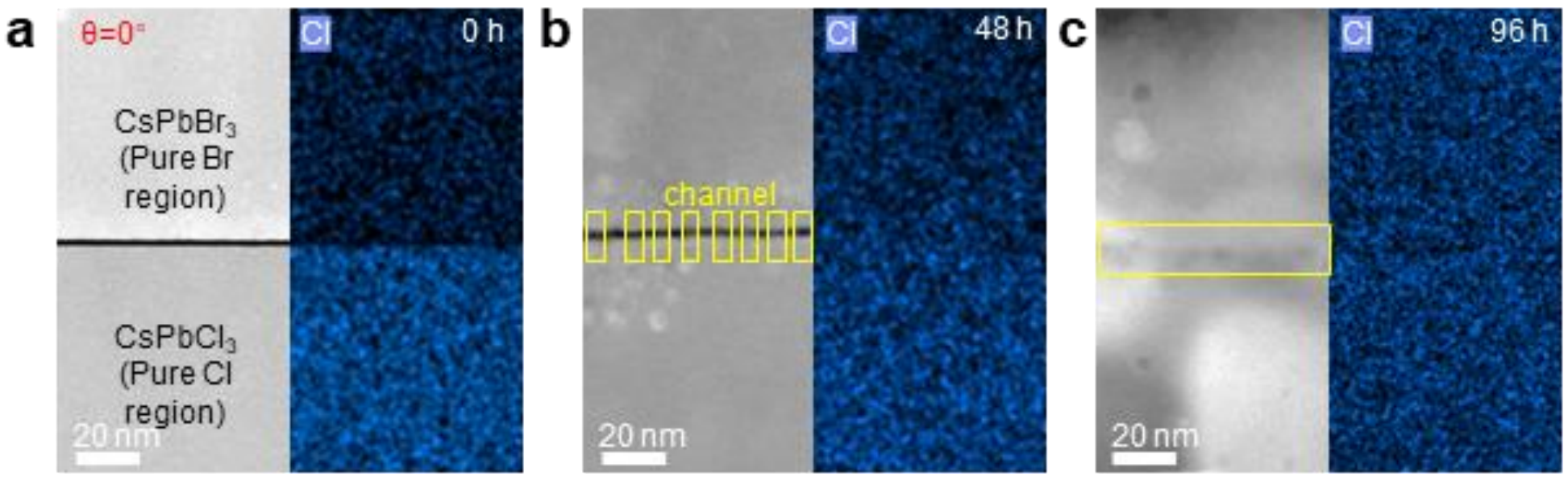

Supplementary Figure 2 | HAADF images and corresponding EDS mapping of Cl of the heterostructures with twist angle of 0° after (a) 0, **(b)** 48, and **(c)** 96 hours. The yellow dashed rectangles indicate diffusion channels.

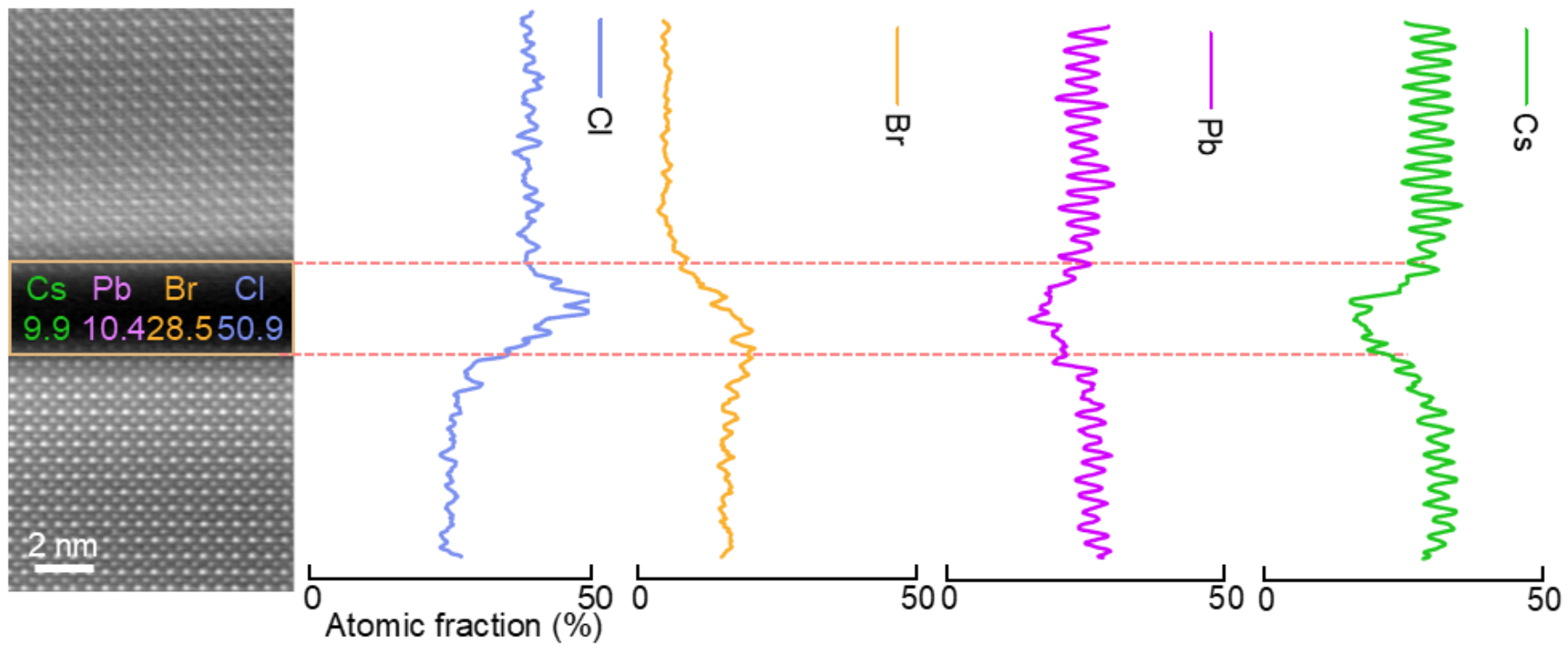

Supplementary Figure 3 | HAADF image and quantitative element analysis at the initial heterointerface with twist angle of 0°.

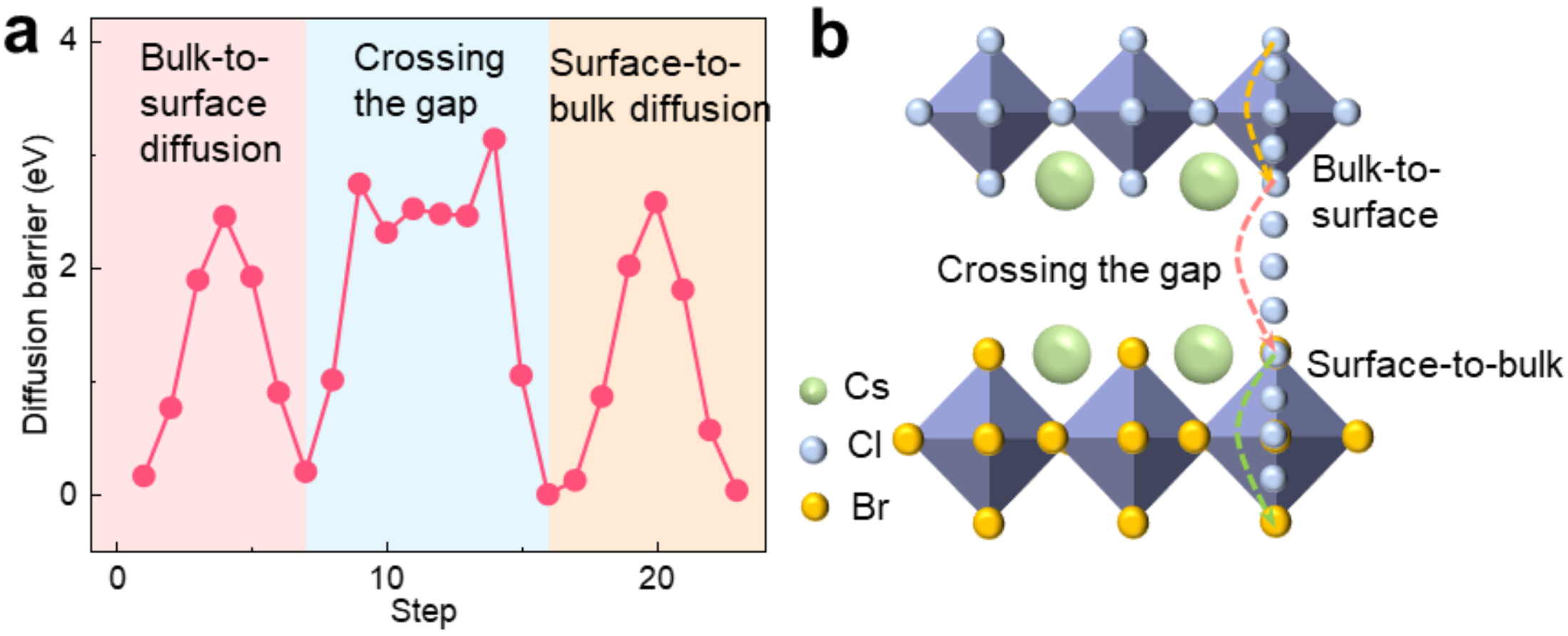

Supplementary Figure 4 | Energy profile and schematic illustration of Cl diffusion across the $CsPbCl_3$/$CsPbBr_3$ heterostructure. (a) Calculated energy barriers for Cl diffusion in three stages. **(b)** Schematic representation of the three-stage diffusion pathway, indicating the bulk-to-surface, surface-to-surface (crossing the gapping), and surface-to-bulk processes.

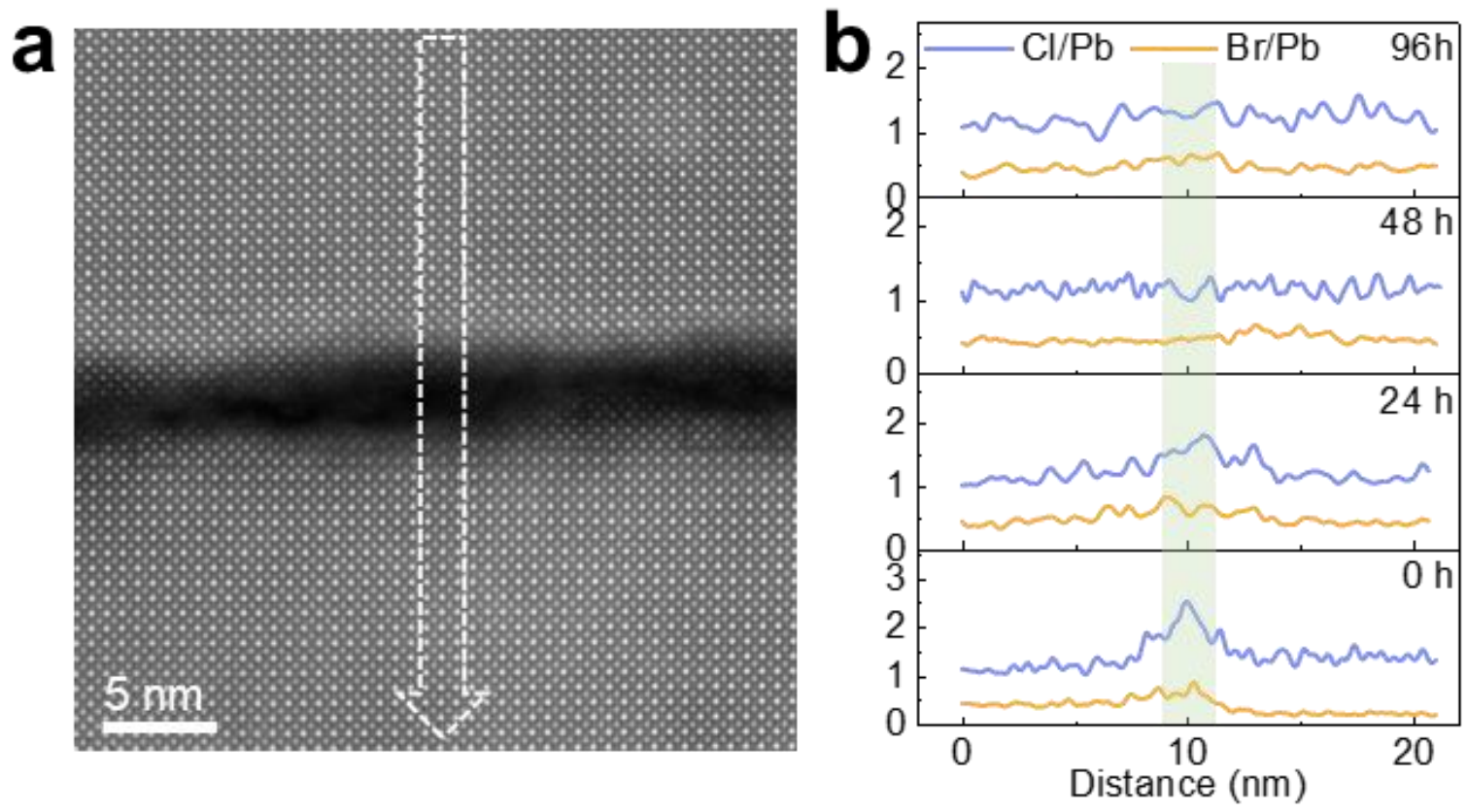

Supplementary Figure 5 | The evolution of atomic-ratio for Cl/Pb and Br/Pb near the 0° heterointerface. The green background highlights the interface.

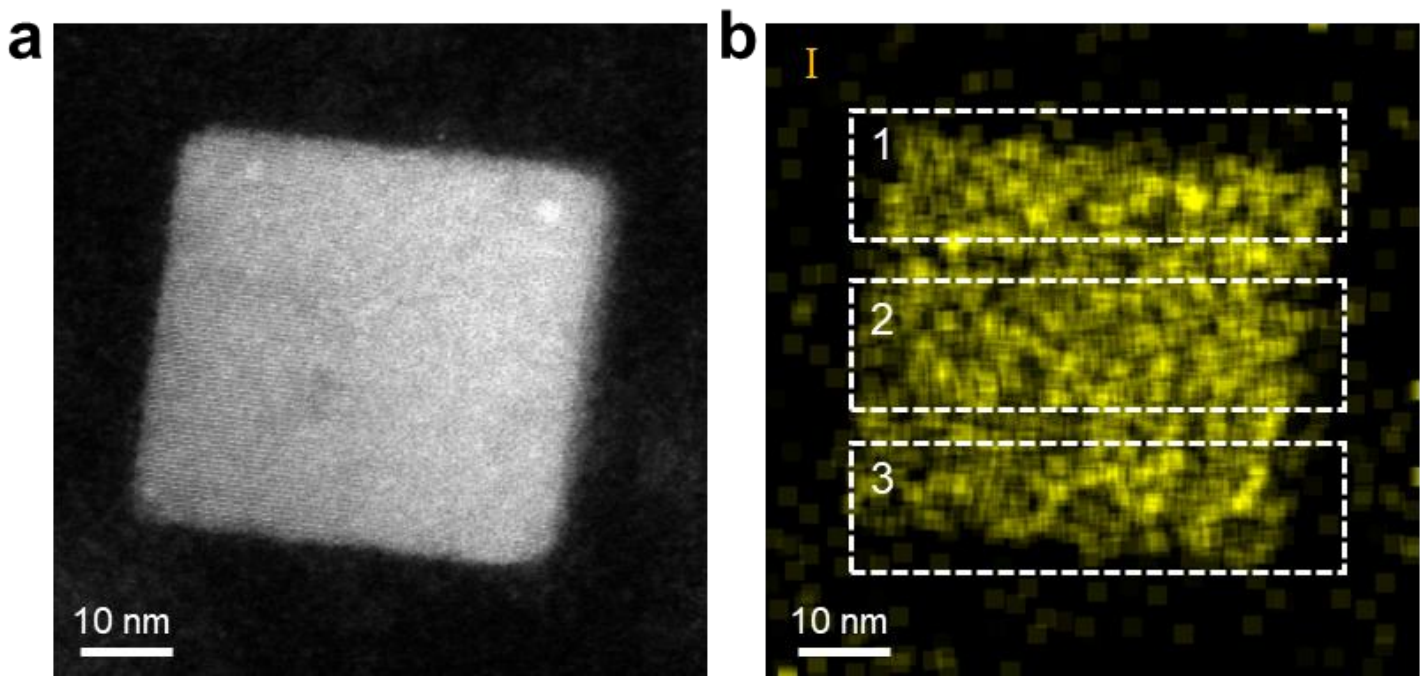

c

	I/Pb
Region 1	3.06
Region 2	3.05
Region 3	2.96

Supplementary Figure 6 | Surface ion migration on a $CsPbI_3$ nanosheet. (a) HAADF-STEM image of a $CsPbI_3$ nanosheet. **(b)** The corresponding EDS mapping of I element. **(c)** Table showing the atomic ratio of I : Pb ≈ 3 : 1 within 3 white dashed boxes in b.

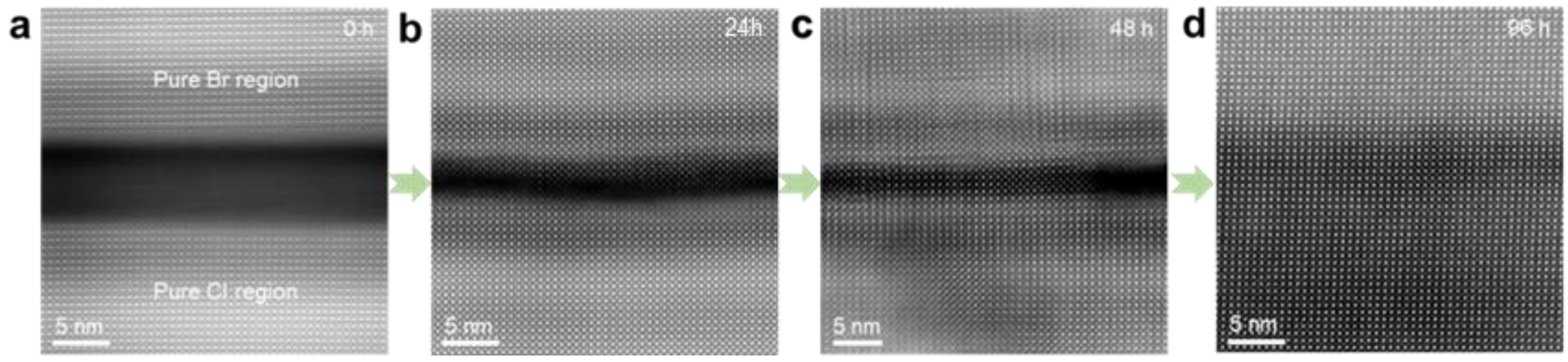

Supplementary Figure 7 | Atomic-scale observation of ion migration at the heterointerface with twist angle of 0°. (**a-d**) HAADF images presenting the connection process at the heterointerface stored in vacuum for different time.

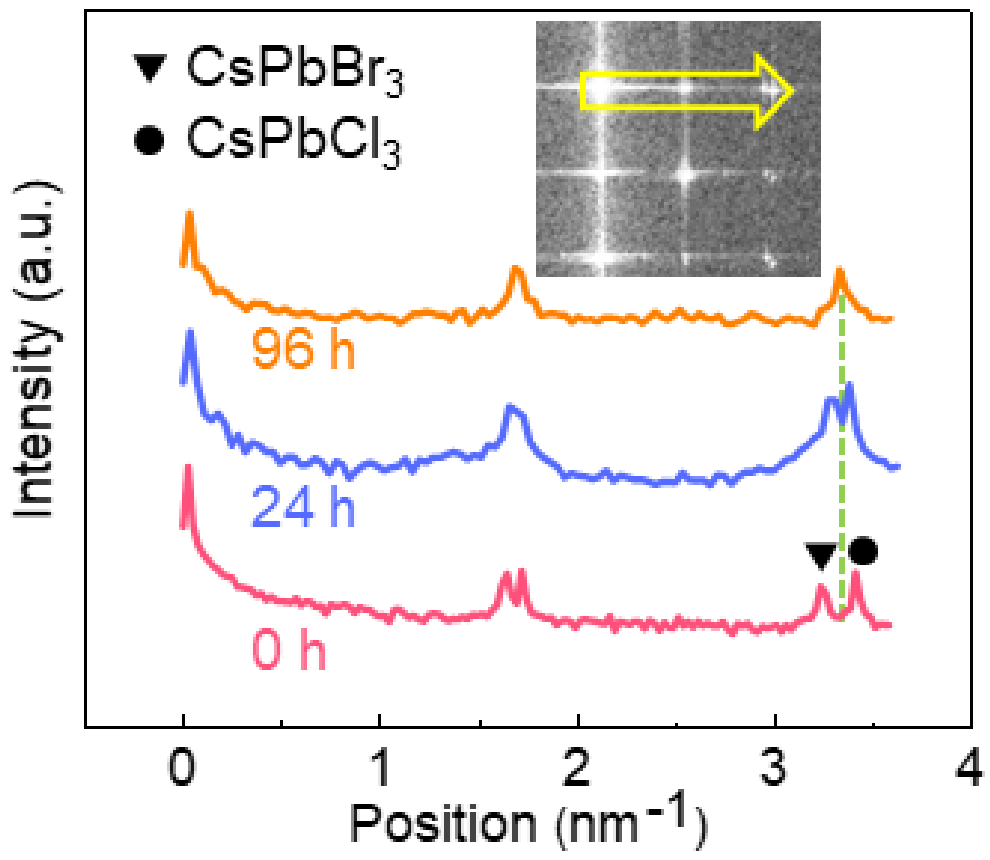

Supplementary Figure 8 | Intensity profiles of FFT spots along the arrow in the inset for the 0° heterointerface at 0, 24 and 96 hours. Inset is the FFT pattern acquired from Fig. S7.

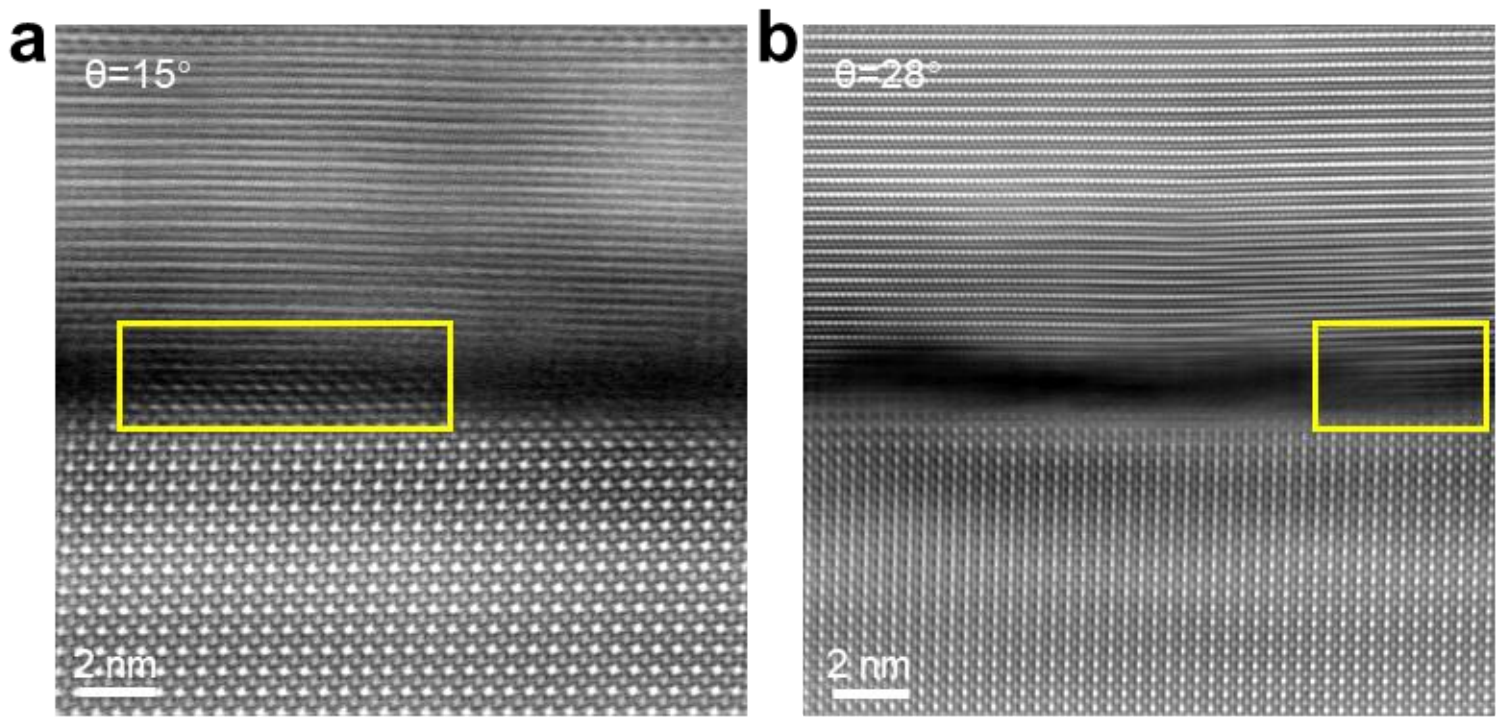

Supplementary Figure 9 | High-magnification HAADF-STEM images of the areas indicated by yellow boxes in Fig. 2. (a) 15° twist; **(b)** 28° twist.

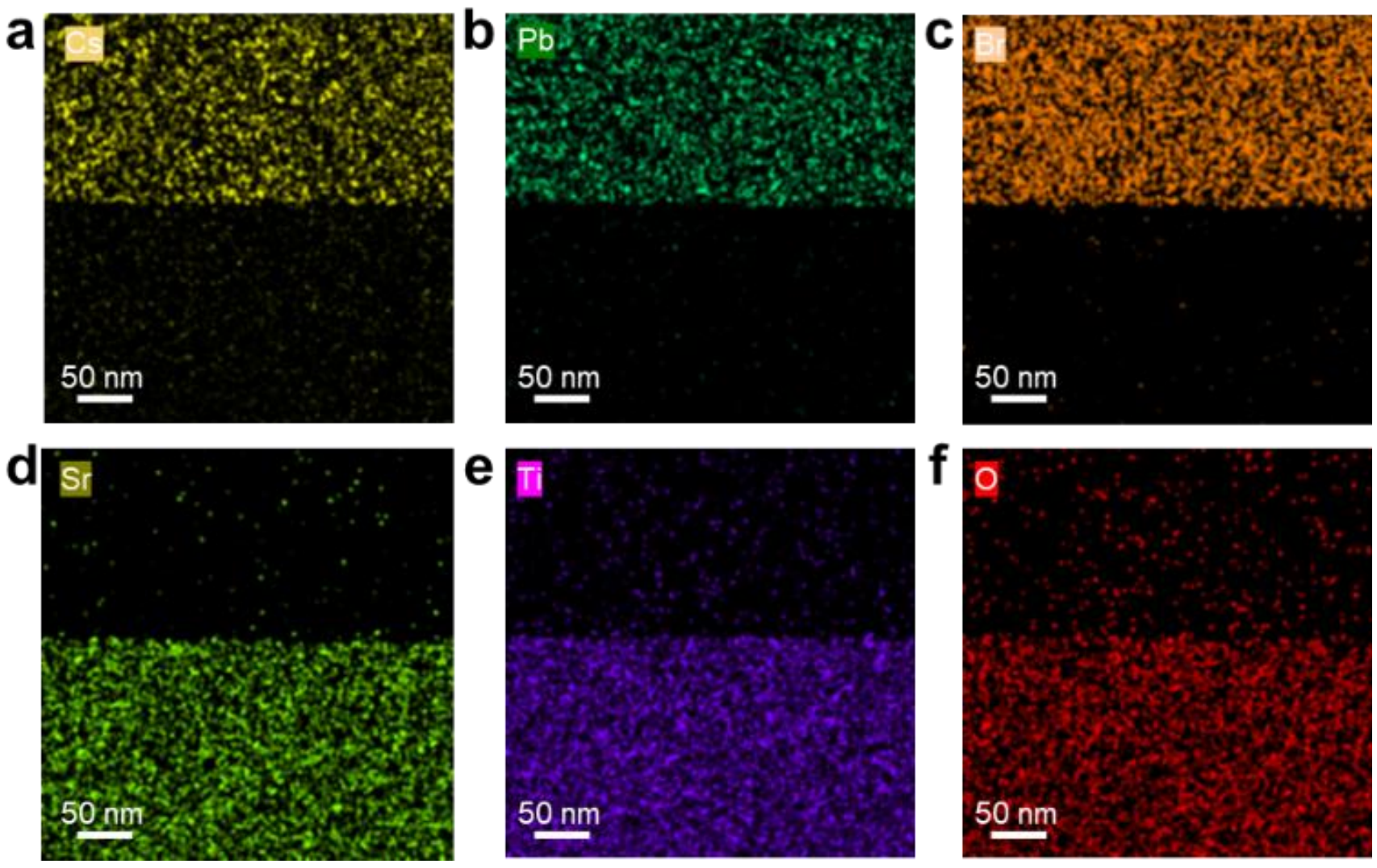

Supplementary Figure 10 | EDS elemental maps at the $CsPbBr_3/SrTiO_3$ heterointerface after storing in vacuum for 96 h. (a) Cs, **(b)** Pb, **(c)** Br, **(d)** Sr, **(e)** Ti, and **(f)** O.

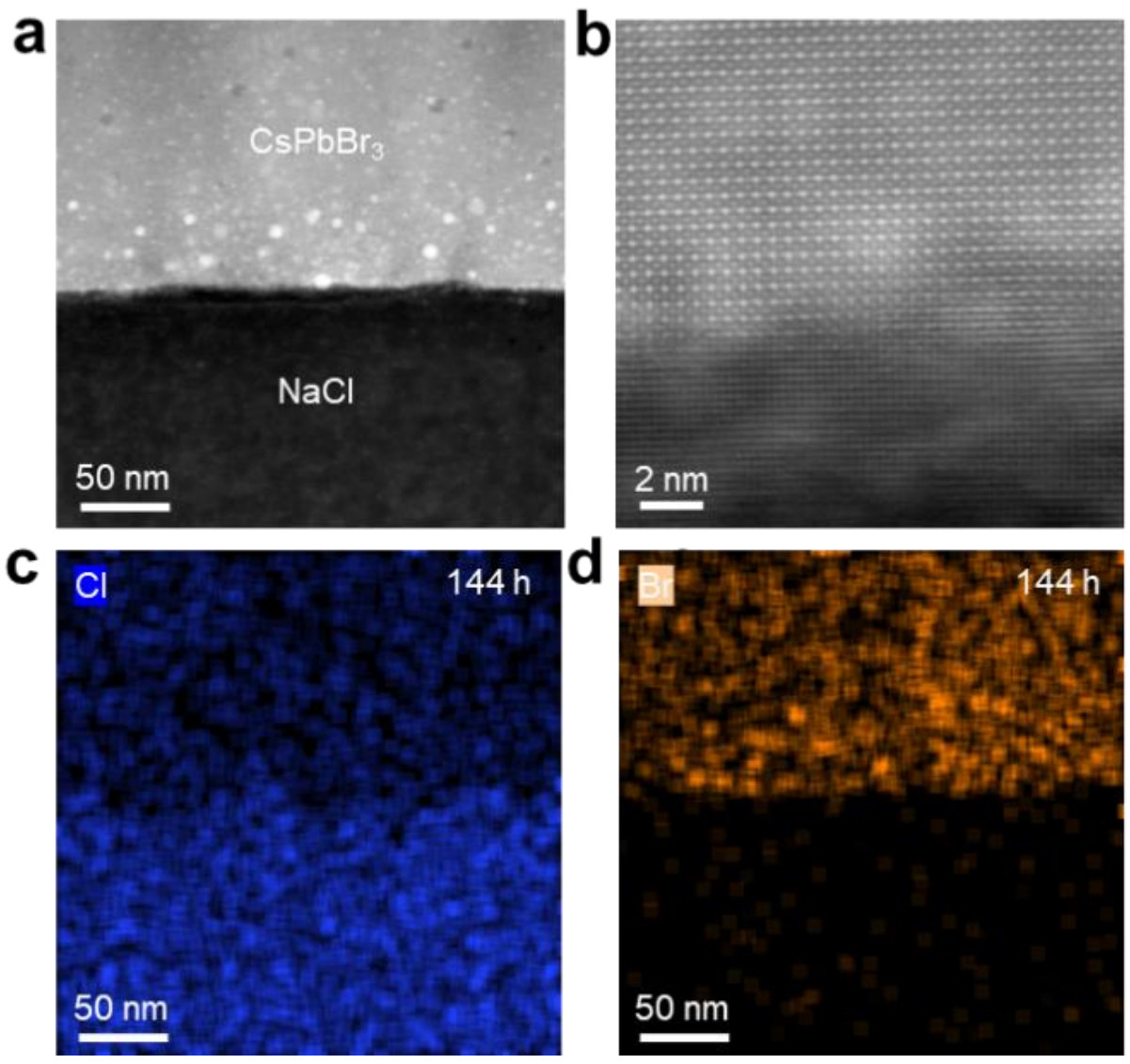

Supplementary Figure 11 | Halide ion migration behavior at the $CsPbBr_3$/NaCl heterostructure. **(a)** HAADF image of the heterostructure. **(b)** Atomic-resolution HAADF-STEM image of the heterointerface. **(c, d)** The corresponding EDS mapping of Cl and Br of the heterostructure after storing in vacuum for144 hours.

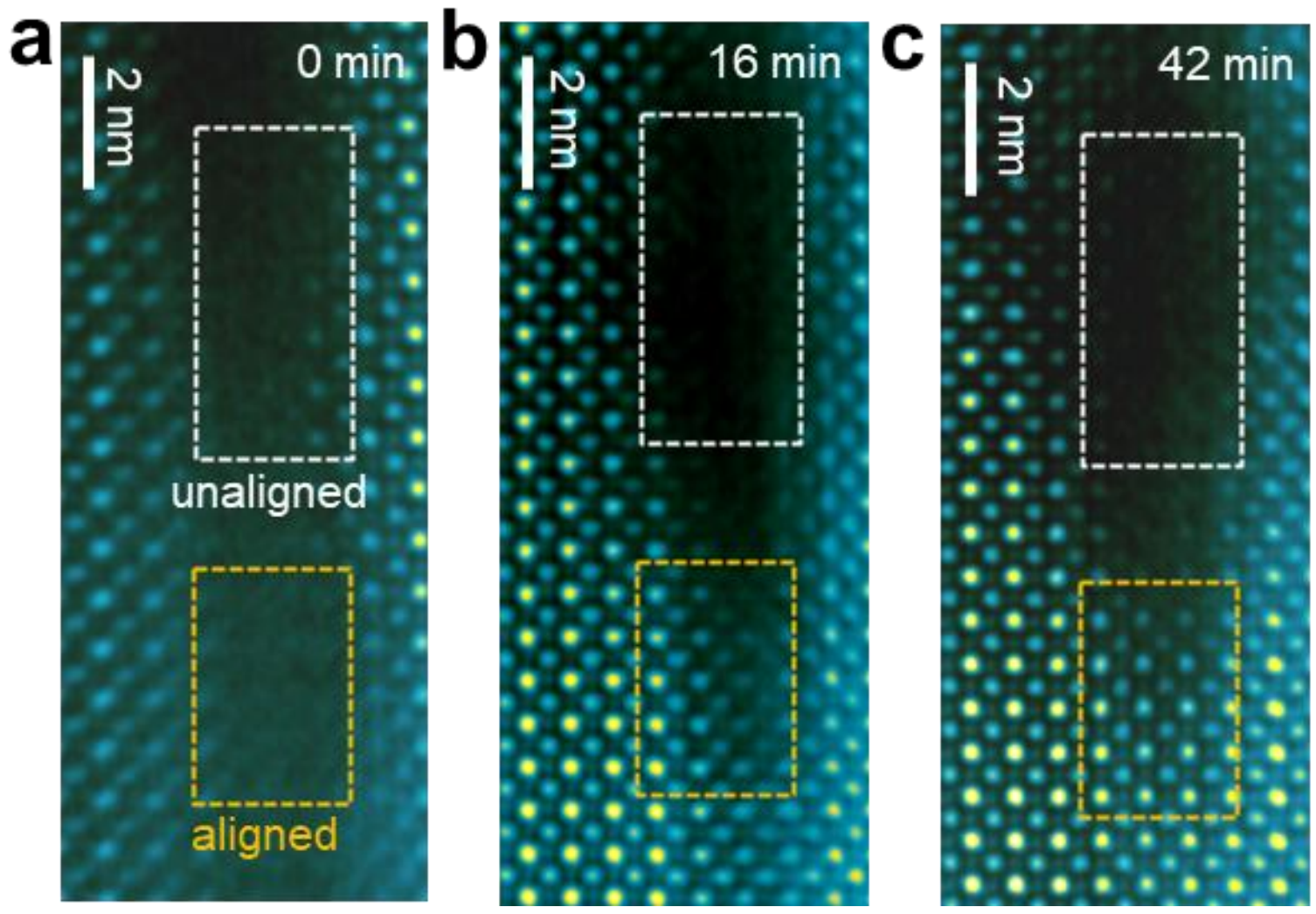

Supplementary Figure 12 | Time-series in-situ HAADF images of the 0° heterointerface under electron beam illumination. The white and orange dashed rectangular indicate unaligned and aligned lattice, respectively. **(a)** 0, **(b)** 16, and **(c)** 42 min.

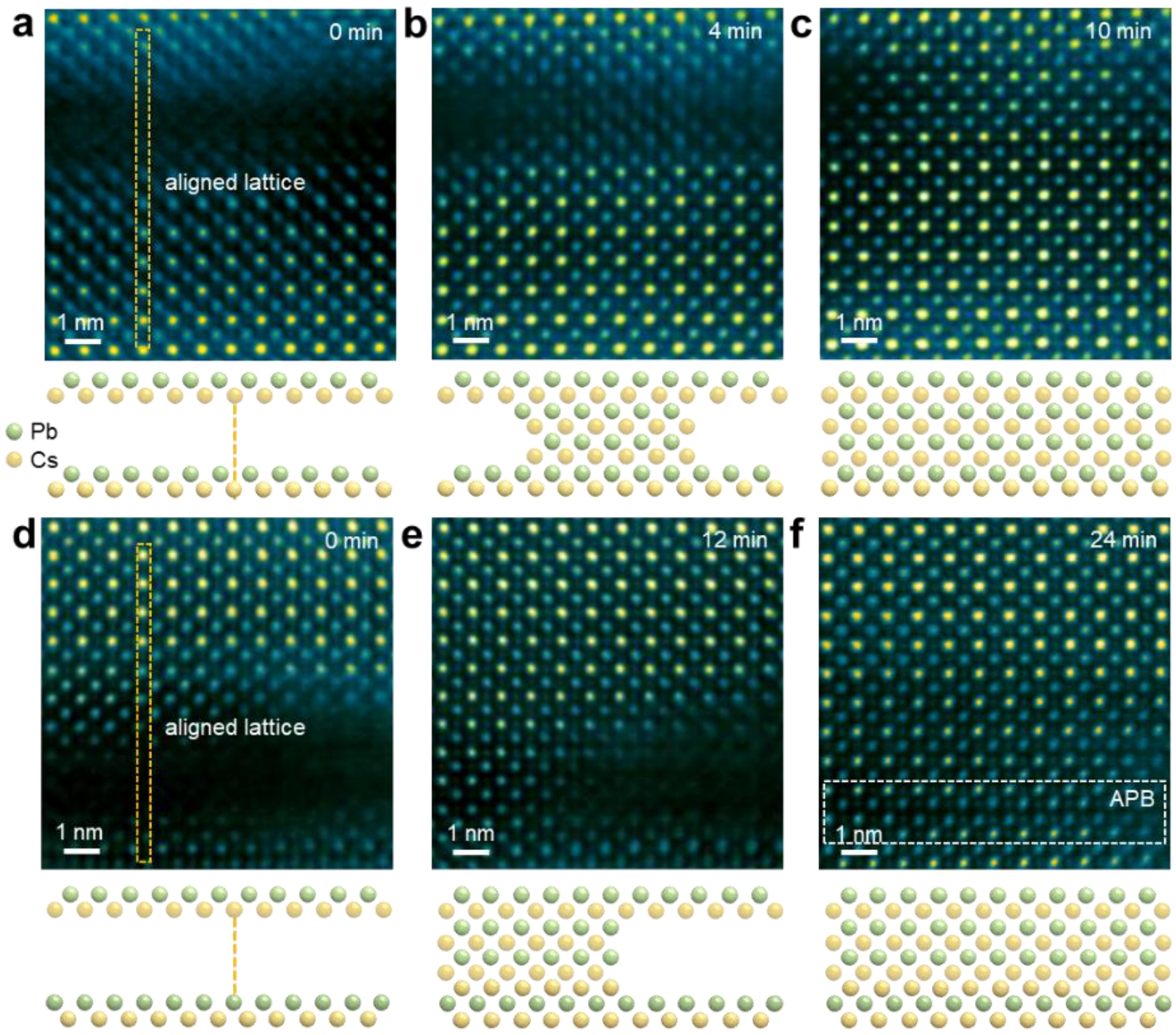

Supplementary Figure 13 | Atomic-scale observation of preferential connection for the 0° heterointerface with aligned lattices. (**a-c**) Time-series in-situ HAADF images of heterointerface under electron beam illumination wherein the Pb lattice in $CsPbBr_3$ aligns to that in $CsPbCl_3$. The bottom atomic models further depict the atomic-scale diffusion pathway. The orange and green balls represent Cs and Pb atom, respectively. (**d-f**) Time-series in-situ HAADF images of heterointerface wherein the Pb lattice in $CsPbBr_3$ offsets a half of unit to that of $CsPbCl_3$. The white dashed rectangular indicates the formation of anti-phase boundary.

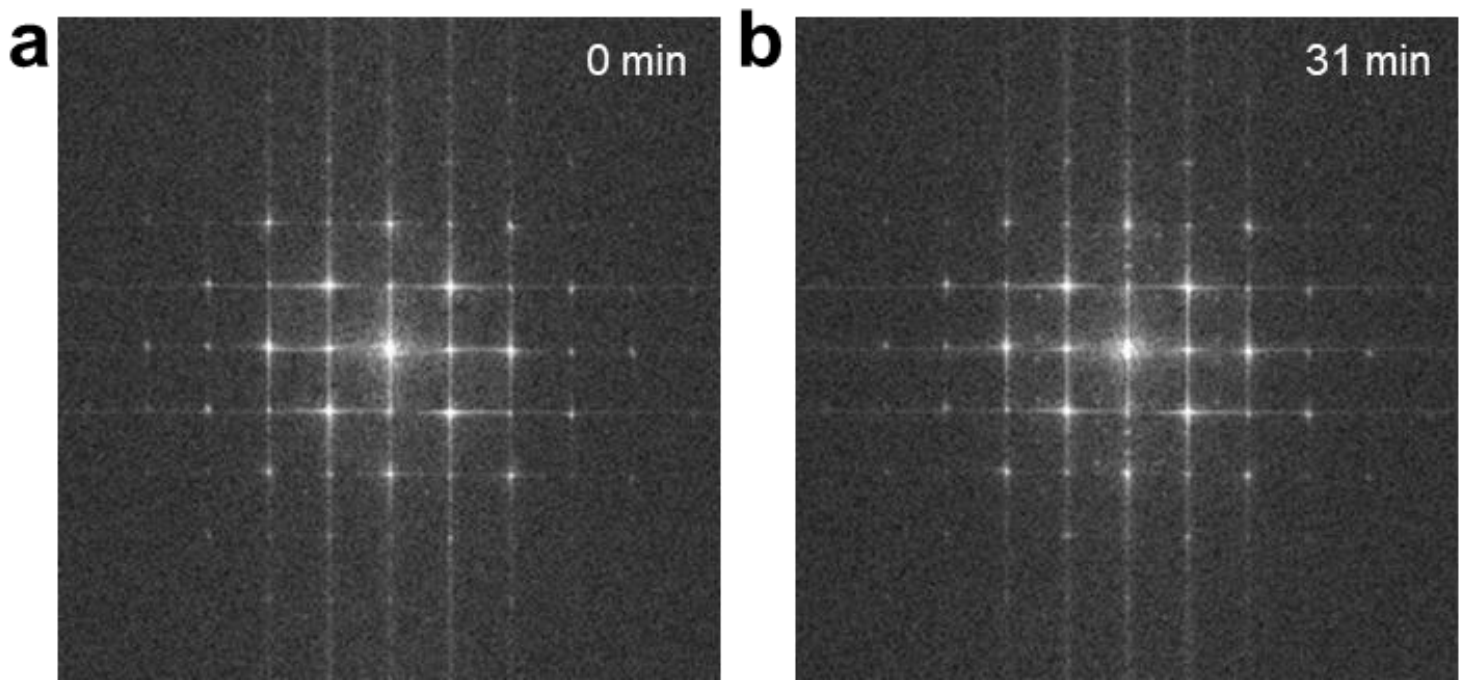

Supplementary Figure 14 | FFT patterns acquired from Fig. 3. (a) Fig. 3a, and **(b)** Fig. 3c.

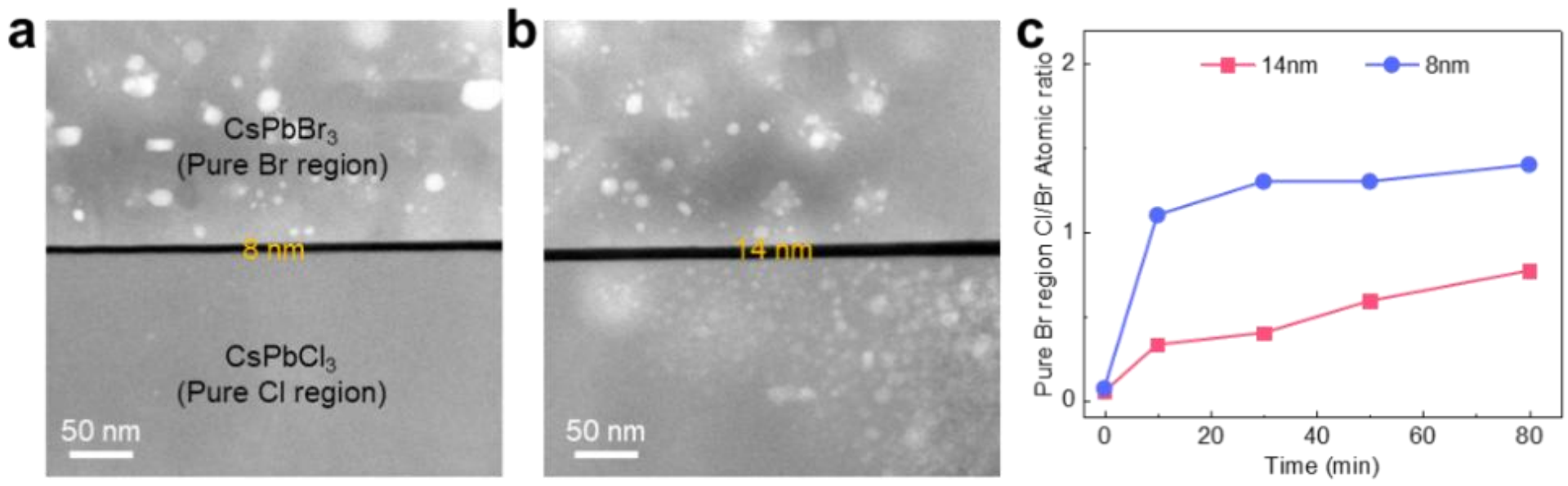

Supplementary Figure 15 | Effect of initial interfacial gap on halide interdiffusion for the 0° heterostructure. (a, b) HAADF-STEM image of the $CsPbBr_3$/$CsPbCl_3$ heterointerface with an initial gap width of (a) ~8 nm, (b) ~14 nm. **(c)** Time evolution of the Cl/Br atomic ratio from pure Br region under 150 °C heating.

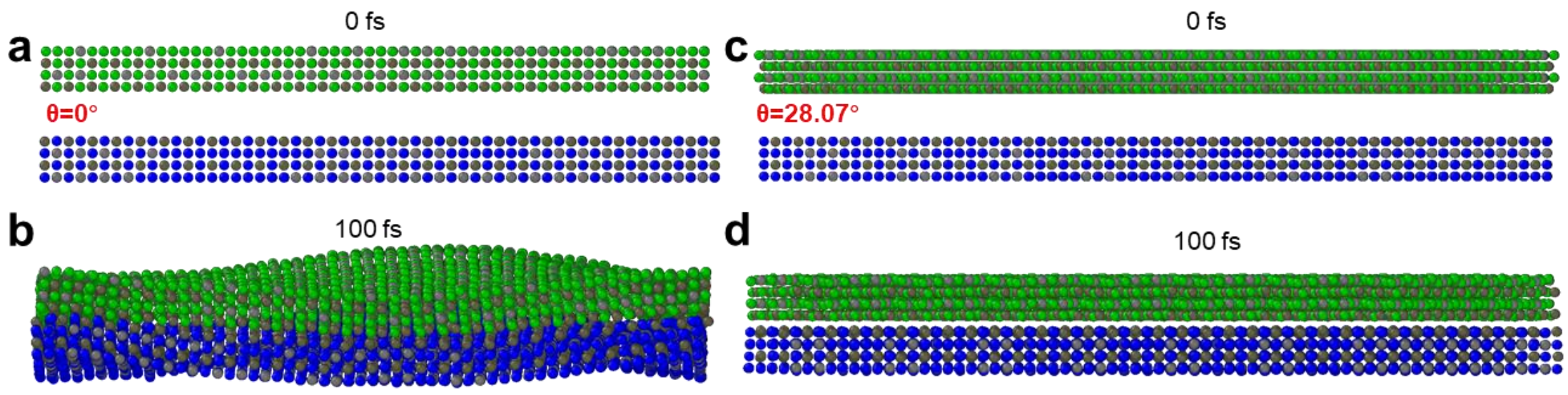

Supplementary Figure 16 | MD simulations of ion diffusion in $CsPbBr_3$/$CsPbCl_3$ heterostructures with Cs-X (X = halide) terminations. (a, b) 0° twisted heterostructure. **(c, d)** 28.07° twisted heterostructure.

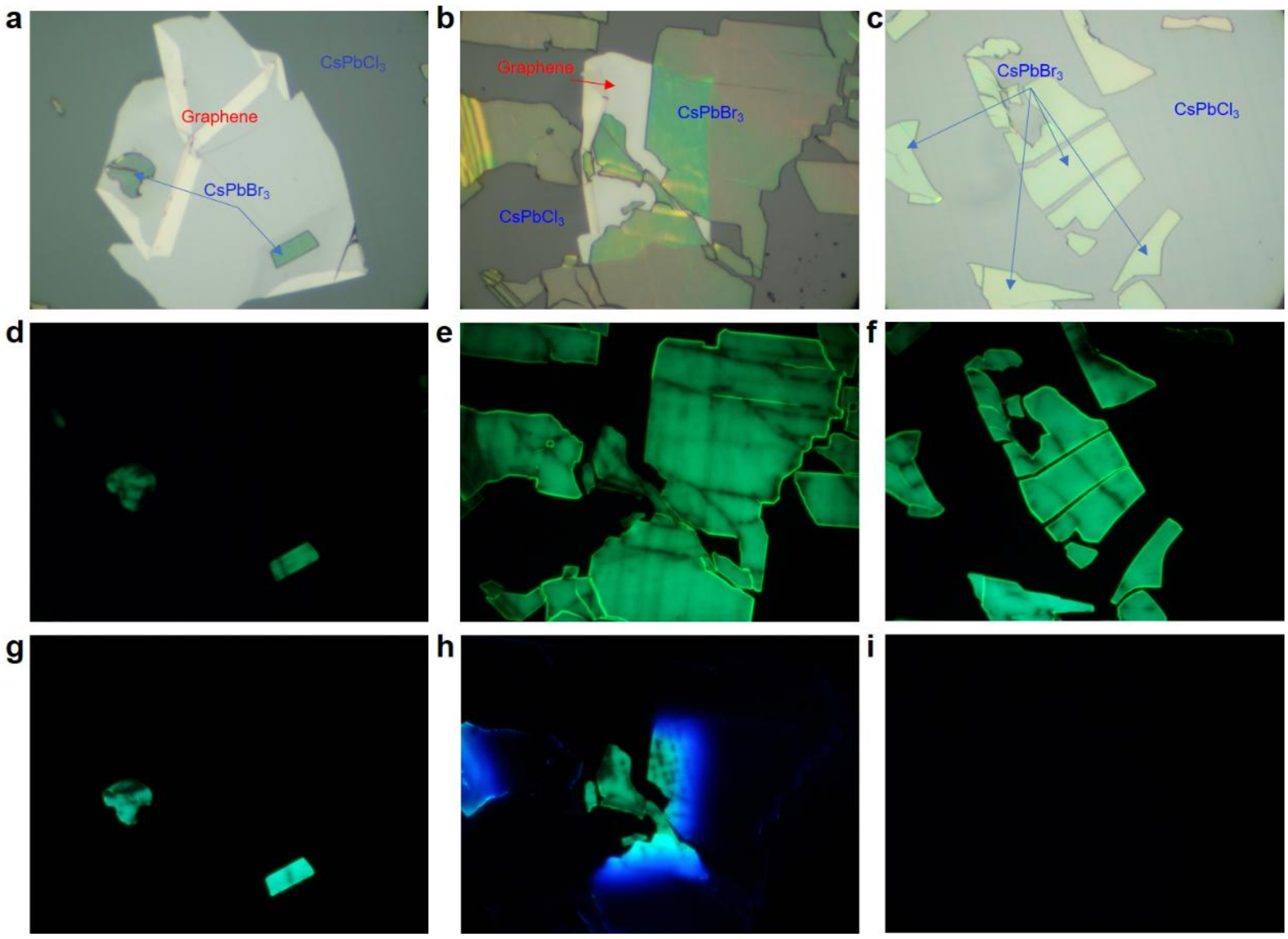

Supplementary Figure 17 | Graphene interlayer engineering suppresses halide ion migration in $CsPbBr_3$-$CsPbCl_3$ heterostructures. Optical images of $CsPbBr_3$-graphene-$CsPbCl_3$ heterostructure. (**a**) Full-blocking. (**b**) Partial-blocking. (**c**) No-blocking. (**d-f**) Initial confocal PL mapping images of the three heterostructures. (**g-i**) Confocal PL mapping images after thermal annealing at 150° C for 1 h of the three heterostructures. Images acquired with a 400 nm laser as the excitation wavelength with emission detection channel widths of 475-700 nm.

Supplementary Table 1 | The (m, n) integer pair and the corresponding twist angles.

m	n	θ (degree)
1	6	18.92
1	5	22.61
1	4	28.07
1	3	36.87
2	5	43.60
5	38	14.99

Note: These angles were selected because periodic boundary conditions require commensurate supercells for the twisted square lattice. The allowed twist angles can be described by $\theta = 2\arctan(m/n)$ where m and n are coprime integers. The twist system comprises $2(m^2 + n^2)$ unit cells. The (m, n) integer pair and the corresponding twist angles are presented in the following table.

For the experimental twist angle of ~15°, the closest commensurate model corresponds to (5,38), which contains 2938-unit cells and 14690 atoms. This system size is beyond our first-principles computational capability.

We have also clarified that, due to the fourfold symmetry of the cubic lattice, twist angles from 0° to 45° are sufficient to represent the distinct interfacial configurations. This 28.07° twist geometry maximizes atomic misalignment and decreases vdW coupling.

Supplementary Table 2 | The evolutions of the PL peak from the $CsPbBr_3$ side.

	0°	28°
0 min	529 nm	529 nm
10 min	527 nm	529 nm
20 min	526 nm	527 nm
40 min	525 nm	526 nm
60 min	525 nm	525 nm
80 min	524 nm	526 nm
100 min	524 nm	524 nm
120 min	523 nm	524 nm
150 min	522 nm	524 nm
170 min	520 nm	522 nm
190 min	516 nm	520 nm
220 min	510 nm	516 nm
250 min	507 nm	516 nm
