# Supplementary material for: Twistionics in halide perovskites
Source: Nat Commun. 2026 Aug 25;17:10177. doi: 10.1038/s41467-026-77126-7 (PMC13612815; doi:10.1038/s41467-026-77126-7)
Supplement: Supplementary file 2 [file 41467_2026_77126_MOESM2_ESM.pdf]

nature portfolio

Peer Review File

# Twistionics in halide perovskites

Corresponding Author: Professor Shulin Chen

**This file contains all reviewer reports in order by version, followed by all author rebuttals in order by version.**

**Attachments originally included by the reviewers as part of their assessment can be found at the end of this file.**

Version 1:

Reviewer comments:

Reviewer #1

(Remarks to the Author)
In this work the authors provide atomically resolved characterization results revealing how halide ions undergo cross-interface migration at the $CsPbBr_3/CsPbCl_3$ interface, and how interfacial twisting suppresses their cross-interface transport. Combined with theoretical modeling, this work elucidates the underlying mechanisms governing halide ion migration across the interface. The authors employ STEM-HAADF to achieve atomic-resolved characterization of halide ion migration in perovskites cross heterojunction interfaces. It provides an in-depth elucidation of the detailed migration mechanism and its entire evolution process. Based on these experimental observations, the authors propose a strategy to suppress ionic migration across perovskite heterojunctions by introducing interfacial twisting, and further apply this method in devices. These findings are of significant importance. For example, in 2D/3D heterojunction perovskite photovoltaic devices, the diffusion of organic cations from the 2D perovskite into the 3D perovskite lattice leads to performance degradation. This work therefore provides valuable guidance for the rational design of stable 2D/3D perovskite heterojunctions. Beside the experimental investigation, the study further integrates molecular dynamics simulations to demonstrate the energetic feasibility and physical rationality of the proposed interfacial ion-migration pathway, offering complementary theoretical support for the experimental conclusions. Overall, this is an excellent work, and I recommend acceptance of this work after the authors address the following points.
1. Further clarification is needed on the universality, and relevant experimental data need to be supplemented to explain whether the ion cross-interface-migration mode mentioned in this study generally exists at any lattice-match interface or only exists at the interface of perovskite-perovskite heterojunctions.
2. There are issues with the consistency of the research content in the article, and there are not good control conditions when comparing the results, resulting in certain loopholes in the experimental conclusions, such as:
(1) In Figures 1d and 5g, the article only provides PL images under heating conditions of 150 degree C, which differs from the STEM characterization conditions in the article. The authors should provide PL images at the same temperature as during the characterization process.
(2) In Figures 3a, d, and g, there are significant differences in the flatness of the sample interface, with Figure 3a being noticeably rougher. This may lead to differences in the gap between the sample in Figure 3a and Figures 3d and g. If possible, please choose an interface of sufficient flatness and control the interface spacing to further enhance the rigor of the characterization results.
(3) In Figure 4, the model constructed by simulation calculation has different terminations at the interface, but the same termination at the interface in the schematic diagram of Supplementary Figure 7. Please provide a brief explanation and make modifications to ensure consistency in the article regarding whether chemical terminations will affect the migration of halide ions.
3. Some hypotheses proposed in the article lack sufficient support:
(1) The article suggests that van der Waals forces are responsible for this result, but considering the ionic properties of perovskite crystal, it also exhibits strong electrostatic forces at the surface. Can the authors comment how to eliminate the influence of electrostatic forces?
(2) Since the article suggests that lattice constant matching can enhance van der Waals forces, have the authors tried to design experiments to bond perovskite materials with halide crystals that match their lattice constants and observe whether similar halide ion migration behavior occurs?
(3) The article mentions that the migration of halide ions at the heterojunction interfaces requires the formation of specific ion transport channels, as shown in Figures 1a and 1b. However, it does not provide clear characterization results to explain whether halide ions also undergo cross interface migration before channel formation, whether halide ion migration requires

the existence of interface channels, and whether further experimental data can be supplemented to explain this.

Reviewer #2

(Remarks to the Author)
Review „Twistionics in halide perovskites" by Sanxia Yin et. al.

In this work, the authors investigate ion migration across a halide perovskite heterostructure with and without twist angle. Ion migration at and across the interface of twisted heterostructure is examined in detail on the microscopic level using low-dose TEM. The authors employ molecular dynamics (MD) simulations to corroborate the experimental findings. For the macroscopic scale, complementary transport data (IVCs) are presented.

The manuscript provides valuable data that offer insights into the mechanisms of ion migration. The authors' most important finding is that ion migration is dependent on the twist angle, with a minimum at 28°.

The experimental results are of high quality and provide new and important insights into ion migration at twist boundaries of halide perovskites, an emerging field of research. Research topic and scientific results of the manuscript appeal to a sufficiently large readership.

In principle, the manuscript would be suitable for publication in Nature Communications, however, in its current form it does not quite meet the expected standards, and I therefore propose the following major modifications.

For further details, please see attached pdf.

Reviewer #3

(Remarks to the Author)
This is an interesting study that looks at inter-diffusion of ions from a block of $CsPbBr_3$ to a block of $CsPbCl_3$ perovskite as a function of the twist angle between the blocks. In this study two crystals are separated by a 4 nm gap and the authors carry out an elegant imaging study showing the formation of ion bridges and ultimately the melding of the two interfaces. It makes sense that introducing a twist in the lattice at the interface makes it harder for ions to pass across.

The work is interesting and I think publishable in subject to some revisions:
It is stated that ion migration is 'a fundamental instability' in halide perovskites, and that ion migration in PV leads to 'reduced open circuit voltage'. The argument lacks nuance! Ion migration can be bad for halide perovskites, although there are some cases when it can improve design tolerances and in PV ions tend to increase voltage and decrease current so it is not correct to say that they just straightforwardly reduce open circuit voltage and in fact the picture is complex (see e.g. Hart et al Energy Environ. Sci., 2024,17, 7107-7118; M. Córdoba and K. Taretto, Solar RRL, 2023, 8 , 2300742 and a nuanced discussion in ACS Energy Lett. 2025, 10, 10, 4849–4855)

Throughout the paper it is discussed that ion migration is strongly supressed in twisted systems. I think the authors should be more precise with their language here – ion migration can and will still occur within both the $CsPbBr_3$ and $CsPbCl_3$, either under illumination or applied voltage. Ions will still move to the interfaces in the devices described in figure 5, presumably with some initial ion accumulation at the perovskite-perovskite interface depending on the built in potential difference across that interface. This doesn't change much in the twisted systems, the difference is that it is then harder for ions to pass from the bromide perovskite to the chloride one (and/or vice versa). What is being controlled is that exchange of halide from on material to another, there will still be ion migration in each parent material.

I struggled a lot to see the images in Figure 2 that show 'diffusion channels' I think they need to be larger/zoomed in. By eye the channels are not clearly visible in the PDF. I'm also interested in the mechanism – e.g. the step 1 formation of 'interfacial diffusion channels' followed by lateral growth. The interface evolution is shown in wonderful detail. What I missed however is a discussion of the relevance to devices where there is not a 4 nm gap. Will the mechanism be the same? I also missed a discussion on the relative changes in diffusion rate for the twisted interfaces, compared to the many papers where diffusion is interrupted e.g. by a 2D layer as the interface or slowed down by e.g. cation substitution.

Finally I missed a discussion on how twistionics could be routinely and reproducibly introduced to devices? The paper fully lacks an experimental and methodology section, this needs to be added in full before publication. In addition it would be good to add more on how the electron beam is influencing the growth of interfacial structures, and how the rate of interdiffusion under imaging conditions compares to that under operating conditions for a device.

The van der waals interactions mentioned are interesting, the paper would benefit from more discussion on this.

Version 2:

Reviewer comments:

Reviewer #1

(Remarks to the Author)
The revised version has addressed all my concerns in my original report. I support publication of this paper as is.

Reviewer #2

(Remarks to the Author)
In their response letter and the revised manuscript, the authors addressed all points of criticism raised. As a result, the manuscript has improved significantly.

I have no further comments and recommend publication in Nature Communications without further changes.

Reviewer #3

(Remarks to the Author)
I have read the revised manuscript and the detailed response to the reviewers' comments. The manuscript has been substantially improved and I am happy to recommend it for publication.

**Open Access** This Peer Review File is licensed under a Creative Commons Attribution 4.0 International License, which permits use, sharing, adaptation, distribution and reproduction in any medium or format, as long as you give appropriate credit to the original author(s) and the source, provide a link to the Creative Commons license, and indicate if changes were made.
In cases where reviewers are anonymous, credit should be given to 'Anonymous Referee' and the source.
The images or other third party material in this Peer Review File are included in the article’s Creative Commons license, unless indicated otherwise in a credit line to the material. If material is not included in the article’s Creative Commons license and your intended use is not permitted by statutory regulation or exceeds the permitted use, you will need to obtain permission directly from the copyright holder.
To view a copy of this license, visit https://creativecommons.org/licenses/by/4.0/

## Response to Referees' Comments

We would like to thank the reviewers for their constructive comments and helpful suggestions concerning our manuscript. In response, we have conducted additional experiments and analyses, revised the manuscript accordingly, and clarified the relevant points throughout the text. Below is our point-by-point response to the reviewers. We have included a copy of the manuscript that highlights the changes made in response to the reviewers' comments.

Reviewer #1 (Remarks to the Author):

In this work the authors provide atomically resolved characterization results revealing how halide ions undergo cross-interface migration at the $CsPbBr_3/CsPbCl_3$ interface, and how interfacial twisting suppresses their cross-interface transport. Combined with theoretical modeling, this work elucidates the underlying mechanisms governing halide ion migration across the interface. The authors employ STEM-HAADF to achieve atomic-resolved characterization of halide ion migration in perovskites cross heterojunction interfaces. It provides an in-depth elucidation of the detailed migration mechanism and its entire evolution process. Based on these experimental observations, the authors propose a strategy to suppress ionic migration across perovskite heterojunctions by introducing interfacial twisting, and further apply this method in devices. These findings are of significant importance. For example, in 2D/3D heterojunction perovskite photovoltaic devices, the diffusion of organic cations from the 2D perovskite into the 3D perovskite lattice leads to performance degradation. This work therefore provides valuable guidance for the rational design of stable 2D/3D perovskite heterojunctions. Beside the experimental investigation, the study further integrates molecular dynamics simulations to demonstrate the energetic feasibility and physical rationality of the proposed interfacial ion-migration pathway, offering complementary theoretical support for the experimental conclusions. Overall, this is an excellent work, and I recommend acceptance of this work after the authors address the following points.

**Reply**: We are grateful for the reviewer's positive comments.

1. Further clarification is needed on the universality, and relevant experimental data need to be supplemented to explain whether the ion cross-interface-migration mode mentioned in this study generally exists at any lattice-match interface or only exists at

the interface of perovskite-perovskite heterojunctions.

**Reply**: We thank the reviewer for this important comment. To clarify the generality of the proposed cross-interface ion-migration mechanism, we performed additional control experiments using $CsPbBr_3$-$SrTiO_3$ (STO) and $CsPbBr_3$-NaCl heterostructures.

For the $CsPbBr_3$-STO heterostructure, although the lattice of $CsPbBr_3$ (583 pm) is approximately 1.5 times that of STO (390 pm), no obvious cross-interface anion migration was observed (Fig. S10). This is likely due to the rigid oxide lattice and distinct chemical bonding environment of STO[2], which prevent the formation of continuous halide-ion transport channels across the interface.

We further examined a $CsPbBr_3$-NaCl heterostructure, in which the lattice constant of NaCl (564 pm) is close to that of $CsPbCl_3$ (560 pm). In this case, $Cl^-$ migration toward the $CsPbBr_3$ side was observed, whereas $Br^-$ migration into the NaCl side was negligible (Fig. S11). This is mainly because the substitution of $Cl^-$ by $Br^-$ to form $NaCl_{1-x}Br_x$ solid solutions is thermodynamically unfavorable[5].

These results indicate that the ion cross-interface migration mechanism proposed in this work is mainly applicable to chemically and structurally compatible heterojunctions, rather than to any lattice-matched interface.

We have added this discussion on Line 19-24 Page 7 of the revised manuscript.

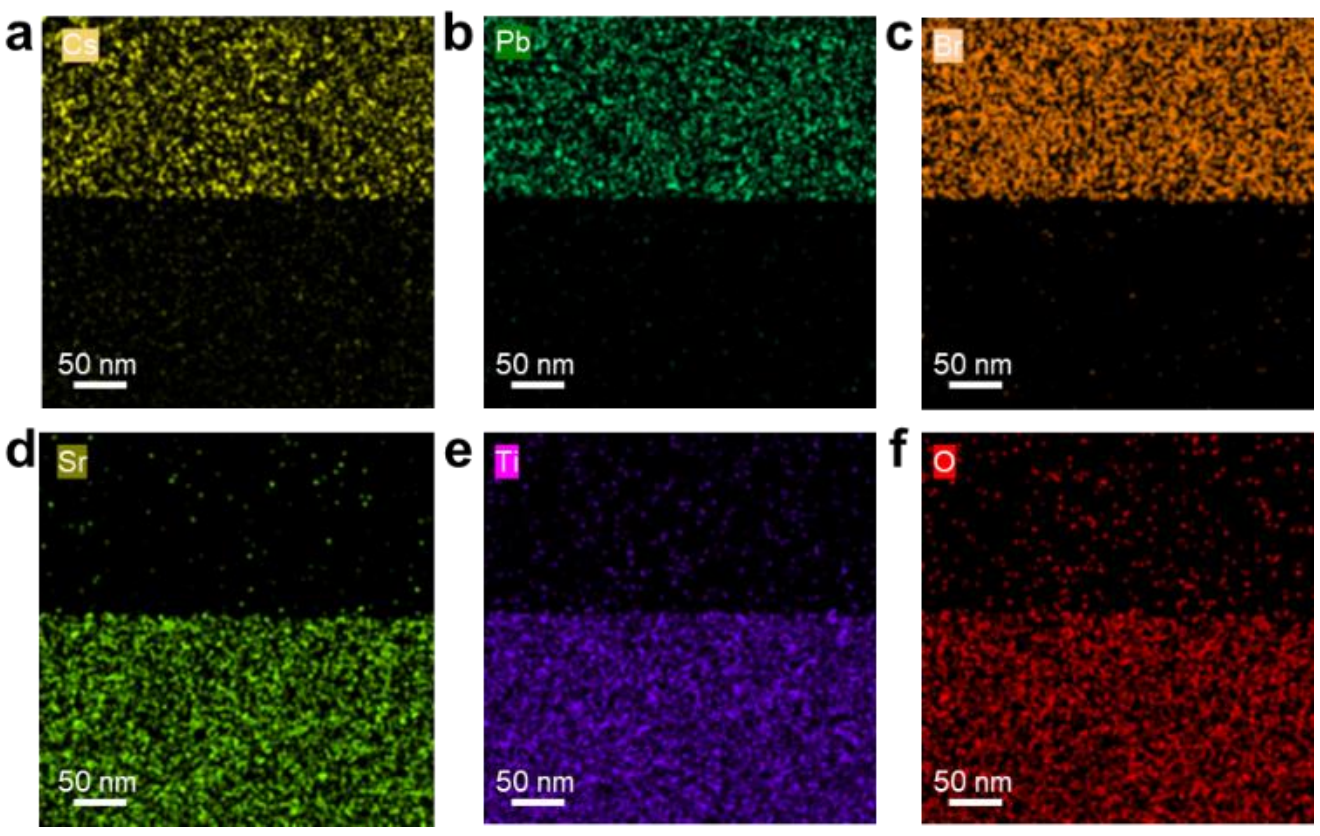


Supplementary Figure 10 | EDS elemental maps of Cs, Pb, Br, Sr, Ti, and O at the $CsPbBr_3/SrTiO_3$ heterointerface after storing in vacuum for 96 h.

2. There are issues with the consistency of the research content in the article, and there are not good control conditions when comparing the results, resulting in certain loopholes in the experimental conclusions, such as:

(1) In Figures 1d and 5g, the article only provides PL images under heating conditions of 150 degree C, which differs from the STEM characterization conditions in the article. The authors should provide PL images at the same temperature as during

the characterization process.

**Reply**: We thank the reviewer for this comment. We would like to clarify that the PL measurements were not intended to exactly reproduce the STEM experimental conditions, but to provide macroscopic optical validation of the twist-dependent ion-interdiffusion behavior observed by STEM.

In low-dose STEM, the electron beam can transfer energy to materials, driving ion migration and structural evolutions, similar to heat- or light-induced transitions[3]. In PL and device experiments, thermal annealing at 150 °C was used as an accelerated condition to obtain measurable halide interdiffusion within a practical time window. At room temperature, the interdiffusion process is much slower and does not readily produce clear PL changes within the same measurement duration.

We have added this discussion on Line 8-9 Page 8 and Line 2-3 Page 10 of the revised manuscript.

(2) In Figures 3a, d, and g, there are significant differences in the flatness of the sample interface, with Figure 3a being noticeably rougher. This may lead to differences in the gap between the sample in Figure 3a and Figures 3d and g. If possible, please choose an interface of sufficient flatness and control the interface spacing to further enhance the rigor of the characterization results.

**Reply**: We thank the reviewer for this careful observation. We agree that interfacial flatness and initial gap may affect the comparison of diffusion kinetics. To address this issue, we performed additional analyses.

First, Fig. S13 shows another 0° sample with a flatter interface, where diffusion channels still form rapidly after 10 min of electron-beam irradiation. This indicates that the fast channel formation in the 0° sample is not caused by interface roughness.

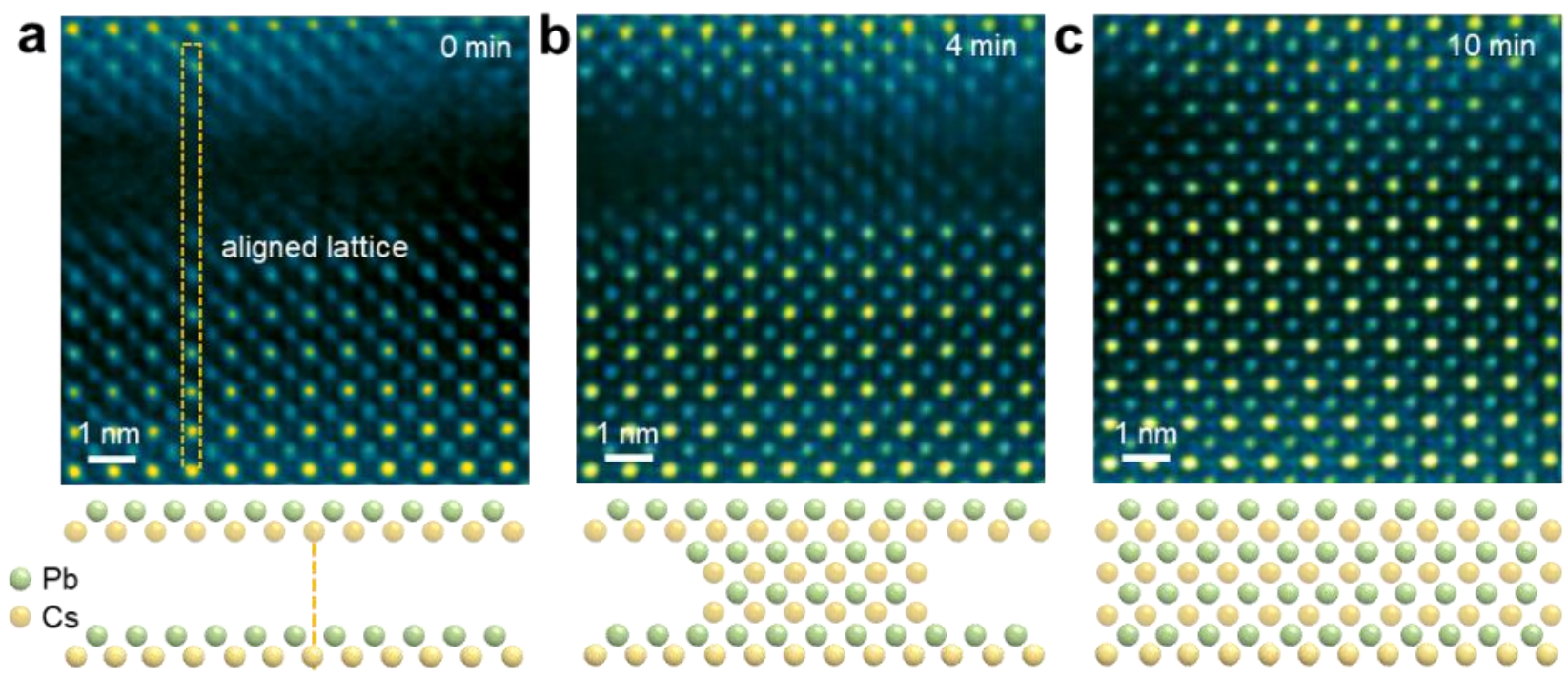


Supplementary Figure 13 | Atomic-scale observation of preferential connection for the 0° heterointerface with aligned lattices. (a-c) Time-series in-situ HAADF images of heterointerface under electron beam illumination wherein the Pb lattice in $CsPbBr_3$

aligns to that in $CsPbCl_3$. The bottom atomic models further depict the atomic-scale diffusion pathway. The orange and green balls represent Cs and Pb atom, respectively.

Second, we further analyzed the effect of initial interfacial gap on ion interdiffusion and found a wider gap delays interdiffusion under identical conditions (Fig. S15). However, in our experiments, the 0° sample has a wider initial gap (~4 nm) but undergoes complete interdiffusion more rapidly, whereas the 28° sample has a narrower gap (~2 nm) but shows the slowest channel formation and interdiffusion. Therefore, we conclude that the observed differences in diffusion kinetics in Fig. 3 arise primarily from the twist angle, rather than from minor variations in interfacial morphology.

We have added these discussions on Line 19-21 Page 8 of the revised manuscript.

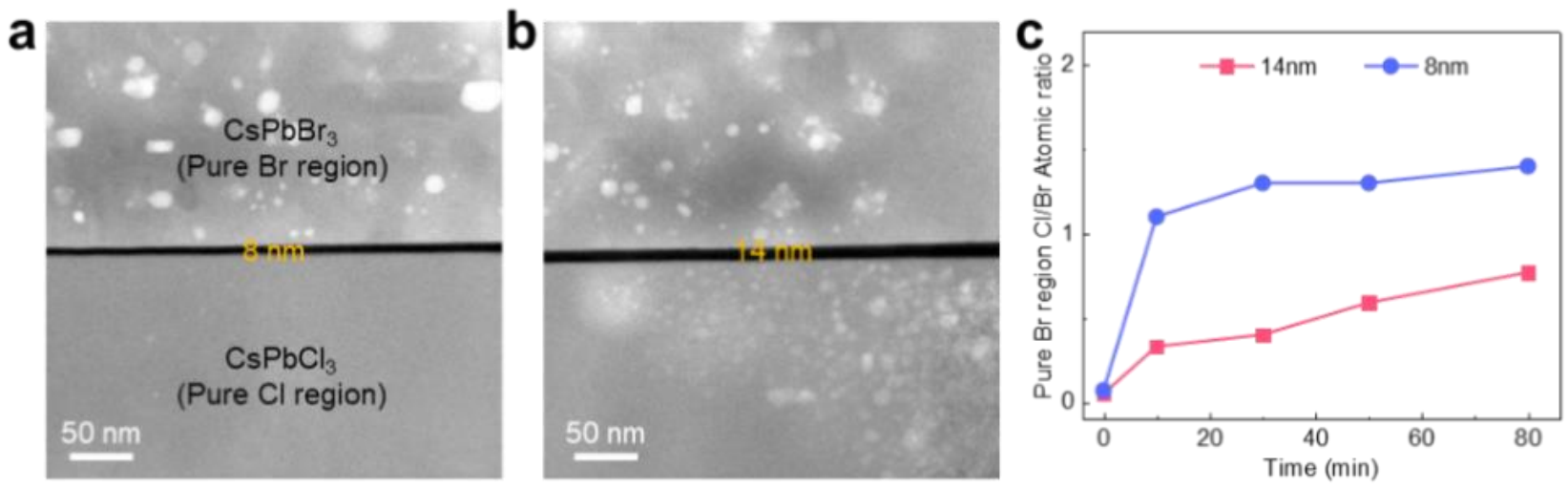


Supplementary Figure 15 | Effect of initial interfacial gap on halide interdiffusion. (a, b) HAADF-STEM image of the $CsPbBr_3/CsPbCl_3$ heterointerface with an initial gap width of (a) ~8 nm, (b) ~14 nm. (c) Time evolution of the Cl/Br atomic ratio at the two interfaces under 150 °C heating from pure Br region.

(3) In Figure 4, the model constructed by simulation calculation has different terminations at the interface, but the same termination at the interface in the schematic diagram of Supplementary Figure 7. Please provide a brief explanation and make modifications to ensure consistency in the article regarding whether chemical terminations will affect the migration of halide ions.

**Reply**: We thank the reviewer for pointing out this inconsistency. We have corrected the schematic in Fig. 7 (newly Fig. S13) so that the interfacial termination is consistent with the simulation model in Fig. 4.

In addition, we performed supplementary calculations using Cs-X (X = halide) terminations (Fig. S16). The results show that the 28.07° twisted interface still maintains a larger interfacial gap than the 0° interface after the same simulation time, indicating weakened interfacial coupling and suppressed cross-interface ion migration upon twisting. Therefore, chemical terminations might not affect the qualitative conclusion that twist angle suppresses ion migration.

We have added these discussions on Line 1-3 Page 9 of the revised manuscript.

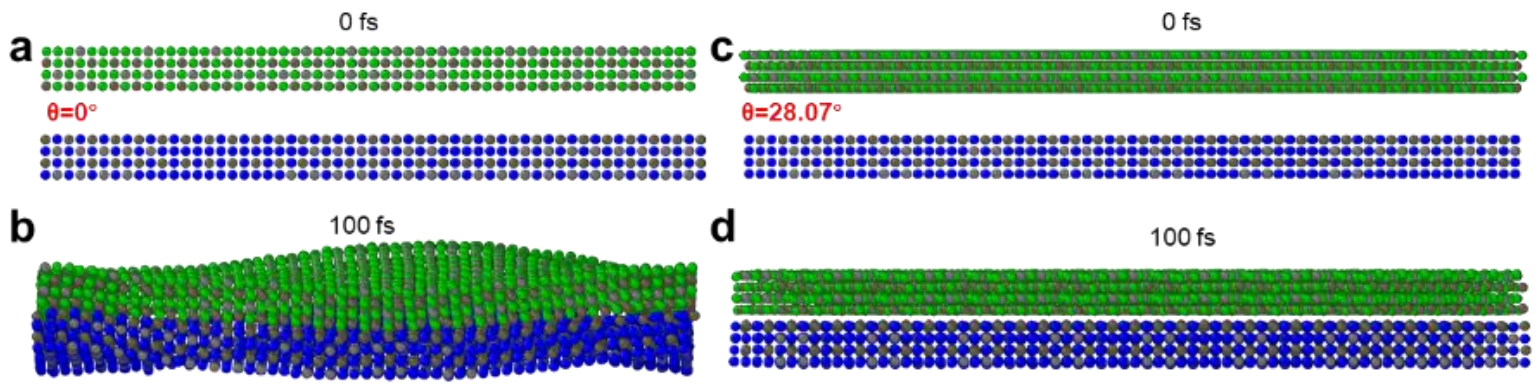


Supplementary Figure 16 | MD simulations of ion diffusion in $CsPbBr_3/CsPbCl_3$ heterostructures with Cs-X (X = halide) terminations. (a, b) 0° twisted heterostructure. (c, d) 28.07° twisted heterostructure.

3. Some hypotheses proposed in the article lack sufficient support:

(1) The article suggests that van der Waals forces are responsible for this result, but considering the ionic properties of perovskite crystal, it also exhibits strong electrostatic forces at the surface. Can the authors comment how to eliminate the influence of electrostatic forces?

**Reply**: We thank the reviewer for this comment. It should be clarified that van der Waals forces inherently include electrostatic interactions from a physical perspective and they act together and are not mutually exclusive mechanisms[4]. We have stated this in the revised manuscript.

(2) Since the article suggests that lattice constant matching can enhance van der Waals forces, have the authors tried to design experiments to bond perovskite materials with halide crystals that match their lattice constants and observe whether similar halide ion migration behavior occurs?

Reply: We thank the reviewer for this suggestion. Following this suggestion, we constructed a $CsPbBr_3$/NaCl heterostructure, considering that NaCl has a lattice constant of ~564 pm, close to that of $CsPbCl_3$ (~560 pm).

It is observed that $Cl^-$ migrate toward the $CsPbBr_3$ side, while $Br^-$ hardly migrate toward the NaCl side (Fig. S11). This is likely because the substitution of $Cl^-$ by $Br^-$ to form $NaCl_{1-x}Br_x$ solid solutions is thermodynamically unfavorable[5]. This result indicates that lattice matching can facilitate interfacial halide migration, but lattice matching alone is not sufficient to produce symmetric cross-interface ion interdiffusion. Chemical compatibility between the two lattices is also required.

We have added these experimental results and discussion to the revised manuscript on Page 7 Line 20-22.

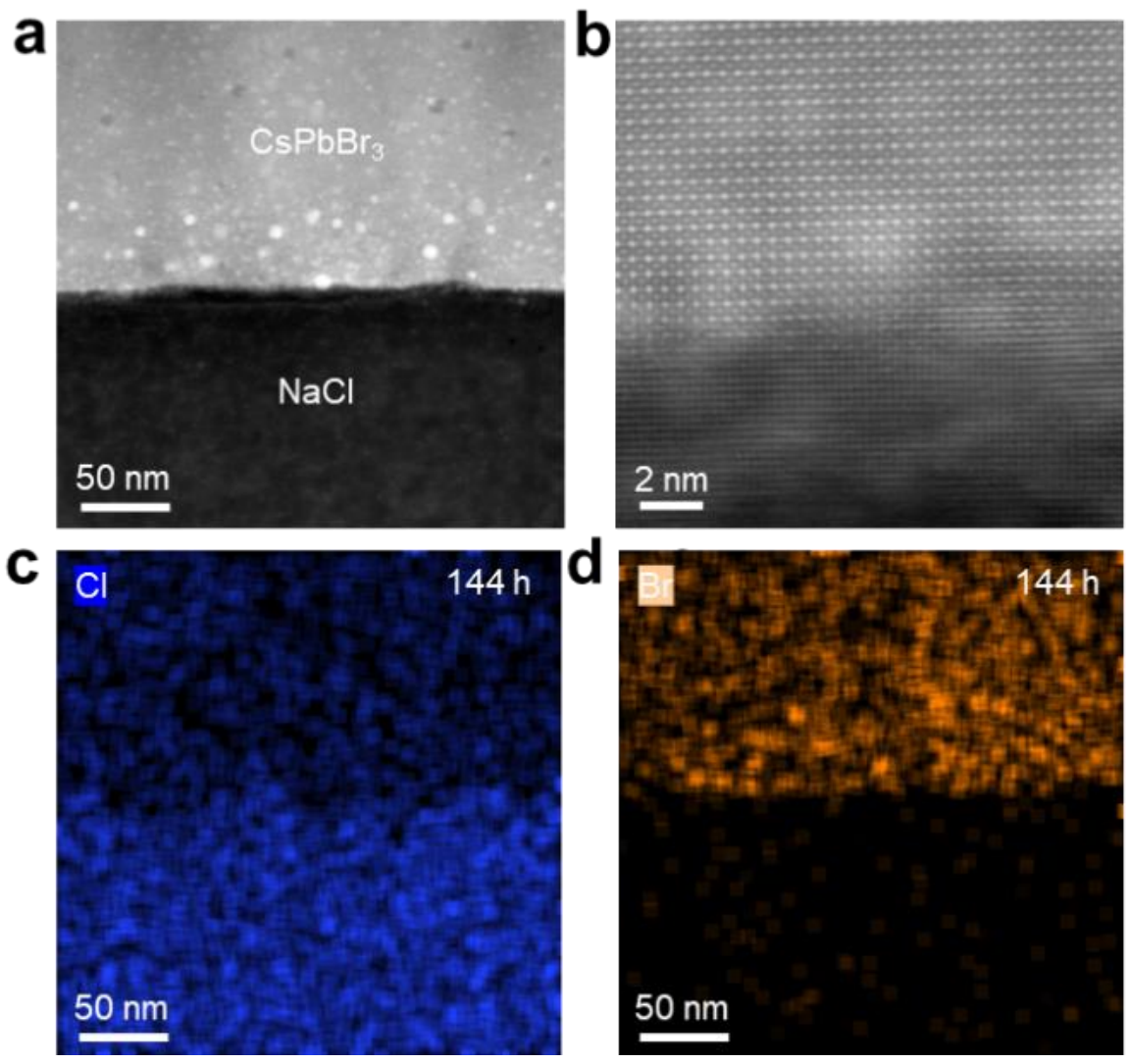


Supplementary Figure 11 | Halide ion migration behavior at the $CsPbBr_3$/NaCl heterostructure. (a) HAADF image of the heterostructure. (b) Atomic-resolution HAADF-STEM image of the heterointerface. (c, d) The corresponding EDS mapping of Cl and Br of the heterostructure after storing in vacuum for144 hours.

(3) The article mentions that the migration of halide ions at the heterojunction interfaces requires the formation of specific ion transport channels, as shown in Figures 1a and 1b. However, it does not provide clear characterization results to explain whether halide ions also undergo cross interface migration before channel formation, whether halide ion migration requires the existence of interface channels, and whether further experimental data can be supplemented to explain this.

Reply: We thank the reviewer for this comment. We believe the halide ion inter-migration requires the existence of interface channels. As shown in newly Fig. 2, obvious halide interdiffusion starts from the regions where interfacial channels have formed, whereas no clear cross-interface migration is observed in regions without such channels. This indicates that the channels provide the major pathway for halide-ion transport across the interface.

Before channel formation, ions diffuse toward and accumulate in the interfacial gap (Fig. S3). First-principles calculations show that the diffusion barrier from the bulk to the surface/gap (2.287 eV) is relatively lower than that directly crossing the gap (3.133 eV) or surface to bulk (2.574 eV) (Fig. S4). Consequently, halide ions first accumulate in the gap and gradually form perovskite bridges, and then undergo pronounced cross-interface interdiffusion through these channels.

We have added and highlighted these words on Line 6-14 and 18-22 on Page 6 of the revised manuscript

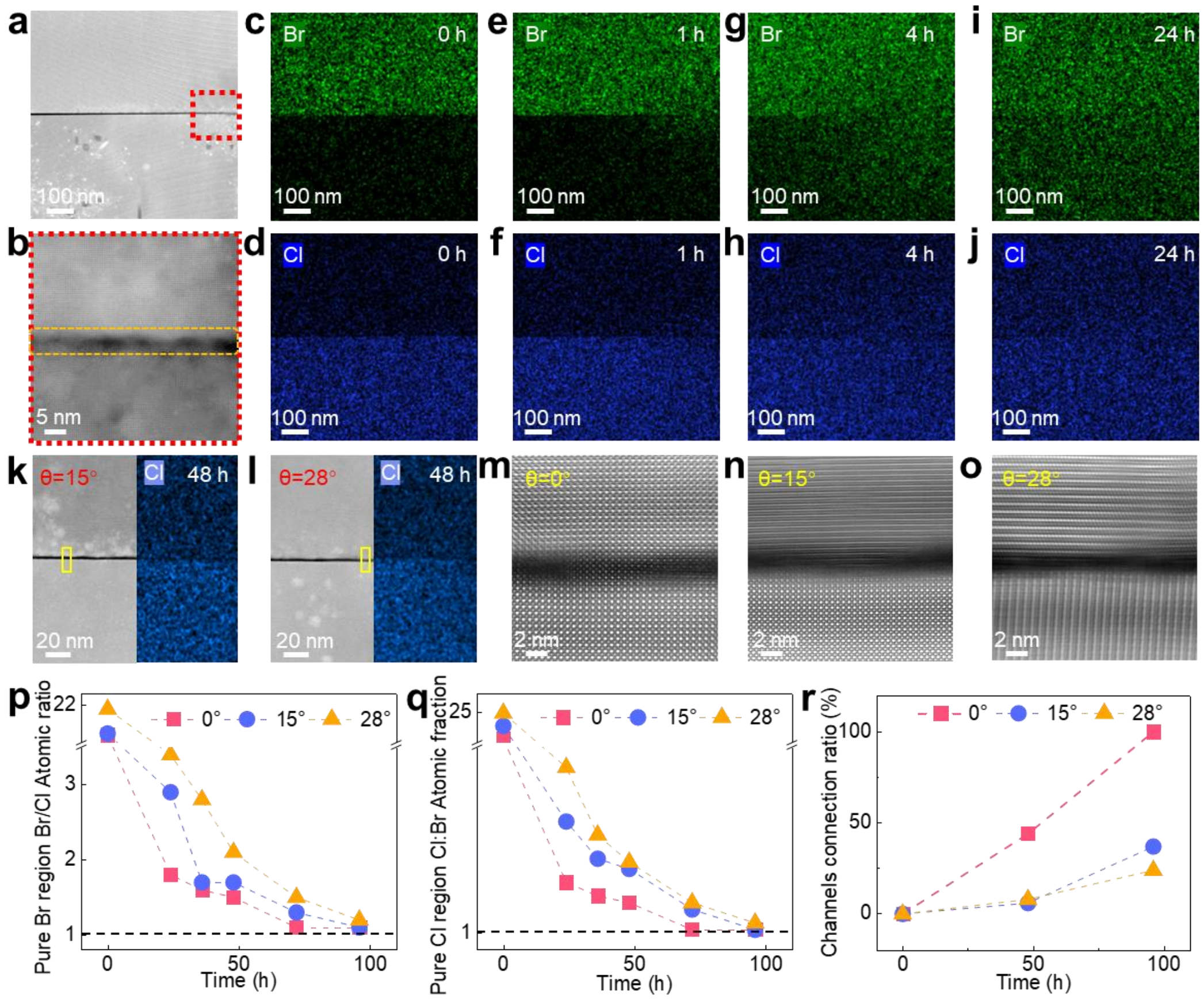


Fig. 1 | Twist-angle-dependent halide composition evolution and diffusion channels in $CsPbCl_3$–$CsPbBr_3$ heterostructures. (a, b) HAADF images of the diffusion channels in a twist angle of 0°. (c-j) The corresponding EDS mapping of Br and Cl at 0, 1, 4, and 24 hours. (k, l) HAADF images and corresponding EDS mapping of Cl of the heterostructures with twist angle of 15° and 28° after 48 hours. The yellow dashed rectangles indicate diffusion channels. (m-o) HAADF images of heterointerface with twist angles 0°, 15° and 28°. (p, q) Compositional evolutions of the atomic ratio of Cl to Br at twisted heterostructure under 150 °C heating from pure Cl region and pure Br region. (r) The evolutions of diffusion channel ratio for different twist angles 0°, 15°, and 28°.

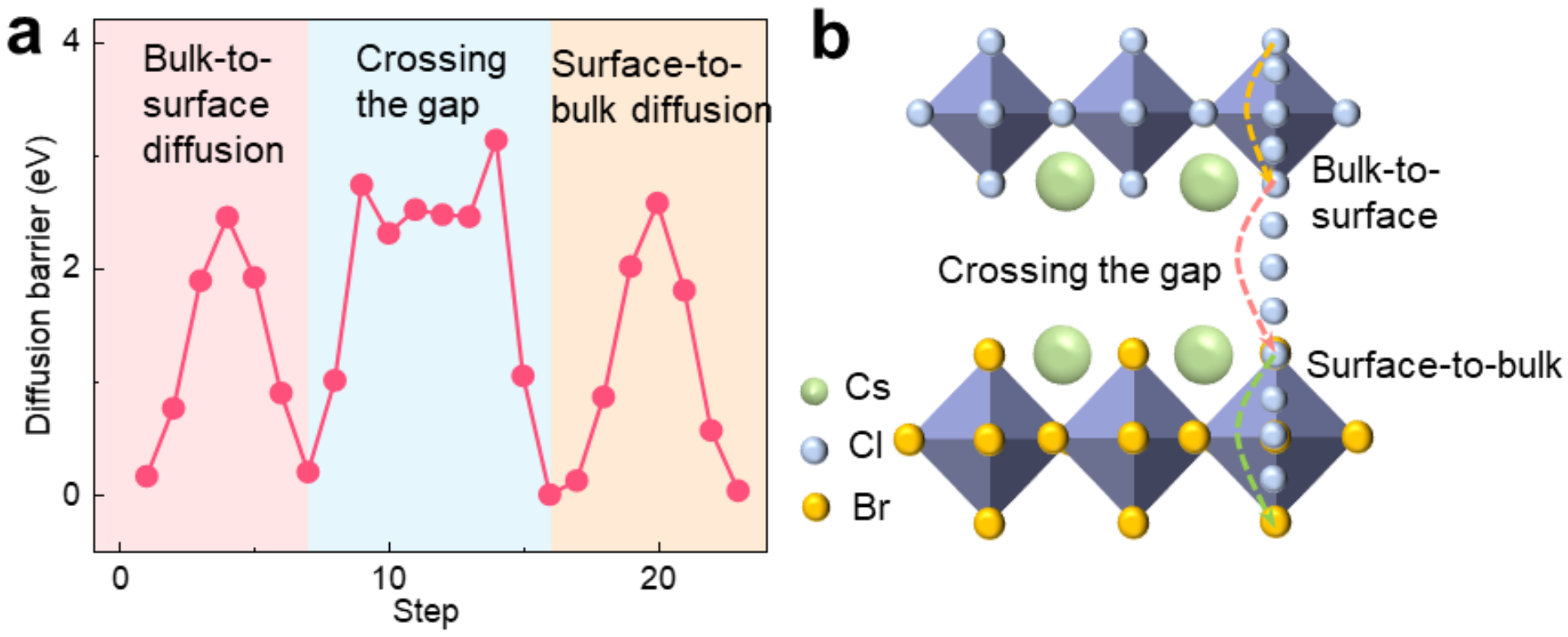


Supplementary Figure 4 | Energy profile and schematic illustration of Cl diffusion across the $CsPbCl_3/CsPbBr_3$ heterostructure. (a) Calculated energy barriers for Cl diffusion in three stages. (b) Schematic representation of the three-stage diffusion pathway, indicating the bulk-to-surface, surface-to-surface (crossing the gapping), and surface-to-bulk processes.

Reviewer #2 (Remarks to the Author):

Review „Twistionics in halide perovskites" by Sanxia Yin et. al.

In this work, the authors investigate ion migration across a halide perovskite heterostructure with and without twist angle. Ion migration at and across the interface of twisted heterostructure is examined in detail on the microscopic level using low-dose TEM. The authors employ molecular dynamics (MD) simulations to corroborate the experimental findings. For the macroscopic scale, complementary transport data (IVCs) are presented. The manuscript provides valuable data that offer insights into the mechanisms of ion migration. The authors' most important finding is that ion migration is dependent on the twist angle, with a minimum at 28°.The experimental results are of high quality and provide new and important insights into ion migration at twist boundaries of halide perovskites, an emerging field of research. Research topic and scientific results of the manuscript appeal to a sufficiently large readership. In principle, the manuscript would be suitable for publication in Nature Communications, however, in its current form it does not quite meet the expected standards, and I therefore propose the following major modifications.

**Reply**: We are grateful for the reviewer's positive comments.

Scientific background: broaden scope by including references to oxide-based perovskites

As the authors correctly state, twistronics is an important research area for van der Waals materials. It is also an emerging field of research for freestanding membranes made of oxide-based perovskites. In order to invite readers stemming from the related field of oxide-perovskites, it would make sense to include references from freestanding oxide community. For example, some recent publications referring to twisted oxide-based perovskites on polar vortices [1,2] and coincident lattice sites [3] could be included. A very recent work on twisted, wafer-bonded ferroelectrics should also not be missed [4]. The observation of a gap at the interface of layer-transferred (hetero-) structures is also well known in the field of freestanding oxide perovskites. Typically, planar surfaces alone are insufficient to achieve intimate contact; instead, an annealing step is often required to form a gapless interface [5] and establish the expected interfacial relaxations at a twist grain boundary [6]. Again, it would be beneficial to the readership to establish a connection to the neighbouring research area of oxide-based perovskites by including some of those references and possibly some work about interdiffusion.

**Reply**: We thank the reviewer for this constructive suggestion. To better bridge our work with the freestanding oxide perovskite community, we have added several recent

references in the revised manuscript. In the Introduction, we have cited studies on polar vortices in twisted $SrTiO_3$ bilayers[6], freestanding $BaTiO_3$ films[7], charge disproportionation at twisted $SrTiO_3$ interfaces[8], and twist-angle-dependent conductivity in wafer-bonded ferroelectrics[9], as well as annealing-driven gapless interfaces[10] and screw-dislocation networks at twisted $SrTiO_3$ interfaces[11].

We have added these words on Line 6-11 Page 4 of the revised manuscript.

Results and discussion: revise proposed model of ion migration by discussing the role of cations

The authors limit their discussion of ion migration to halide ions (anions) and do not specifically address the migration of cations (Cs, Pb). Naturally, halide migration is the primary focus when Cl and Br cross the interface. However, to understand the degradation mechanism at the interface, cation migration must also be considered. Initially, a gap exists at the interface. Over time, this gap is partially closed by nanobridges. These nanobridges ultimately consist of crystalline perovskite material, so the initial local cation depletion in the gap must have been compensated for by cation migration. The experimental results (Fig. S6) and numerical simulations (Fig. 5, in particular Cs seems mobile) support this interpretation. The manuscript would gain significantly in clarity if both halide diffusion and cation migration were discussed.

Furthermore, it can be observed that despite the excess of halide ions in the gap, diffusion into the bulk is limited. Halide ions seem to leak out from bulk into the gap, but a diffusion barrier may exist between the gap and the bulk leading to the halide excess concentration in the gap. Hence, contrary to the authors' description, the gap therefore does not really function as an effective reservoir for halide interdiffusion. The effective reservoir thus remains the bulk itself. However, it is only accessible for interdiffusion after a crystalline bridge across the gap has formed. The manuscript would would gain significantly in clarity if bulk-bulk diffusion and surface-bulk diffusion are clearly distinguished.

One more comment to the 3-step explanation provided by the authors: "(1) … ion migration is dominated by concentration gradients". In my understanding, diffusion always requires concentration gradients. The question is rather, what determines the diffusion constant?

Suggestions for a modified explanation:

1. The experimental results show that halide interdiffusion between bulk components is significantly more efficient in the absence of a gap. As long as a gap exists, i.e., clearly separated surfaces are present, halide interdiffusion is restricted.

Maybe not surprising, but there is an obvious asymmetry in the diffusion direction

and the manuscript would gain clarity if this could be stated with a quantitative estimate, i.e. to compare the diffusion barrier from the gap to bulk with the diffusion barrier from the bulk into the gap.

**Reply**: We thank the reviewer for this careful analysis. We agree that our previous description of the gap as a "reservoir" was not accurate. We have revised the description to clarify that the gap itself is not the reservoir for interdiffusion. Instead, halide ions diffuse from the bulk toward the surface/gap region and accumulate there before effective cross-interface transport occurs.

In addition, we conducted first-principles calculations regarding diffusion barriers (Fig. S4). The diffusion barrier of the Cl diffusing from the $CsPbCl_3$ bulk to its surface is 2.287 eV whereas the barrier for crossing the interfacial gap from the $CsPbCl_3$ surface to the $CsPbBr_3$ surface reaches 3.133 eV. After crossing the gap, the barrier for diffusion from the $CsPbBr_3$ surface into the $CsPbBr_3$ bulk is 2.574 eV. These results quantitatively confirm the asymmetric diffusion process and explain why halide interdiffusion is restricted when clearly separated surfaces exist.

We have revised the relevant description in the manuscript and added a distinction between bulk-to-bulk diffusion and surface-to-bulk diffusion on Page 6 and Line 18-22.

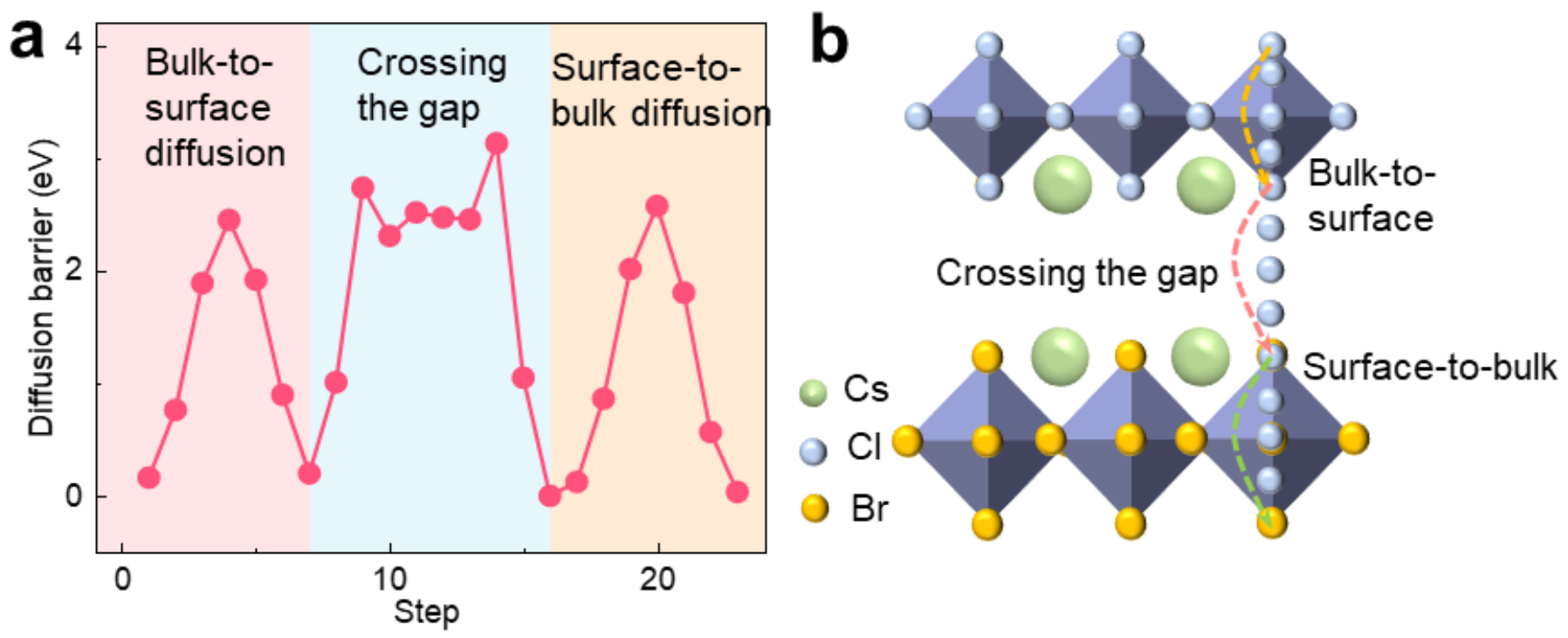


Supplementary Figure 4 | Energy profile and schematic illustration of Cl diffusion across the $CsPbCl_3/CsPbBr_3$ heterostructure. (a) Calculated energy barriers for Cl diffusion in three stages. (b) Schematic representation of the three-stage diffusion pathway, indicating the bulk-to-surface, surface-to-surface (crossing the gapping), and surface-to-bulk processes.

2. For efficient halide interdiffusion, it is necessary to close the gap. This is initiated by the formation of nano-bridges for which “slow” cation migration is crucial. The role of cation migration should be clearly explained. Apparently, nano-bridge formation is triggered by the interaction with the neighbouring crystal and dependent on the strength of those interactions. Interestingly, this interaction can be tuned (locally)

by the twist angle.

As an extreme case of a non-interacting interface, a single crystal (and single surface) could be considered. In this case, ion "surface" migration should be even less than in the case of 28°. The authors may consider to include this in SI.

**Reply**: We thank the reviewer for the comments. We agree that the formation of nanobridges is a prerequisite for efficient halide interdiffusion, and cation migration plays a key role in this process.

Our results show that the initial gap is rich in halide ions but depleted in Cs and Pb (Fig. S3). As the gap closes, cations continuously migrate from both sides toward the interface to compensate for the local Cs/Pb deficiency. MD simulations (Fig. 5) show that Cs ions possess measurable mobility under thermal activation. Halide interdiffusion dominates the initial stage of channel formation, while cation migration provides critical support by compensating charge and facilitating nanobridge crystallization; the synergistic migration of anions and cations accomplishes the interfacial fusion[12,13].

As an extreme case, we examined surface ion migration on a single $CsPbI_3$ nanosheet, where no adjacent crystal is present. Under the same conditions, no appreciable surface diffusion or halide exchange was observed (Fig. S6), indicating that surface ion migration is very limited without interfacial coupling.

We have added this discussion in the revised manuscript and Supplementary Information on page 6 and Line 23-26.

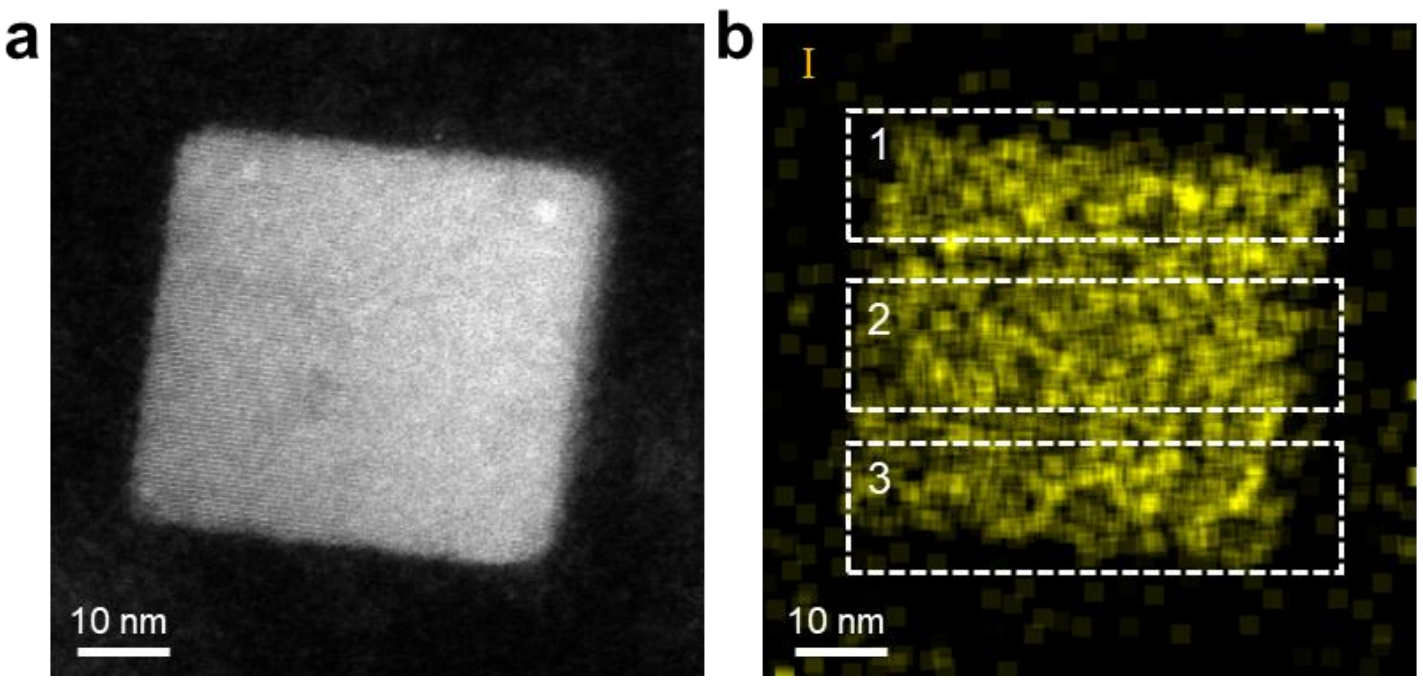


c

| | I/Pb |
|---|---|
| Region 1 | 3.06 |
| Region 2 | 3.05 |
| Region 3 | 2.96 |

Supplementary Figure 6 | Surface ion migration on $CsPbI_3$ nanosheets. (a) HAADF-STEM image of a $CsPbI_3$ nanosheet. (b) The corresponding EDS mapping of I. (c) Table showing the atomic ratio of I : Pb ≈ 3 : 1 within the white dashed box in b.

3. In this context, Fig S3 seems so important that it would be justified to place it into the main text. Only fig S3 clearly shows, how the halide interdiffusion bursts out locally once a nano bridge is formed. This is an absolutely crucial result which is not visible in current fig 2 (see also comments below). Hence, I suggest an alternative fig 2:

**Reply**: We thank the reviewer for this constructive suggestion. We agree that Fig. S3 (newly Fig. 2 in the revised manuscript) clearly shows the local burst of halide interdiffusion once a nanobridge is formed, which is a key result demonstrating that ion migration depends on channel formation. Therefore, we have incorporated the original Fig. S3 into the main text and adjusted the layout of Fig. 2 accordingly. These changes have been made in the revised manuscript.

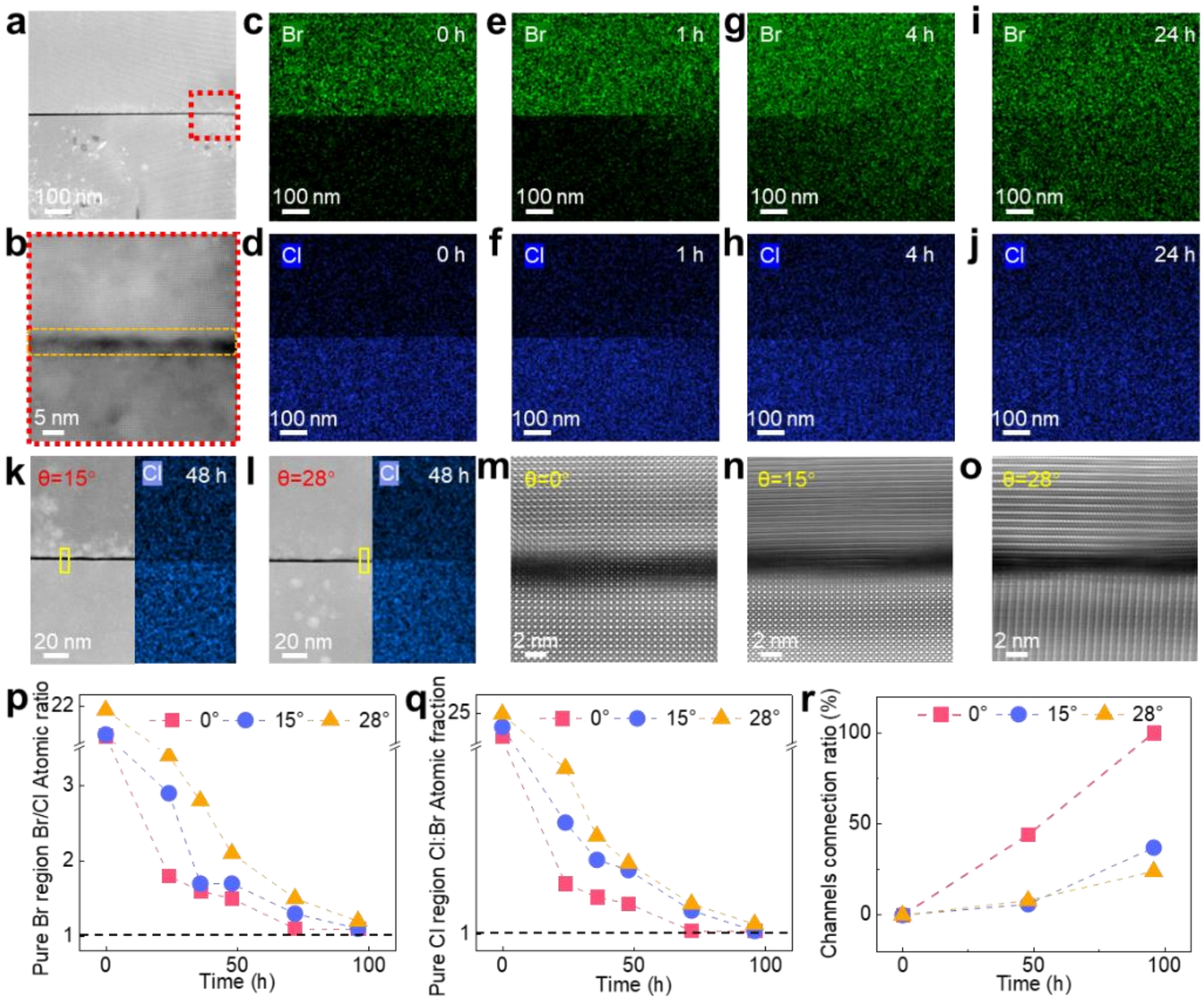

Fig. 2 | Twist-angle-dependent halide composition evolution and diffusion channels in $CsPbCl_3$–$CsPbBr_3$ heterostructures. (a, b) HAADF images of the diffusion channels. (c-j) The corresponding EDS mapping of Br and Cl at 0, 1, 4, and 24 hours. (k, l) HAADF images and corresponding EDS mapping of Cl of the heterostructures with twist angle of 15°, and 28° after 48 hours. The yellow dashed rectangles indicate diffusion channels. (m-o) HAADF images of heterointerface with twist angles 0°, 15° and 28°. (p, q) Compositional evolutions of the atomic ratio of Cl to Br at twisted heterostructure under 150 °C heating from pure Cl region and pure Br region. (r) The evolutions of diffusion channel ratio for different twist angles 0°, 15°, and 28°.

According to fig 4h, MD simulations were performed for twist angles 0°, ≈ 18°, ≈ 28°, ≈ 35°, and 45°. It would be good, if the authors could state the exact angles.

Notably, the experimental twist angle of 15° is missing and it is surprising that the experimental twist angle has not been considered in MD simulations. Could the authors explain and justify the particular set of twist angles used in MD simulations? Periodic boundary conditions..?

As a side note, it may help the reader to explicitly state that for cubic symmetry the range of twist angles 0° - 45° is sufficient to investigate.

**Reply**: Great appreciation for your professional comments. We have now specified the exact twist angles used in the simulations: 0°, 18.92°, 28.07°, 36.87°, and 43.60°.

These angles were selected because periodic boundary conditions require commensurate supercells for the twisted square lattice. The allowed twist angles can be described by $\theta = 2\arctan(m/n)$ where m and n are coprime integers. The twist system comprises $2(m^2 + n^2)$ unit cells. The (m, n) integer pair and the corresponding twist angles are presented in the following table.

For the experimental twist angle of ~15°, the closest commensurate model corresponds to (5,38), which contains 2938 unit cells and 14690 atoms. This system size is beyond our first-principles computational capability.

We have also clarified that, due to the fourfold symmetry of the cubic lattice, twist angles from 0° to 45° are sufficient to represent the distinct interfacial configurations.

We have added these explanations in the Supporting Information.

Supplementary Table 1 | The (m, n) integer pair and the corresponding twist angles.

| m | n | θ (degree) |
|---|---|---|
| 1 | 6 | 18.92 |
| 1 | 5 | 22.61 |
| 1 | 4 | 28.07 |
| 1 | 3 | 36.87 |
| 2 | 5 | 43.60 |
| 5 | 38 | 14.99 |

What is so special about 28°? From crystal symmetry (cubic) this is not obvious. While 28° degrees do not coincide with any symmetry axis of the considered crystals, it might match with coincident site lattice (CSL). With the simulations at hand, the authors should provide some scientific insight.

**Reply**: We thank the reviewer for this important question. We agree that 28° is not

a special symmetry angle of the cubic lattice. Its significance in our work arises from the strongly reduced interfacial registry at this twist angle.

Consider a fixed atomic layer and a twisted layer. The twisted atom coordinates (x, y) lie on the unit circle ($x^2+y^2=1$, $0 \leq x, y \leq 1$, $x \geq y$). We examine the distances from the twisted atom to three nearest-neighbor positions in the fixed layer: A (0.5,0.5), B (1,0), and C (1,1). The variance of these three distances is minimized when the twist angle is approximately 28°, as determined by numerical optimization. At this angle, the twisted atom lies near the center of the triangle formed by A, B, and C, maximizing its average separation from the fixed layer and thus minimizing van der Waals coupling. This angle represents the opposite of a coincident site lattice (which seeks atomic coincidence) and instead maximizes atomic misalignment, effectively suppressing ion migration.

We have added this discussion in the Supporting Information.

Smaller comments:

Page 4: "Recently, twistionics, the deliberate control …" Here, it is about twistronics. Please correct.

**Reply**: We thank the reviewer for the correction. "twistionics" has been changed to "twistronics". The correction has been made in the revised manuscript.

Page 6: Discussion of twist-angle-dependent interdiffusion of halide ions, fig 2a-c). The start of the discussion does not clearly state, that this is for twist angle 0°. Please make this more clearly.

**Reply**: We thank the reviewer for the comment. It has been clarified in the main text that Fig. 2a-c correspond to the sample with a twist angle of 0°. The correction has been made in the revised manuscript.

Page 7: "meanwhile, statistical analysis …" What kind of analysis? From experimental TEM data or numerical simulations? Please clarify.

**Reply**: We thank the reviewer for the comment. The statistical analysis is based on the quantification of diffusion channels observed in experimental TEM images, rather than numerical simulations. We have clarified this in the revised manuscript on page 7 and Line 14.

Page 7: "… aligned and unaligned feature, as indicated in Fig S7… diffusion channels prefer to be formed where the lattice … aligns." Whether the lattices are aligned or misaligned is not clearly visible in the figure. Further, fig S7 claims that half a unit cell misalignment also yields efficient ion migration? Maybe, the authors could

find a better way to present the experimental results. See also comment to Fig S7 below

**Reply**: We thank the reviewer for the suggestion. We have therefore reprocessed the figure and enlarged the aligned and misaligned regions for better visibility (Fig. S12).

We also clarified in the figure caption that the "lattice-matched" regions include both fully aligned and half-unit-cell shifted configurations. In these regions, the two lattices still maintain periodic registry across the interface, which can support the formation of diffusion channels.

We have added this explanation in the revised manuscript and the caption of Fig. S12 and S13.

Page 8: "Considering that lattice mismatch induced by twist angle …" This sounds a bit confusing, because "mismatch" associates with mismatch of lattice parameter. Maybe one could write "lattice misalignment"?

**Reply**: We thank the reviewer for the suggestion. The original wording was indeed ambiguous. We have changed "lattice mismatch" to "lattice misalignment" in the revised manuscript.

Page 8, ref [40] Is this the correct reference for ion migration? Ref 40 "DFT study on type-II photocatalyst for overall water splitting …" discusses only electronic charge carrier migration and does not mention ion migration at all.

**Reply**: We thank the reviewer for the correction. Upon checking, reference [40] is indeed inappropriate. We have replaced it with an appropriate reference on ion migration[14]. The correction has been made in the revised manuscript.

Page 9, "For the 0° device, it is observed a significant rectifying behaviour…" Language! Please improve.

**Reply**: We thank the reviewer for the comment. The original sentence had a grammatical issue. We have revised it to "For the 0° device, a significant rectifying behavior is observed…" The correction has been made in the revised manuscript on page 6 and Line 6.

Fig1, caption: "(c) Optical images of twisted the heterostructures …" Typo, please skip the "the".

**Reply**: We thank the reviewer for pointing out the typo. In the revised manuscript, we have deleted "the".

Fig2,

6. Yellow boxes to indicate diffusion channels: On that scale and resolution, we simply cannot identify diffusion channels or nano-bridges. Maybe, a high-resolution image would show them, but on the chosen larger range the reader cannot see those. I do not think that inserting "yellow boxes" as a guide to the eye is the way to go... Anyway, I suggest to rework fig 2, see above.

**Reply**: We thank the reviewer for this useful suggestion. We have therefore provided high-magnification images of the corresponding regions in the Supplementary Information (Fig. S9), where the local nano-bridge/diffusion-channel features can be more clearly resolved.

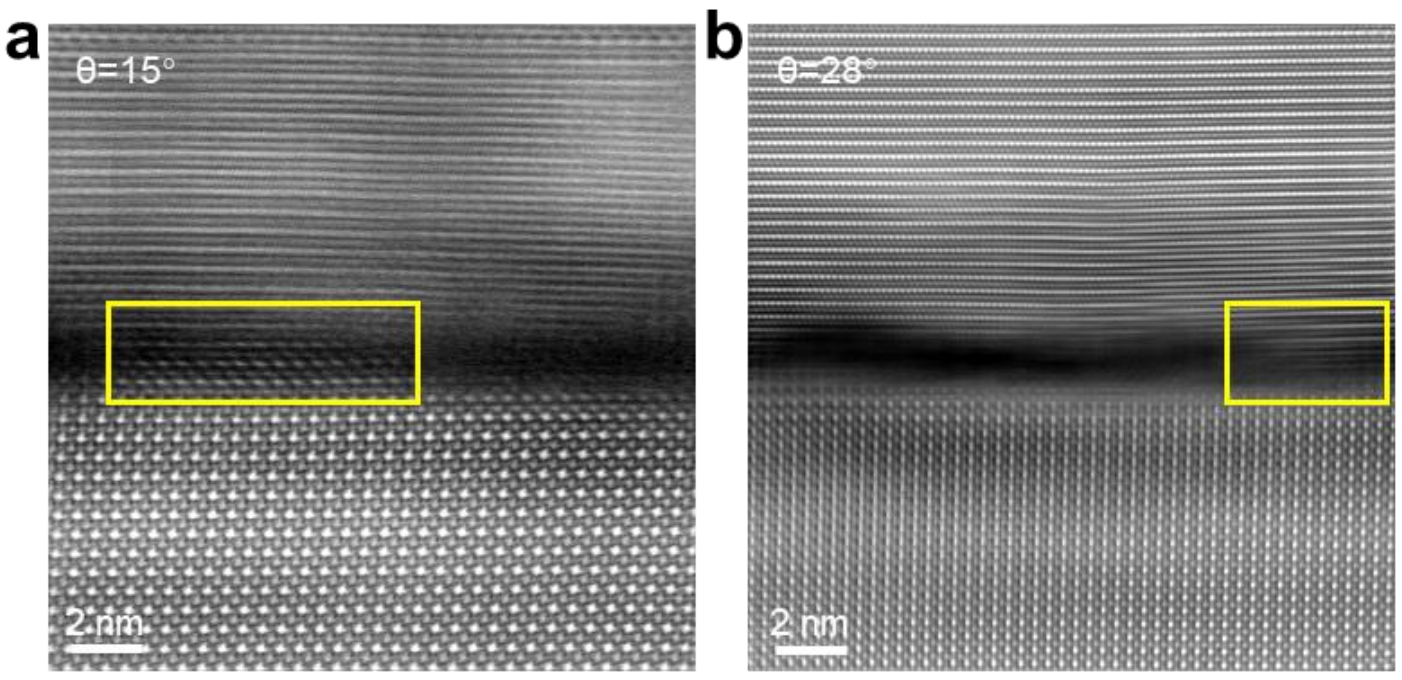


Supplementary Figure 9 | High-magnification HAADF-STEM images of the areas indicated by yellow boxes in Fig. 2. (a) 15° twist; (b) 28° twist.

7. Caption: false enumeration (It is correct in the text): (m,n) => (j,k), (j-l) => (l-n)

**Reply**: We thank the reviewer for the correction. As Fig. 2 has been reorganized according to the suggestion, we have updated all the panel labels in the caption to ensure consistency with the main text.

8. Text within sub-figures is very small. When text is placed on non-uniform background, such as atomic resolution TEM, then one cannot read the text without significant enlargement. Well, this is possible only in the digital version…

**Reply**: We thank the reviewer for the comment. We have increased the font size of the labels and placed them in regions with uniform background contrast wherever possible to improve readability.

Fig3, also same problem with text as in fig2: too small…

**Reply**: We thank the reviewer for the comment. We have accordingly increased the font size of the labels in Fig. 3 and placed them in regions with uniform background where possible.

Fig4:

It would help if row d-f could be separated more clearly from row a-c. Currently, the gap with the halide heterostructure is similar as the gap between figure a and figure d! This makes it difficult to read.

**Reply**: We thank the reviewer for the comment. We have increased the spacing between the (a–c) and (d–f) panels in Fig. 4 to help readers clearly distinguish the two groups.

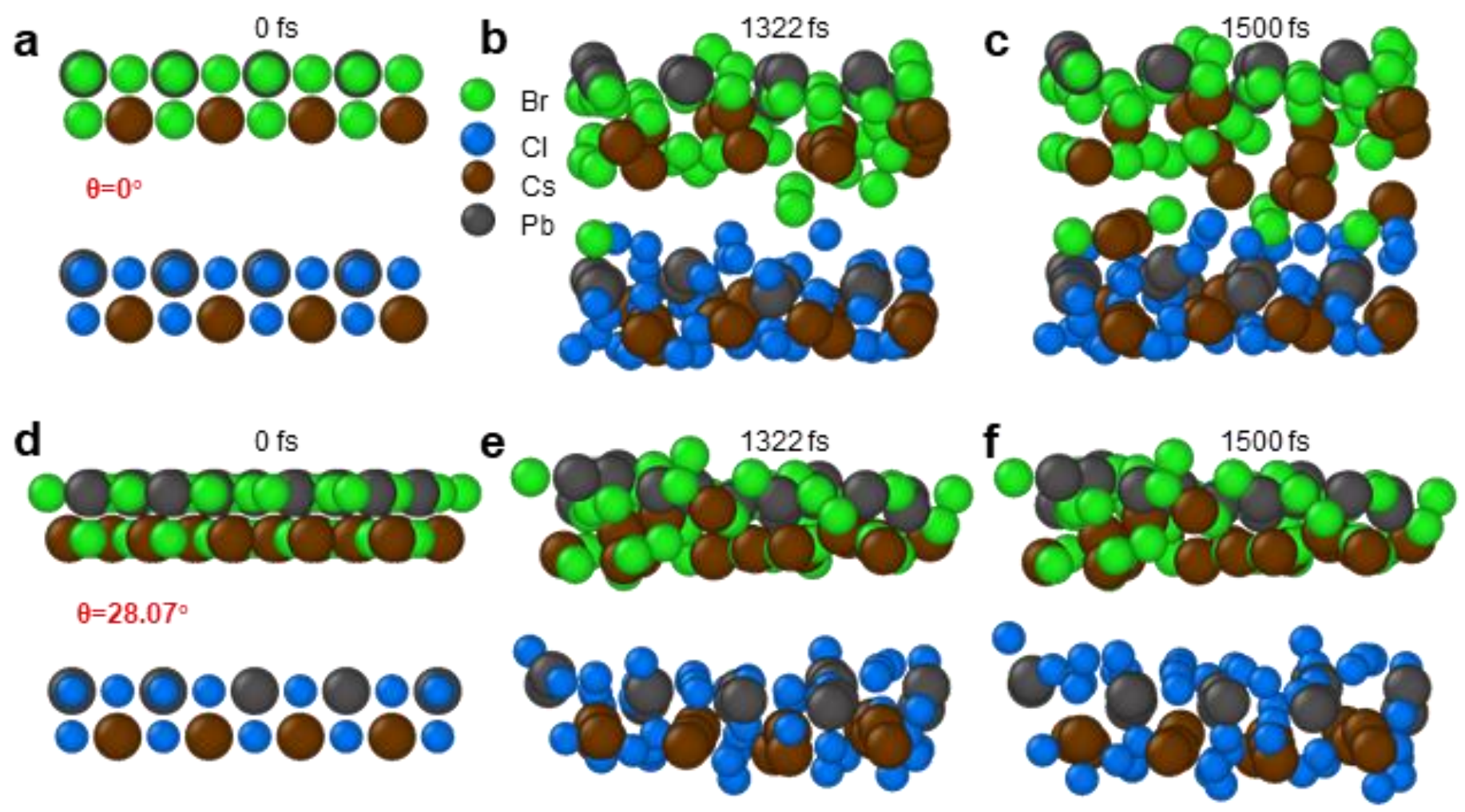


Fig 5

Fig 5a): What is the little doted square? Please clarify or remove.

**Reply**: We thank the reviewer for pointing this out. The small dotted square in Fig. 5a indicates the position where the PL spectra were acquired (the $CsPbBr_3$ side). We have clarified this in the figure caption.

The $CsPbCl_3$-film is grown on STO as described in methods. But fig 5b) c) actually indicate that there are $CsPbBr_3$- flakes and $CsPbCl_3$-flakes. A double transfer process is not described in the methods section, and should probably be completed, accordingly. Yet, it may suggest that the substrate for the devices is indeed something else than STO. Probably not only Si, but $SiO_2$/Si? Please clarify and correct accordingly.

The CsPbCl3 film is deposited onto STO as described in the methods. However, Figures 5b) and c) show $CsPbBr_3$ and $CsPbCl_3$ flakes. A double transfer process is not described in the methods and should therefore be added. Further, this suggests that the substrate of the devices is not the original growth substrate STO, and it could be Si as indicated in the figure. Yet, I suspect it is rather $SiO_2$/Si. Please clarify.

**Reply**: We thank the reviewer for the comment. The description of the device substrate and transfer process in the Methods section was indeed insufficiently clear. We have now added a detailed transfer process: the $CsPbCl_3$ film was first transferred

onto a thermally oxidized $SiO_2/Si$ substrate; subsequently, the $CsPbBr_3$ film was transferred onto the $CsPbCl_3/SiO_2/Si$ stack. The twist angle between the two films was controlled by aligning their edges under an optical microscope. Thus, the final device substrate is $SiO_2/Si$, not STO. The $CsPbBr_3$ and $CsPbCl_3$ flakes shown in Fig. 5(b, c) are the components of the heterostructure formed after this sequential transfer.

The corresponding revisions have been made in the Methods section.

The dotted lines are probably supposed to highlight the edges of the $CsPbBr_3$-flake. I would prefer to skip the dotted lines since this just obscures the optical image and gives a false impression. For example, the dotted boxes suggests that the flake is rectangular while the image shows it is not perfectly rectangular. If it is really necessary to highlight the $CsPbBr_3$ flake, one could opt for false colour.

**Reply**: We thank the reviewer for the suggestion. We agree that the dashed boxes in the original figure were distracting and gave a false impression of perfectly rectangular flakes. We have removed the dashed boxes and instead used a translucent light blue overlay to highlight the $CsPbBr_3$ flakes in the optical images. This clearly indicates the flake positions without distorting the actual morphology. The corresponding modifications have been made in Fig. 5(b, c) of the revised manuscript.

Supplementary material

Fig S2:

It would improve readability if the separate lines would not overlap. Maybe, one could consider separate graphs for each atomic species?

**Reply**: We thank the reviewer for the suggestion. To avoid overlapping and improve readability, we have separated the spectral lines in Fig. S2 (newly Fig. S3) and plotted individual graphs for each atomic species. The figure has been replaced in the revised Supporting Information.

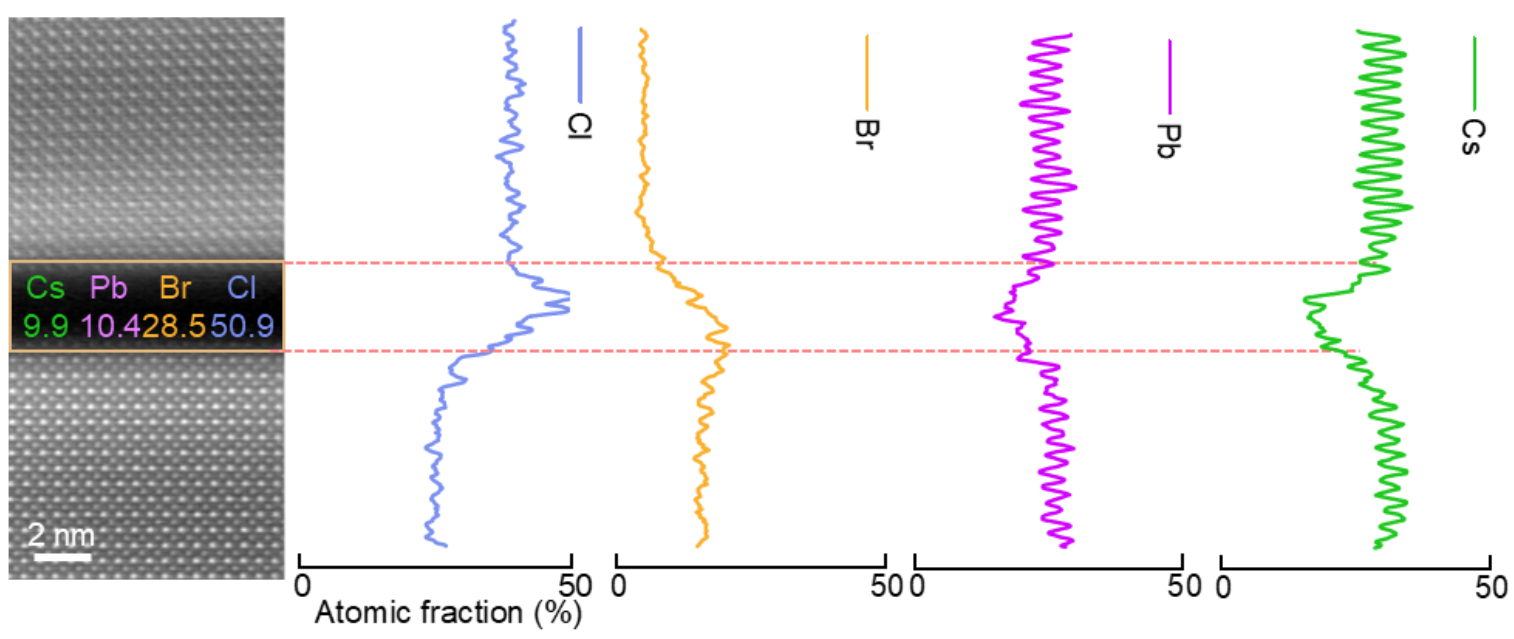


Supplementary Figure 3 | HAADF image and quantitative element analysis at the initial heterointerface with twist angle of 0°.

Fig S3-8

Please indicate the twist angle! I assume it is 0°, but it is nowhere clearly stated.

**Reply**: We thank the reviewer for pointing this out. The twist angle (0°) has now been clearly indicated in the relevant figure and specified in the figure caption. The correction has been made in the revised Supporting Information.

Fig S4:

a) Please skip the dotted boxes.

**Reply**: We thank the reviewer for the suggestion. The dotted boxes have been removed from Supplementary Fig. 7a.

b) Please skip the dotted lines. It seems they are just qualitative guides to the eye, which do not add really anything.

**Reply**: We thank the reviewer for the suggestion. The dotted lines have been removed from Supplementary Fig. 7b.

b) Please skip the orange dotted line. Again, this is just a guide to the eye. This time, without to explain the criteria, i.e. 50% Br? Please, either explain clearly how this line is defined, or skip. Even without guides to the eye the main message is clear: the gap is closing and a crystalline interface is formed, allowing for efficient halide interdiffusion.

**Reply**: We thank the reviewer for the suggestion. We agree that the orange dotted line was simply a guide to the eye without a clear definition criterion. It has been removed. The modification has been made in the revised Supporting Information.

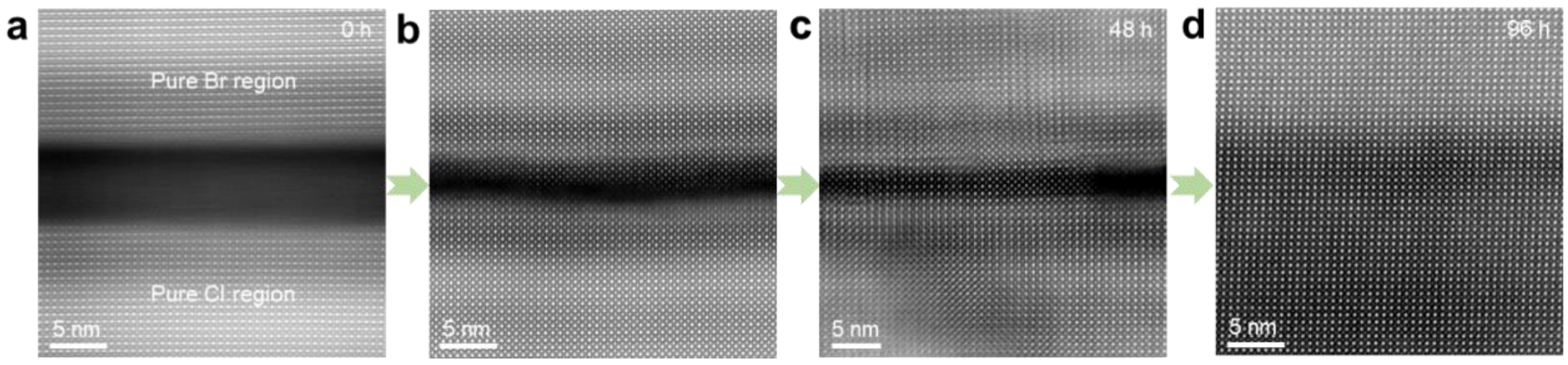


Supplementary Figure 7 | Atomic-scale observation of ion migration at the heterointerface with twist angle of 0°. (a-d) HAADF images presenting the connection process at the heterointerface stored in vacuum. The orange dashed lines roughly indicate the surface of pure-Br region

Fig S7

a) – c): Aligned and unaligned regions are marked, but the reader has no chance to independently verify this: too small image, too small range …

**Reply**: We thank the reviewer for the comment. We have reprocessed the figure and enlarged the display area to facilitate independent judgment by readers. The replacement has been made in the revised manuscript.

(g-i) Shift of half a unit cell is not clearly visible. In any case, does it mean, that also an unaligned lattice forms nanobridges?

**Reply**: We thank the reviewer for the comment.

We have therefore enhanced the image contrast in panels (g–i) and added atomic models below these panels to better illustrate the local interfacial registry and diffusion pathway.

We would like to clarify that the half-unit-cell shift does not represent a fully unaligned interface. Although the two lattices are laterally shifted, they still share the same crystallographic orientation and maintain periodic registry across the interface. Therefore, both fully aligned and half-unit-cell shifted regions are considered lattice-matched regions, where nano-bridges preferentially nucleate.

For regions with weaker local registry, nano-bridge formation can still occur, but with slower kinetics. For example, in the 0° sample, the entire interface becomes connected after storing in vacuum for 96 h. We have clarified this point in the caption of Supplementary Fig. S13.

(g-i) What does APB mean? Please explain.

**Reply**: We thank the reviewer for the question. APB stands for anti-phase boundary, referring to regions where the lattice phase is inverted by 180° at the interface[15]. We have added this definition in the caption of the supplementary figure.

Reviewer #3 (Remarks to the Author):

This is an interesting study that looks at inter-diffusion of ions from a block of $CsPbBr_3$ to a block of $CsPbCl_3$ perovskite as a function of the twist angle between the blocks. In this study two crystals are separated by a 4 nm gap and the authors carry out an elegant imaging study showing the formation of ion bridges and ultimately the melding of the two interfaces. It makes sense that introducing a twist in the lattice at the interface makes it harder for ions to pass across. The work is interesting and I think publishable in subject to some revisions:

**Reply**: We are grateful for the reviewer's positive comments.

1. It is stated that ion migration is 'a fundamental instability' in halide perovskites, and that ion migration in PV leads to 'reduced open circuit voltage'. The argument lacks nuance! Ion migration can be bad for halide perovskites, although there are some cases when it can improve design tolerances and in PV ions tend to increase voltage and decrease current so it is not correct to say that they just straightforwardly reduce open circuit voltage and in fact the picture is complex (see e.g. Hart et al Energy Environ. Sci., 2024,17, 7107-7118; M. Córdoba and K. Taretto, Solar RRL, 2023, 8 , 2300742 and a nuanced discussion in ACS Energy Lett. 2025, 10, 10, 4849–4855)

**Reply**: We thank the reviewer for this comment. The original statement was overly simplified. The effect of ion migration on the performance of perovskite photovoltaic devices is indeed complex: it can either reduce open-circuit voltage or, under certain conditions, improve it. For example, Hart et al. found that mobile ions can significantly increase the steady-state open-circuit voltage and improve device tolerance to interfacial energy level misalignment[16]. Córdoba and Taretto reported that increasing the interface band offset can enhance the open-circuit voltage in inorganic perovskite solar cells containing mobile ions[17]. Schmidt and Ehrler pointed out that ion accumulation at the interface can screen the built-in electric field, and its effect on open-circuit voltage depends on the device band alignment, which may either increase, decrease, or have no effect[18].

We have revised the relevant statement and added these literatures in the Introduction accordingly.

2. Throughout the paper it is discussed that ion migration is strongly supressed in twisted systems. I think the authors should be more precise with their language here – ion migration can and will still occur within both the $CsPbBr_3$ and $CsPbCl_3$, either under illumination or applied voltage. Ions will still move to the interfaces in the devices described in figure 5, presumably with some initial ion accumulation at the perovskite-

perovskite interface depending on the built in potential difference across that interface. This doesn't change much in the twisted systems, the difference is that it is then harder for ions to pass from the bromide perovskite to the chloride one (and/or vice versa). What is being controlled is that exchange of halide from on material to another, there will still be ion migration in each parent material.

**Reply**: We thank the reviewer for this comment. The original statement was indeed not precise enough. The twist angle suppresses the cross-interface halide ion exchange (i.e., migration from one perovskite to the other), rather than ion migration within each individual layer. In the twisted system, ion migration within the bulk of $CsPbBr_3$ and $CsPbCl_3$ still exists, and ions can still migrate to the interface.

We have revised the wording throughout the manuscript to clarify that twist-angle engineering suppresses cross-interface halide interdiffusion, rather than completely suppressing ion migration in the parent perovskite materials.

3. I struggled a lot to see the images in Figure 2 that show 'diffusion channels' I think they need to be larger/zoomed in. By eye the channels are not clearly visible in the PDF. I'm also interested in the mechanism – e.g. the step 1 formation of 'interfacial diffusion channels' followed by lateral growth. The interface evolution is shown in wonderful detail. What I missed however is a discussion of the relevance to devices where there is not a 4 nm gap. Will the mechanism be the same? I also missed a discussion on the relative changes in diffusion rate for the twisted interfaces, compared to the many papers where diffusion is interrupted e.g. by a 2D layer as the interface or slowed down by e.g. cation substitution.

**Reply**: We thank the reviewer for these helpful comments.

First, we have reorganized Fig. 2 to better show the burst of interdiffusion once nanobridge formation, and provided high-magnification images of the corresponding regions in Fig. S9.

Second, we have clarified the relevance of this mechanism to practical devices. Although real devices usually do not contain an initial ~4 nm gap[19], the gap-containing interface in our experiment captures an early stage of interfacial evolution from separated surfaces to connected interfaces. A no-gap device interface can be regarded as a later stage of this process, where the obvious gap-closing step is no longer required. Nevertheless, efficient cross-interface halide exchange still depends on the formation and lateral continuity of interfacial diffusion pathways. Twisting weakens interfacial registry and coupling, thereby suppressing the formation and connection of these pathways.

Third, we have added a discussion comparing this twist-angle strategy with

previous approaches such as 2D interlayers and compositional substitution. In our experiments, the 28° twist angle reduces the halide interdiffusion rate by about 25% compared to the 0° sample (Fig.2p, q). This value is obtained from FIB-thinned lamella samples, likely faster than that in actual thick devices. Previous studies suggest substitution strategies can reduce $I^-$ mobility by more than an order of magnitude[20], while 2D layer strategies can prevent noticeable halide interdiffusion for several days[21]. The significance of our work is that twist-angle engineering offers a distinct, interface-geometry-based route to regulate ion transport without changing the intrinsic material composition or introducing foreign blocking layers. This strategy is therefore complementary to existing ion-migration suppression methods.

We have added these discussions on Line 18-21 at Page 10 and Line 14-19 at Page 11 of the revised manuscript.

4. Finally I missed a discussion on how twistionics could be routinely and reproducibly introduced to devices? The paper fully lacks an experimental and methodology section, this needs to be added in full before publication. In addition it would be good to add more on how the electron beam is influencing the growth of interfacial structures, and how the rate of interdiffusion under imaging conditions compares to that under operating conditions for a device.

**Reply**: We thank the reviewer for these helpful comments.

First, we have added a detailed Methods section describing how twistionics is introduced into devices. Briefly, the $CsPbCl_3$ film was first transferred onto a thermally oxidized $SiO_2$/Si substrate, followed by the $CsPbBr_3$ film. The twist angle was controlled by aligning the crystal edges under an optical microscope before transfer, which allows routine construction of twisted perovskite heterostructures.

Second, we have also added a discussion on the role of the electron beam. The electron beam can transfer energy to materials[3], locally activate ion migration and interfacial structural evolution. A higher beam current may accelerate nano-bridge formation and interfacial growth; however, excessive electron dose can induce perovskite decomposition. Therefore, electron-beam-assisted in situ observation effectively mimics ion migration behavior under device operating conditions.

Third, we have clarified the comparison between imaging conditions and device operating conditions. Ion migration under real operating conditions involves coupling of thermal, electric, and optical fields and is more complex than microscopic observations. In our experiments, we used thermal annealing at 150 °C to accelerate ion diffusion to obtain measurable halide interdiffusion within a practical time window. Although the absolute interdiffusion rates under electron-beam imaging, thermal

annealing, and real device operation are different, they consistently show the same trend: twisted interfaces suppress cross-interface halide exchange compared with lattice-aligned interfaces.

We have added these discussions on Line 8-9 Page 8 and Line 2-6 Page 10 of the revised manuscript.

5. The van der waals interactions mentioned are interesting, the paper would benefit from more discussion on this.

**Reply**: We thank the reviewer for this comment. We have expanded the discussion on the role of van der Waals interactions in the revised manuscript.

Previous studies have shown that vdW interactions are highly sensitive to interfacial distance and atomic registry[23,14]. For, example, first-principles calculations on 2D MXenes show that vdW interactions directly affect the interlayer spacing and binding energy[24] while AFM measurements confirmed that vdW forces increase significantly as the tip-sample distance decreases[25]. In addition, twisted bilayer systems such as $MoS_2$ have demonstrated that twist angle can modulate interlayer vdW coupling[14].

Based on these insights, we further discuss our three-stage diffusion model. As the interfacial gap gradually decreases from several nanometers to near-contact, vdW interactions between the two perovskites continuously increase (Fig. 4g), promoting nanobridge formation. Once nano-bridges form, they provide connected pathways for cross-interface halide interdiffusion. The twist angle modulates vdW coupling by changing local atomic registry: at 0°, atoms are aligned and vdW coupling is strong; at 28.07°, misalignment leads to a ~ 50% decrease in vdW energy as shown by DFT calculations, explaining the reduced nano-bridge formation and suppressed halide exchange.

We have added these discussions on Line 3-7 Page 9 and Line 10-12 Page 9 of the revised manuscript.

**Reference:**


1. Eames, C. et al. Ionic transport in hybrid lead iodide perovskite solar cells. *Nat. Commun.* **6**, 7497 (2015).

2. Liu, S. et al. Epitaxy of a monocrystalline $CsPbBr_3$-$SrTiO_3$ halide-oxide perovskite p–n heterojunction with high stability for photodetection. *Adv. Mater.* **35**, 2303544 (2023).

3. Egerton, R. F. et al. Radiation damage in the TEM and SEM. *Micron* **35**, 399–409 (2004).

4. Israelachvili, J. N. The nature of van der waals forces. *Contemp. Phys.* **15,** 159–178 (1974).

5. Fineman, M. A. et al. Heats of formation and some derived thermodynamic properties of NaCl-NaBr solid solutions[1,2]. *J. Am. Chem. Soc.* **70**, 4165–4169 (1948).

6. Sha, H. et al. Polar vortex hidden in twisted bilayers of paraelectric $SrTiO_3$. *Nat. Commun.* **15**, 10915 (2024).

7. Sánchez-Santolino, G. et al. A 2D ferroelectric vortex pattern in twisted $BaTiO_3$ freestanding layers. *Nature* **626**, 529–534 (2024).

8. Kim, M.-S. et al. Charge disproportionation at twisted $SrTiO_3$ bilayer interface driven by local atomic registry. *ACS Nano* **19**, 39714–39724 (2025).

9. Rogers, A. et al. Polar discontinuities, emergent conductivity, and critical twist-angle-dependent behaviour at wafer-bonded ferroelectric interfaces. *Nat. Commun.* **17**, 1842 (2026).

10. Wang, H., et al. Interface design beyond epitaxy: Oxide heterostructures comprising symmetry-forbidden interfaces. *Adv. Mater.* **36**, 2405065 (2024).

11. Schmidbauer, M. et al. Periodic lateral superlattice in bonded $SrTiO_3/SrTiO_3$ twisted perovskites. *Appl. Phys. Lett.* **126**, 101902 (2025).

12. Moral, R. F. et al. Anion and cation migration at 2D/3D halide perovskite interfaces. *ACS Energy Lett.* **9**, 2703–2716 (2024).

13. Li, W. et al. The role of ion migration, octahedral tilt, and the A-site cation on the instability of $Cs_{1-x}FA_xPbI_3$. *Nat. Commun.* **14**, 8523 (2023).

14. Wu, Y. et al. Observation of an intermediate state during lithium intercalation of twisted bilayer $MoS_2$. *Nat. Commun.* **13**, 3008 (2022).

15. Chen, S. et al. Atomic structure and electrical/ionic activity of antiphase boundary in $CH_3NH_3PbI_3$. *Acta Mater.* **234**, 118010 (2022).

16. Hart, L. J. F. et al. More is different: mobile ions improve the design tolerances of perovskite solar cells. *Energy Environ. Sci.* **17**, 7107–7118 (2024).

17. Córdoba, M. et al. Insight into the dependence of photovoltaic performance on interfacial energy alignment in solar cells with mobile ions. *Solar RRL* **8**, 2300742 (2024).

18. Torre Cachafeiro, M. A. et al. Ionic losses and gains in perovskite solar cells: impact on efficiency and stability. *ACS Energy Lett.* **10**, 4849–4855 (2025).

19. Zhou, Y. et al. Heterojunction structures for reduced noise in large-area and sensitive perovskite x-ray detectors. *Sci. Adv.* **7**, eabg6716 (2021).

20. Ferdani, D. W. et al. Partial cation substitution reduces iodide ion transport in lead iodide perovskite solar cells. *Energy Environ. Sci.* **12**, 2264–2272 (2019).

21. Hautzinger, M.P. et al. Metal halide perovskite heterostructures: Blocking anion diffusion with single-layer graphene. *J. Am. Chem. Soc.* **145,** 2052–2057 (2023).

22. Thiesbrummel, J. et al. Ion migration in perovskite solar cells. *Nat. Rev. Chem.* **10**, 179–195 (2026).

23. Luo, Y. et al. Van der Waals interactions at the nanoscale: The effects of nonlocality. *Proc. Natl. Acad. Sci. U.S.A.* **111**, 18422–18427 (2014).

24. Rajashekhar Vaibhava, K. M. et al. Investigation of van der Waals interactions in two-dimensional MXenes via first-principles simulations. *Phys. Chem. Chem. Phys.* **28**, 2090–2102 (2026).

25. Gisbert, V. G. et al. Fast and high-resolution mapping of van der Waals forces of 2D materials interfaces with bimodal AFM. *Nanoscale* **15**, 19196–19202 (2023).

**Review „Twistionics in halide perovskites" by Sanxia Yin et. al.**

In this work, the authors investigate ion migration across a halide perovskite heterostructure with and without twist angle. Ion migration at and across the interface of twisted heterostructure is examined in detail on the microscopic level using low-dose TEM. The authors employ molecular dynamics (MD) simulations to corroborate the experimental findings. For the macroscopic scale, complementary transport data (IVCs) are presented.

The manuscript provides valuable data that offer insights into the mechanisms of ion migration. The authors' most important finding is that ion migration is dependent on the twist angle, with a minimum at 28°.

The experimental results are of high quality and provide new and important insights into ion migration at twist boundaries of halide perovskites, an emerging field of research. Research topic and scientific results of the manuscript appeal to a sufficiently large readership. In principle, the manuscript would be suitable for publication in Nature Communications, however, in its current form it does not quite meet the expected standards, and I therefore propose the following major modifications.

**Scientific background: broaden scope by including references to oxide-based perovskites**

As the authors correctly state, twistronics is an important research area for van der Waals materials. It is also an emerging field of research for freestanding membranes made of oxide-based perovskites. In order to invite readers stemming from the related field of oxide-perovskites, it would make sense to include references from freestanding oxide community. For example, some recent publications referring to twisted oxide-based perovskites on polar vortices [1,2] and coincident lattice sites [3] could be included. A very recent work on twisted, wafer-bonded ferroelectrics should also not be missed [4].

The observation of a gap at the interface of layer-transferred (hetero-) structures is also well known in the field of freestanding oxide perovskites. Typically, planar surfaces alone are insufficient to achieve intimate contact; instead, an annealing step is often required to form a gapless interface [5] and establish the expected interfacial relaxations at a twist grain boundary [6]. Again, it would be beneficial to the readership to establish a connection to the neighbouring research area of oxide-based perovskites by including some of those references and possibly some work about interdiffusion.

---

[1] Sha, H., Zhang, Y., Ma, Y., Li, W., Yang, W., Cui, J., ... & Yu, R. (2024). Polar vortex hidden in twisted bilayers of paraelectric SrTiO3. *Nature Communications*, *15*(1), 10915.
[2] Sánchez-Santolino, G., Rouco, V., Puebla, S., Aramberri, H., Zamora, V., Cabero, M., ... & Santamaría, J. (2024). A 2D ferroelectric vortex pattern in twisted BaTiO3 freestanding layers. *Nature*, *626*(7999), 529-534.
[3] Kim, M. S., Lee, K., Ishikawa, R., Song, K., Shahed, N. A., Eom, K. T., ... & Choi, S. Y. (2025). Charge Disproportionation at Twisted SrTiO3 Bilayer Interface Driven by Local Atomic Registry. *ACS nano*, *19*(46), 39714-39724.
[4] Rogers, A., Holsgrove, K., Schäfer, N. A., Koppitz, B., McCluskey, C. J., Yedama, S., ... & Gregg, J. M. (2026). Polar discontinuities, emergent conductivity, and critical twist-angle-dependent behaviour at wafer-bonded ferroelectric interfaces. *Nature Communications*.
[5] Wang, H., Harbola, V., Wu, Y. J., van Aken, P. A., & Mannhart, J. (2024). Interface Design beyond Epitaxy: Oxide Heterostructures Comprising Symmetry-Forbidden Interfaces. *Advanced Materials*, *36*(32), 2405065.
[6] Schmidbauer, M., Maltitz, J., Stümpel, F., Hanke, M., Richter, C., Schwarzkopf, J., & Martin, J. (2025). Periodic lateral superlattice in bonded SrTiO3/SrTiO3 twisted perovskites. *Applied Physics Letters*, *126*(10).

**Results and discussion: revise proposed model of ion migration by discussing the role of cations**

The authors limit their discussion of ion migration to halide ions (anions) and do not specifically address the migration of cations (Cs, Pb). Naturally, halide migration is the primary focus when Cl and Br cross the interface. However, to understand the degradation mechanism at the interface, cation migration must also be considered. Initially, a gap exists at the interface. Over time, this gap is partially closed by nanobridges. These nanobridges ultimately consist of crystalline perovskite material, so the initial local cation depletion in the gap must have been compensated for by cation migration. The experimental results (Fig. S6) and numerical simulations (Fig. 5, in particular Cs seems mobile) support this interpretation. The manuscript would gain significantly in clarity if both halide diffusion and cation migration were discussed.

Furthermore, it can be observed that despite the excess of halide ions in the gap, diffusion into the bulk is limited. Halide ions seem to leak out from bulk into the gap, but a diffusion barrier may exist between the gap and the bulk leading to the halide excess concentration in the gap. Hence, contrary to the authors' description, the gap therefore does not really function as an effective reservoir for halide interdiffusion. The effective reservoir thus remains the bulk itself. However, it is only accessible for interdiffusion after a crystalline bridge across the gap has formed. The manuscript would would gain significantly in clarity if bulk-bulk diffusion and surface-bulk diffusion are clearly distinguished.

One more comment to the 3-step explanation provided by the authors: “(1) … ion migration is dominated by concentration gradients”. In my understanding, diffusion always requires concentration gradients. The question is rather, what determines the diffusion constant?

Suggestions for a modified explanation:

1. The experimental results show that halide interdiffusion between bulk components is significantly more efficient in the absence of a gap. As long as a gap exists, i.e., clearly separated surfaces are present, halide interdiffusion is restricted.
    - Maybe not surprising, but there is an obvious asymmetry in the diffusion direction and the manuscript would gain clarity if this could be stated with a quantitative estimate, i.e. to compare the diffusion barrier from the gap to bulk with the diffusion barrier from the bulk into the gap.

2. For efficient halide interdiffusion, it is necessary to close the gap. This is initiated by the formation of nano-bridges for which “slow” cation migration is crucial. The role of cation migration should be clearly explained. Apparently, nano-bridge formation is triggered by the interaction with the neighbouring crystal and dependent on the strength of those interactions. Interestingly, this interaction can be tuned (locally) by the twist angle.
    - As an extreme case of a non-interacting interface, a single crystal (and single surface) could be considered. In this case, ion “surface” migration should be even less than in the case of 28°. The authors may consider to include this in SI.

3. In this context, Fig S3 seems so important that it would be justified to place it into the main text. Only fig S3 clearly shows, how the halide interdiffusion bursts out locally once a nano-bridge is formed. This is an absolutely crucial result which is not visible in current fig 2 (see also comments below). Hence, I suggest an alternative fig 2:

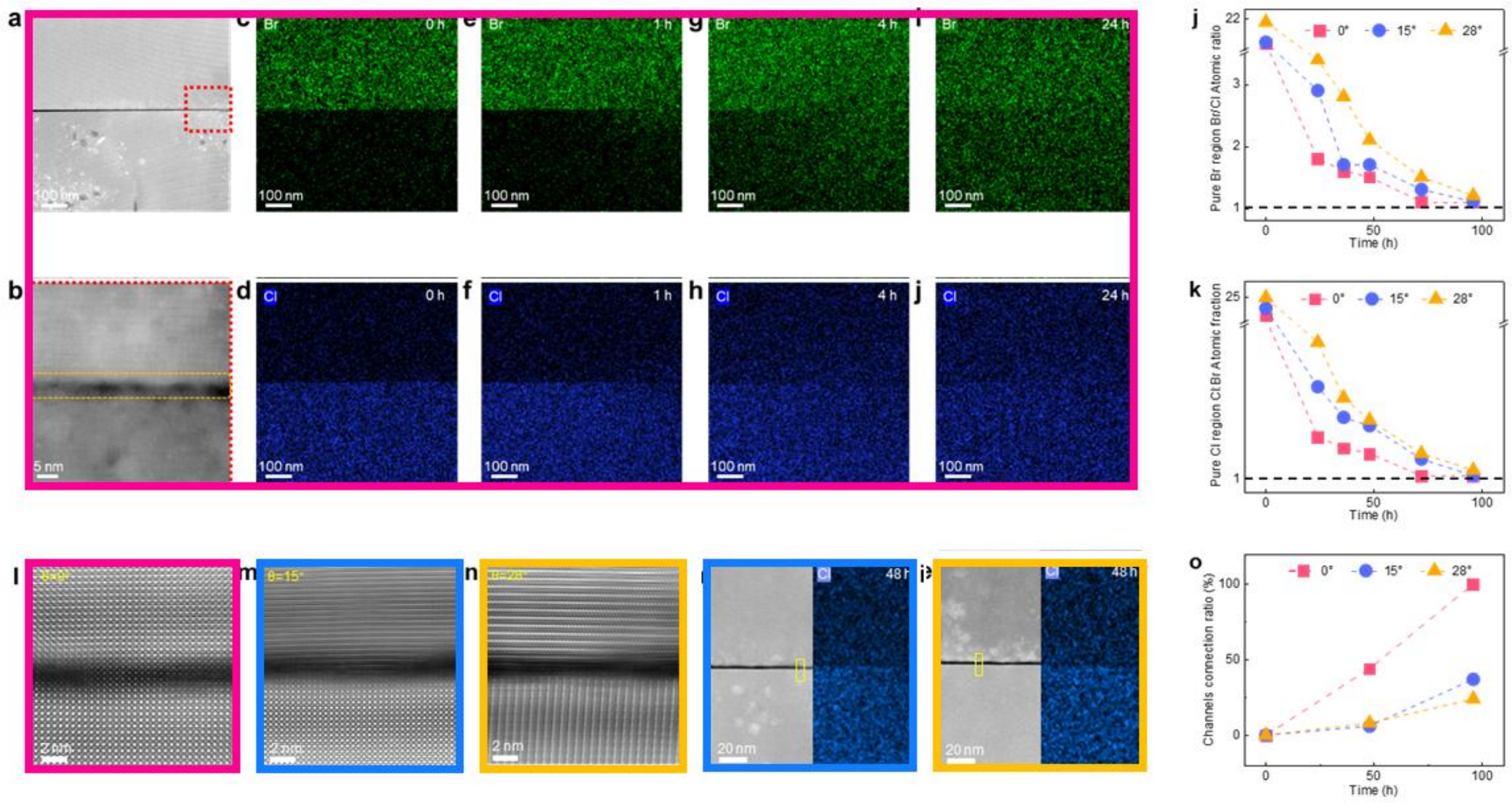

**Twist angle dependence**

4. According to fig 4h, MD simulations were performed for twist angles 0°, ≈ 18°, ≈ 28°, ≈ 35°, and 45°. It would be good, if the authors could state the exact angles. Notably, the experimental twist angle of 15° is missing and it is surprising that the experimental twist angle has not been considered in MD simulations. Could the authors explain and justify the particular set of twist angles used in MD simulations? Periodic boundary conditions..?
    - As a side note, it may help the reader to explicitly state that for cubic symmetry the range of twist angles 0° - 45° is sufficient to investigate.

5. What is so special about 28°? From crystal symmetry (cubic) this is not obvious. While 28° degrees do not coincide with any symmetry axis of the considered crystals, it might match with coincident site lattice (CSL). With the simulations at hand, the authors should provide some scientific insight.

**Smaller comments:**

Page 4: “Recently, twistionics, the deliberate control …”

Here, it is about twistronics. Please correct.

Page 6: Discussion of twist-angle-dependent interdiffusion of halide ions, fig 2a-c).

The start of the discussion does not clearly state, that this is for twist angle 0°. Please make this more clearly.

Page 7: “meanwhile, statistical analysis …”

What kind of analysis? From experimental TEM data or numerical simulations? Please clarify.

Page 7: "… aligned and unaligned feature, as indicated in Fig S7… diffusion channels prefer to be formed where the lattice … aligns."

Whether the lattices are aligned or misaligned is not clearly visible in the figure. Further, fig S7 claims that half a unit cell misalignment also yields efficient ion migration? Maybe, the authors could find a better way to present the experimental results. See also comment to Fig S7 below.

Page 8: "Considering that lattice mismatch induced by twist angle …"

This sounds a bit confusing, because "mismatch" associates with mismatch of lattice parameter. Maybe one could write "lattice misalignment"?

Page 8, ref [40]

Is this the correct reference for ion migration? Ref 40 "DFT study on type-II photocatalyst for overall water splitting …" discusses only electronic charge carrier migration and does not mention ion migration at all.

Page 9, "For the 0° device, it is observed a significant rectifying behaviour…"

Language! Please improve.

Fig1, caption: "(c) Optical images of twisted **the** heterostructures …"

Typo, please skip the "the".

Fig2,

6. Yellow boxes to indicate diffusion channels: On that scale and resolution, we simply cannot identify diffusion channels or nano-bridges. Maybe, a high-resolution image would show them, but on the chosen larger range the reader cannot see those. I do not think that inserting "yellow boxes" as a guide to the eye is the way to go... Anyway, I suggest to rework fig 2, see above.
7. Caption: false enumeration (It is correct in the text): (m,n) => (j,k), (j-l) => (l-n)
8. Text within sub-figures is very small. When text is placed on non-uniform background, such as atomic resolution TEM, then one cannot read the text without significant enlargement. Well, this is possible only in the digital version…

Fig3, also same problem with text as in fig2: too small…

Fig4:

- It would help if row d-f could be separated more clearly from row a-c. Currently, the gap with the halide heterostructure is similar as the gap between figure a and figure d! This makes it difficult to read.

Fig 5

- Fig 5a): What is the little doted square? Please clarify or remove.

- The $CsPbCl_3$-film is grown on STO as described in methods. But fig 5b) c) actually indicate that there are $CsPbBr_3$- flakes and $CsPbCl_3$-flakes. A double transfer process is not described in the methods section, and should probably be completed, accordingly. Yet, it may suggest that the substrate for the devices is indeed something else than STO. Probably not only Si, but $SiO_2$/Si? Please clarify and correct accordingly.
  The $CsPbCl_3$ film is deposited onto STO as described in the methods. However, Figures 5b) and c) show $CsPbBr_3$ and $CsPbCl_3$ flakes. A double transfer process is not described in the methods and should therefore be added. Further, this suggests that the substrate of the devices is not the original growth substrate STO, and it could be Si as indicated in the figure. Yet, I suspect it is rather $SiO_2$/Si. Please clarify.

- The dotted lines are probably supposed to highlight the edges of the $CsPbBr_3$-flake. I would prefer to skip the dotted lines since this just obscures the optical image and gives a false impression. For example, the dotted boxes suggests that the flake is rectangular while the image shows it is not perfectly rectangular. If it is really necessary to highlight the $CsPbBr_3$-flake, one could opt for false colour.

Supplementary material

Fig S2:

- It would improve readability if the separate lines would not overlap. Maybe, one could consider separate graphs for each atomic species?

Fig S3-8

- Please indicate the twist angle! I assume it is 0°, but it is nowhere clearly stated.

Fig S4:

- a) Please skip the dotted boxes.
- b) Please skip the dotted lines. It seems they are just qualitative guides to the eye, which do not add really anything.
- b) Please skip the orange dotted line. Again, this is just a guide to the eye. This time, without to explain the criteria, i.e. 50% Br? Please, either explain clearly how this line is defined, or skip. Even without guides to the eye the main message is clear: the gap is closing and a crystalline interface is formed, allowing for efficient halide interdiffusion.

Fig S7

- a) – c): Aligned and unaligned regions are marked, but the reader has no chance to independently verify this: too small image, too small range …
- (g-i) Shift of half a unit cell is not clearly visible. In any case, does it mean, that also an unaligned lattice forms nanobridges?
- (g-i) What does APB mean? Please explain.
